# Genome-wide expression patterns associated with oncogenesis and sarcomatous transdifferentation of cholangiocarcinoma

**DOI:** 10.1186/1471-2407-11-78

**Published:** 2011-02-19

**Authors:** Min-A Seol, In-Sun Chu, Mi-Jin Lee, Goung-Ran Yu, Xiang-Dan Cui, Baik-Hwan Cho, Eun-Kyung Ahn, Sun-Hee Leem, In-Hee Kim, Dae-Ghon Kim

**Affiliations:** 1 Division of Gastroenterology and Hepatology, the Institute for Medical Science, Departments of Internal Medicine, Chonbuk National University Medical School and Hospital, Jeonju, Jeonbuk, South Korea; 2 Department of Surgery, Chonbuk National University Medical School and Hospital, Jeonju, Jeonbuk, South Korea; 3 Medical Genomics Research Center, Korea Research Institute of Bioscience and Biotechnology, Daejeon 305-806, South Korea; 4 Department of Biological Science, Dong-A University, Busan 604-714, South Korea

## Abstract

**Background:**

The molecular mechanisms of CC (cholangiocarcinoma) oncogenesis and progression are poorly understood. This study aimed to determine the genome-wide expression of genes related to CC oncogenesis and sarcomatous transdifferentiation.

**Methods:**

Genes that were differentially expressed between CC cell lines or tissues and cultured normal biliary epithelial (NBE) cells were identified using DNA microarray technology. Expressions were validated in human CC tissues and cells.

**Results:**

Using unsupervised hierarchical clustering analysis of the cell line and tissue samples, we identified a set of 342 commonly regulated (>2-fold change) genes. Of these, 53, including tumor-related genes, were upregulated, and 289, including tumor suppressor genes, were downregulated (<0.5 fold change). Expression of SPP1, EFNB2, E2F2, IRX3, PTTG1, PPARγ, KRT17, UCHL1, IGFBP7 and SPARC proteins was immunohistochemically verified in human and hamster CC tissues. Additional unsupervised hierarchical clustering analysis of sarcomatoid CC cells compared to three adenocarcinomatous CC cell lines revealed 292 differentially upregulated genes (>4-fold change), and 267 differentially downregulated genes (<0.25 fold change). The expression of 12 proteins was validated in the CC cell lines by immunoblot analysis and immunohistochemical staining. Of the proteins analyzed, we found upregulation of the expression of the epithelial-mesenchymal transition (EMT)-related proteins VIM and TWIST1, and restoration of the methylation-silenced proteins LDHB, BNIP3, UCHL1, and NPTX2 during sarcomatoid transdifferentiation of CC.

**Conclusion:**

The deregulation of oncogenes, tumor suppressor genes, and methylation-related genes may be useful in identifying molecular targets for CC diagnosis and prognosis.

## Background

Cholangiocarcinoma (CC) is a highly lethal adenocarcinoma arising from bile duct epithelial cells. CC accounts for approximately 15% of the total liver cancer cases worldwide, and its incidence is rising [1,2]. The prognosis for CC is quite poor because of difficulties in early diagnosis, and relative resistance of the tumors to chemotherapy [3,4]. At the time of diagnosis, approximately 70% of CC patients have an occult metastasis or advanced local disease that precludes curative resection. Of candidates for curative resection, 30% develop recurrent disease at the anastomotic site or within the intrahepatic biliary tree, and succumb to disease progression or cholangitis [5]. Established risk factors for ductal cholangiocarcinomas include primary sclerosing cholangitis, infection with *Clonorchis sinensis *or *Opisthorchis viverrini *(liver flukes), Calori's disease, congenital choledochal cysts, and chronic intrahepatic lithiasis [6]. However, for most CCs, the cause is unknown.

Recently, molecular investigations have provided evidence that CC carcinogenesis involves a number of genetic alterations, including activating point mutations in the *K-ras *oncogene, and in *p53 *and *BRAF *[7-9]. The deregulated expression of a number of other genes has also been reported, and cyclooxygenase-2 and c-erbB-2 are frequently overexpressed in CCs, suggesting an involvement in early biliary carcinogenesis [10]. In addition, increased expression of interleukin-6 is frequently observed in CC [11]. CC also develops after the liver-specific targeted disruption of the tumor suppressors *SMAD4 *and *PTEN *[12]. The incidence of sarcomatoid changes in CC is estimated to be approximately 5% [13], and sarcomatoid cells are thought to result from de-differentiation of ordinary carcinomatous CC cells. Sarcomatoid neoplasms are highly aggressive and have a poorer survival rate than ordinary CCs [14], but the underlying molecular alterations, which may be related to the epithelial-mesenchymal transition (EMT), remain unclear. Little extensive genome-wide information about altered gene expression in CCs is available, and only a few published studies have reported a comprehensive analysis of gene expression among biliary tract cancers in general [15,16]. The advancement of microarray technology now enables us to analyze genome-wide gene expression in a single experiment, opening avenues for the molecular classification of tumors, detection of the biological nature of tumors, and prediction of prognosis and sensitivity to treatments.

In this study, we generated genome-wide gene expression profiles of 10 cell lines (9 CC cell lines and 1 immortalized cholangiocyte line), and 19 CC tissues using a BeadChip oligonucleotide technology containing 48,000 genes. This procedure allowed us to observe a comprehensive pattern of gene expression in CC compared to cultured normal biliary epithelia (NBE). In addition, we identified a set of genes associated with sarcomatoid transdifferentiation. These data are useful not only because they provide a more profound understanding of cholangiocarcinogenesis and transdifferentiation, but also because they may help to develop diagnostic tools and improve the accuracy of CC prognosis.

## Methods

### Cell lines and cultures

Tumor tissues were obtained from surgical specimens and biopsy specimens in Korean cholangiocarcinoma patients. Tumor tissues were washed three times in Opti-MEM I (Gibco, Grand Island, NY) containing antibiotics. Washed tissue was transferred to a sterile Petri dish and finely minced with scalpels into 1- to 2-mm^3 ^fragments. Tissue fragments in culture medium were seeded in T25 culture flasks (Corning, Medfield, MA) in Opti-MEM supplemented with 10% fetal bovine serum (FBS, Gibco), 30-mM sodium bicarbonate and antibiotics. Tumor cells were cultured undisturbed and passaged as described [17]. Near the 20th passage, the medium was changed from Opti-MEM I to DMEM supplemented with 10% FBS and antibiotics. NBE cells were isolated from mucosal slices of normal bile ducts, with informed consent from liver transplantation donors, and *ex-vivo *cultured in T25 culture flasks in Opti-MEM supplemented with 10% FBS, 30 mM sodium bicarbonate and antibiotics at 37°C with 5% CO_2 _in air. Near-confluent NBE cells were harvested and stored at -80°C until use. Cells were routinely tested for mycoplasma and found to be negative using a Gen Probe kit (San Diego, CA). CC cell lines are in Table 1.

**Table 1:** Clinicopathological features of nine patients with intrahepatic cholangiocarcinomas used to generate CC cells lines.

Patient No	Cell line	Age/Sex	T/N/M Stage	CA19-9 (U/ml)	Differentiation	Tumori genicity	Comments
1	CK-Choi (Choi-CK)	M/68	IVB	184	WD	+	
2	CK-Cho (Cho-CK)	M/82	IVA	500	MD	+	
3	CK-J (JCK)	M/72	IVA	125	PD	+	*C. sinensis*
4	CK-S (SCK)	M/68	IVA	235.6	PD	+	Sarcomatoid
5	CK-L1	M/46	IVA	0.01	PD	+	Combined with HCC
6	CK-L2	M/65	III	2050.1	MD	+	
7	CK-P1	M/66	IVA	23.7	MD	-	
8	CK-P2	F/66	IVA	121.4	MD	+	
9	CK-Y1	M/52	IVA	0.01	PD	+	Combined with HCC

M, male; F, female; C. sinensis, clonorchis sinensis; HCC, hepatocellular carcinoma; WD, well differentiated; MD, moderately differentiated; PD, poorly differentiated.

*International Hepato-Pancreato-Biliary Association classification.

### 5-Aza-2'-deoxycytidine (Aza) treatment

Choi-CK, Cho-CK, and JCK cells were seeded at 1 × 10^6 ^cells/ml. After overnight culture, cells were treated with 5 μM of the DNA methylating agent Aza (Sigma-Aldrich, St. Louis, MO) for 4 days, and then harvested.

### Patients and tissue samples

CC tissues were obtained with informed consent from Korean patients who underwent hepatectomy and common bile duct exploration at Chonbuk National University Hospital. All tumors were clinically and histologically diagnosed as cholangiocarcinoma. Detailed clinocopathological data of the 19 samples are in Table 2. All samples were immediately frozen in nitrogen tanks. Patient information was obtained from medical records. Clinical stage was determined according to the International Hepato-Pancreato-Biliary Association (IHPBA) classification [18].

**Table 2:** Clinicopathological features of 19 CC samples used for microarray analysis.

Sample No	Age/Sex	Loca tion	Size (cm)	T	N	M	Stage	Gross appearance	Differentiation	Sub-class	Comment
1 (CC-GHS)	68/F	L	8.7 × 5.4	3	1	0	IVA	MF	PD	A	
2 (CC-CYS)	57/M	L	NA	3	1	0	IVA	MF +PDI	MD	A	
3 (CC-LJS)	42/M	A	NA	1	0	0	I	ID	WD	B	Intraductal papillary
4 (CC-BJP)	62/M	P	7.8 × 5.6	1	0	0	I	ID	WD	B	Intraductal papillary
5 (CC-HSR)	66/M	AP	7.3 × 6	2	0	0	II	MF	MD	B	
6 (CC-HSW)	59/M	AP	9 × 6.8	2	0	0	II	MF	MD	B	
7 (CC-CSB)	60/M	L	4 × 4.5	3	0	0	III	MF	MD	B	
8 (CC-SJS)	71/M	A	2.1 × 1.9	1	0	0	II	MF	WD	B	
9 (CC-HDS)	63/M	CBD	1.1 × 0.9	1	1	1	IVB	ID	MD	B	
10 (CC-KHC)	47/M	L	14 × 10	4	0	0	IVA	MF	PD	C	
11 (CC-LHG)	42/M	L	5.6 × 3.9	3	1	0	IVA	MF	PD	C	Combined with HCC
12 (CC-LSH)	40/F	P	8.6 × 4	2	0	1	IVB	MF	PD	C	Combined with HCC
13 (CC-KHS)	70/F	L	8.5 × 4.8	3	0	1	IVB	MF +PDI	PD	C	Combined with HCC
14 (CC-LMS)	38/F	AP	5 × 3.7	4	0	1	IVB	MF	PD	C	Combined with HCC
15 (CC-KJA)	39/F	LP	6 × 5	4	0	0	IVA	MF	MD	C	Combined with HCC
16 (CC-JSJ)	64/M	L	0.5 × 0.5	2	0	1	IVB	ID	PD	C	
17 (CC-YCU)	53/M	L	4.3 × 2.1	3	1	0	IVA	MF	MD	C	
18 (CC-GMG)	50/M	L	7 × 3.5	2	0	0	II	MF	MD	C	
19 (CC-BSD)	67/M	L	2.9 × 2.7	4	0	0	IVA	MF+P야	PD	C	

HCC, hepatocellular carcinoma; M, male; F, female; A, anterior segment; P, posterior segment; Med, medial segment; L, lateral segment; MF, mass forming type; PDI, periductal infiltrating type; IDG, intraductal growth type; WD, well differentiated; MD, moderately differentiated; PD, poorly differentiated; NA, not available.

*International Hepato-Pancreato-Biliary Association classification.

### Primer labeling and Illumina Beadchip array hybridization

Total RNA from CC samples was isolated using TRIzol reagent (Invitrogen, CA) according to the manufacturer's instructions. RNA quality was determined by gel electrophoresis, and concentrations were determined using an Ultrospec 3100 pro spectrophotometer (Amersham Bioscience, Buckinghamshire, UK). Biotin-labeled cRNA samples for hybridization were prepared according to Illumina's recommended sample-labeling procedure: 500 ng of total RNA was used for cDNA synthesis, followed by an amplification/labeling step (*in vitro *transcription) to synthesize biotin-labeled cRNA using the Illumina TotalPrep RNA Amplification kit (Ambion Inc., Austin, TX). cRNA concentrations were measured by the RiboGreen method (Quant-iT RiboGreen RNA assay kit; Invitrogen-Molecular Probes, ON, Canada) using a Victor3 spectrophotometer (PerkinElmer, CT), and cRNA quality was determined on a 1% agarose gel. Labeled, amplified material (1500 ng per array) was hybridized to Illumina Human-6 BeadChips v2 containing 48,701 probes for 24,498 genes, according to the manufacturer's instructions (Illumina, San Diego, CA). Array signals were developed by Amersham fluorolink streptavidin-Cy3 (GE Healthcare Bio-Sciences, Little Chalfont, UK) following the BeadChip manual. Arrays were scanned with an Illumina Bead-array Reader confocal scanner (BeadStation 500GXDW; Illumina) according to the manufacturer's instructions. Array data processing and analysis were performed using Illumina BeadStudio software. The BeadStudio Gene Expression Module is a tool for analyzing gene expression data from scanned microarray images generated by the Illumina BeadArray Reader.

### Data analysis

Normalization algorithms were used to adjust sample signals to minimize the effects of variation from non-biological factors. To reduce variation between microarrays, the intensity values for samples in each microarray were rescaled using a quartile normalization method in the BeadStudio module. Measured gene expression values were log2-transformed and median-centered across genes and samples for further analysis. To generate an overview of the gene expression profile and to identify major relationships in cell lines, we used unsupervised hierarchical clustering analysis. Genes with an expression ratio of at least a two-fold difference relative to the median gene expression level across all samples in at least 10% of samples were selected for clustering analysis. Average linkage hierarchical cluster analysis was carried out using a Pearson correlation as the similarity metric, using the GeneCluster/TreeView program (http://rana.lbl.gov/EisenSoftware.htm). Expression profiles for the differentially expressed genes were selected by *t*-test with false discovery rate (FDR) and q-values as gene significance measures, using R software (version 2.5). Because of varying significance in the analyzed comparisons, using a fixed FDR (or q-value) cut-off value was not practical. Therefore, we used *t*-test *P *= 0.01. To ascertain biological relevance, a fold-change cut-off value of 2 or 4 from the mean was chosen. The gene ontology (GO) program (http://david.abcc.ncifcrf.gov/) was used to categorize genes in subgroups based on biological function. Values for each GO group were calculated as a percentage of total mRNA change. For example, the Fisher exact test was used to determine whether the proportions of genes in each category differed by group. The microarray data were registered with the Gene Expression Omnibus (GEO) database (Accession No. GSE22633)

### Immunoblotting

Extracted protein (30 μg) from cell lysates was resolved by SDS-PAGE and transferred to a nitrocellulose membrane. Membranes were incubated for 1 h at room temperature with primary antibody at 1:1000 dilution. After incubation, blots were washed three times in TBS/0.1% Tween 20. Immunoreactivity was detected using alkaline phosphatase-conjugated goat anti-rabbit IgG or a commercial chemiluminescence detection kit (Amersham), according to the manufacturer's instructions.

### Immunohistochemistry

Immunohistochemical staining was performed on formalin-fixed, paraffin-embedded 4-μM tissue sections, as described preciously [19]. Briefly, a deparaffinized section was pretreated by microwave epitope retrieval (750 W during 15 min in citrate buffer 10 mmol; pH 6.0) after rehydration. Before applying primary antibodies, the endogenous peroxidase activity was inhibited with 3% hydrogen peroxide, and a blocking step with biotin and bovine albumin was performed. Primary monoclonal or polyclonal antibodies were detected using a secondary biotinylated antibody and a streptavidin-horseradish peroxidase conjugate according to the manufacturer's instructions (DAKO, Glostrup, Denmark). Counterstaining was performed using Meyer's hematoxylin. Tumors were evaluated for the percentage of positive cells and the staining intensity. Negative controls were samples incubated with either PBS or mouse IgG_1 _instead of primary antibody.

### Real-time RT-PCR

RNA prepared from dissected tissues was precipitated with isopropanol and dissolved in DEPC-treated distilled water. Reverse transcription (RT) was performed using 2 μg total RNA, 50 μM decamer and 1 μl (200 units) and RT-PCR Superscript II (Invitrogen) at 37°C for 50 min, as previously described. Specific primers for each gene were designed using the Primerdepot website (http://primerdepot.nci.nih.gov/) and are in Additional file 1. The control *18S *ribosomal RNA primer was from Applied Biosystems (Foster City, CA) and was used as the invariant control. The real-time RT-PCR reaction mixture consisted of 10 ng reverse-transcribed total RNA, 167 nM forward and reverse primers, and 2 × PCR master mixture in a final volume of 10 μl PCR, was in 384-well plates using the ABI Prism 7900HT Sequence Detection System (Applied Biosystems).

### Animal model of cholangiocarcinoma

The hamster CC model was modified from a previous study [20]. On the first day of the experiment, hamsters in the experimental group were infected with 15 metacercariae of the liver fluke, *C. sinensis*. One day after parasite infestation, hamsters received 15 ppm of dimethylnitrosamine (DMN; Kasei, Japan) in the drinking water for 4 weeks with a normal diet. Thereafter, hamsters were given tap water with a normal diet for the rest of the study. An interim stage of cholangiocarcinogenesis was confirmed at 8 weeks after experiment initiation. Control and CC model hamsters were maintained for a total of 27 weeks for CC to develop.

## Results

### Gene expression patterns distinguish CC cells from cultured NBE cells

**Figure 1:**
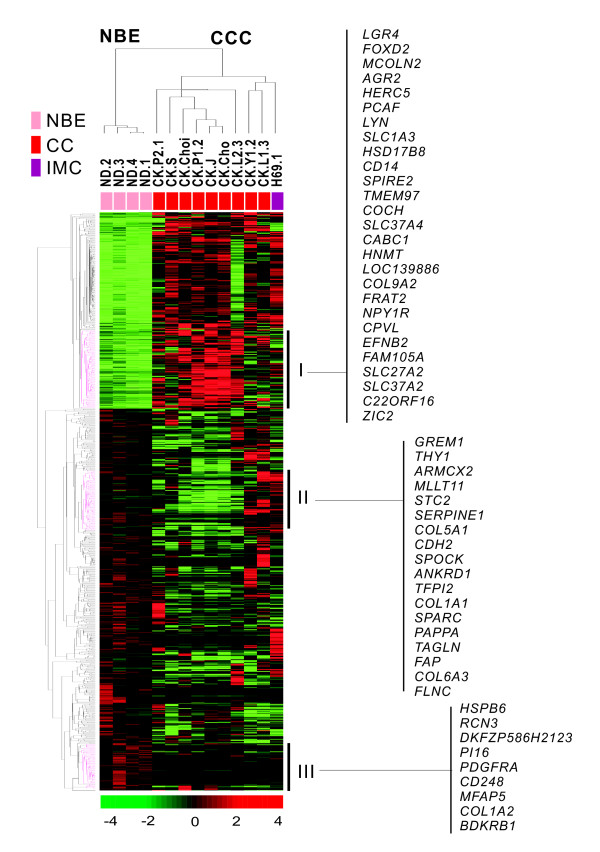
**Unsupervised hierarchical clustering of four biliary epithelial cells, one immortalized cholangiocyte cell line and nine CC cells**. Unsupervised hierarchical clustering separated the samples into two main groups, normal biliary epithelial cells (NBE) isolated from mucosal slices of normal bile ducts and *ex-vivo *cultured as described in Methods, and cholangiocarcinoma cells (CCC). Data are in matrix format, with columns representing individual cell lines and rows representing each gene. Red, high expression; green, low expression; black, no significant change in expression level between the mean and sample. A hierarchical clustering algorithm was applied to all cells and genes using the 1 - Pearson correlation coefficient as a similarity measure. Raw data for a single array were summarized using Illumina BeadStudio v3.0 and output to the user was as a set of 43,148 values for each individual hybridization. We selected 828 unique genes with a two-fold or greater difference from the mean and *P *< 0.01 by *t*-test, for hierarchical clustering analysis. Specific gene clusters (Cluster 1 through Cluster III) were identified in the hierarchical cluster of the genes differentially expressed in CCC compared with NBE. CC, cholangiocarcinoma; IMC, immortalized cholangiocytes.

Using BeadChip microarray analysis, we compared the gene expression profiles of nine CC cell lines, an immortalized biliary epithelial cell line, and four types of NBE cells. We selected 828 unique genes with a 2-fold or greater expression difference from the mean, with a *P *< 0.01 by *t*-test. Unsupervised hierarchical clustering analysis of all samples was based on the similarity in the expression pattern of all genes (Figure 1). Cell samples were separated into two main groups, the NBE cluster, and the transformed and immortalized biliary epithelial cells (CCC cluster). Each distinctive gene cluster was identified by delineation using a hierarchical clustering dendrogram. Cluster I consisted of genes upregulated in CC cells, which included tumor-related genes such as *LGR4, AGR2*, *PCAF, TMEM97, FRAT2*, *EFNB2 *and *ZIC2 *[21-27]. Cluster II included genes underexpressed in CC cells. These were mainly tumor suppressor genes such as *GREM1, THY1, STC2, SERPINE1, SPARC *and *TAGLN *[28-33]. Cluster III was genes upregulated in NBE cells, and contained the *PDGFRA*, *CD248*, and *BDKRB1 *genes.

### Gene expression patterns distinguish CC tissues from cultured NBE cells

**Figure 2:**
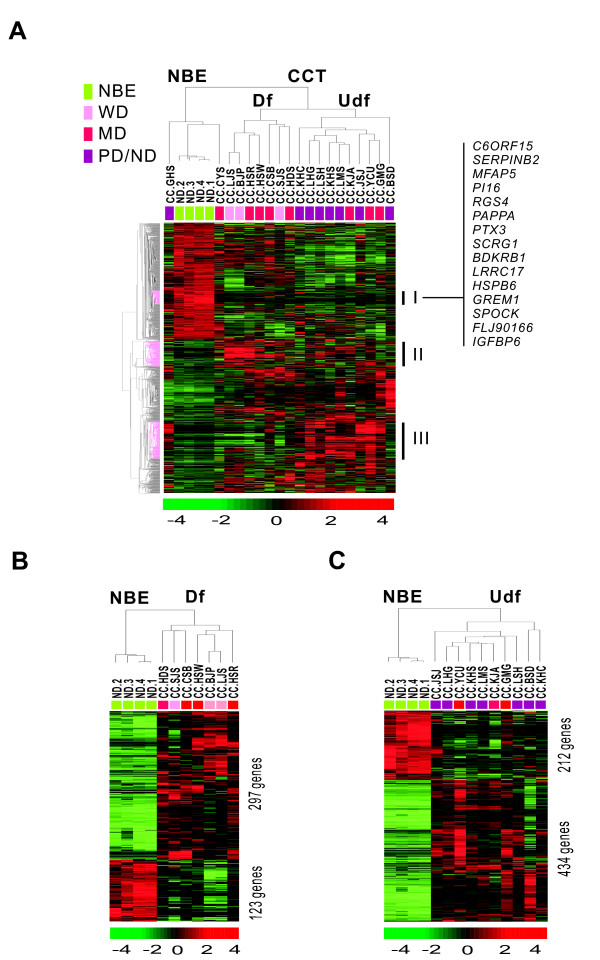
**Unsupervised hierarchical clustering of 4 biliary epithelial cells and 19 CC tissues**. (A) Unsupervised hierarchical clustering separated the samples into two main groups. We selected 1798 unique genes with two-fold or greater difference from the mean with *P *< 0.01 by *t*-test for hierarchical clustering analysis. Specific gene clusters (Cluster 1 through Cluster III) were identified of differentially expressed in CCT compared to NBE. (B) Supervised hierarchical clustering of four biliary epithelial cells and seven differentiated CC tissues. We selected 420 unique genes with four-fold or greater difference from the mean and *P *< 0.01 by *t*-test for hierarchical clustering analysis. (C) Supervised hierarchical clustering of 4 biliary epithelial cells and 10 undifferentiated CC tissues. We selected 646 unique genes with the criteria in B for hierarchical clustering analysis.

Using BeadChip microarrays, gene expression profiles of 19 CC tissues and 4 types of NBE cells were compared. We selected 1798 unique genes with a 2-fold or greater differences from the mean difference with a *P *< 0.01 by *t*-test. Unsupervised hierarchical clustering analysis was as described above (Figure 2A). All samples separated into two main groups, NBE and CC tissues (CCT). Each distinctive gene cluster was identified using a hierarchical clustering dendrogram as above. Intriguingly, the CC sample cluster was divided into two subclasses by tumor differentiation: differentiated (Df) and undifferentiated (Udf). Clustering data for the CC group revealed three clusters. Cluster I had genes upregulated in NBE and downregulated in CCT including *SERPINB2, PAPPA, LRRC17*, and *GREM1*. Cluster II contained genes upregulated in the Df CCT and downregulated in NBE. Cluster III included genes upregulated in poorly differentiated or Udf CCT, and downregulated in NBE. A supervised hierarchical clustering analysis was performed between the NBE class, and the Df and the Udf subclasses based on the similarity of expression pattern of all genes (Figure 2B and 2C). We selected 420 differentially expressed genes in the Df subclass, and 646 genes in the Udf subclass for comparison with the NBE class (Additional files 2 and 3).

### Differential expression and verification of CC-related genes

**Figure 3:**
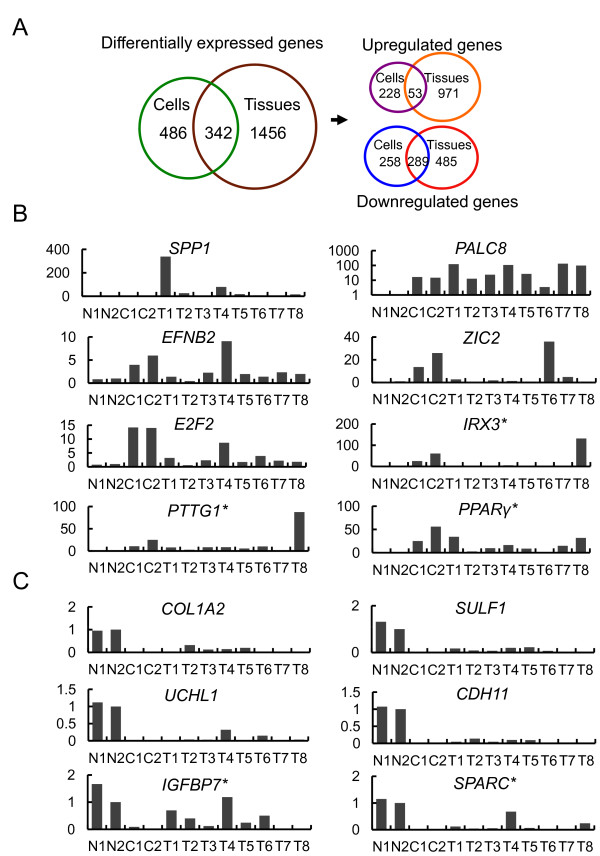
**Differentially regulated genes in human CC tissues compared to NBE cells**. (A) Venn diagram of genes commonly regulated in the cell and tissue samples. The 342 genes included 53 upregulated and 289 downregulated genes, selected from the cell- and tissue-based microarray databases. (B) Real-time RT-PCR analysis of upregulated genes selected from the list of top 25 genes commonly upregulated in both CC cells (C) and tissues (T) compared to cultured NBE cells (N). *, selected from only the cell-based microarray database. (C) Real-time RT-PCR analysis of downregulated genes selected from the list of top 25 genes commonly downregulated in both CC cells (C) and tissues (T) compared to cultured NBE cells (N). *, selected from only the cell-based microarray database.

We compared the gene lists from the cell-based and tissue-based databases, and selected 342 commonly regulated genes, including 53 commonly upregulated genes and 289 commonly downregulated genes (Figure 3A). The top 25 commonly regulated genes in both CCC and CCT compared to NBE are in Additional file 4. To verify the microarray data, we examined the mRNA levels of the identified genes using real-time RT-PCR in human CC tissues. We selected five up-regulated genes from the commonly upregulated genes of both the cell and tissue sample classes (Figure 3B). We also chose the *IRX3*, *PTTG1*, and *PPARγ *genes, which were highly upregulated in only the cell sample class. These genes were preferentially expressed in CC cells and tissues. We also examined the expression of the commonly downregulated *KRT17 *and *UCHL1 *genes, as well as the cellular downregulated *IGFBP7 *and *SPARC *genes using real-time RT-PCR in human CC. The human NBE showed substantial expression of *CK-17*, *UCHL1*, *IGFBP7* and *SPARC*, which were barely detected in CC tissues (Figure 3C).

### Immunohistochemical analysis of CC-related genes

**Figure 4:**
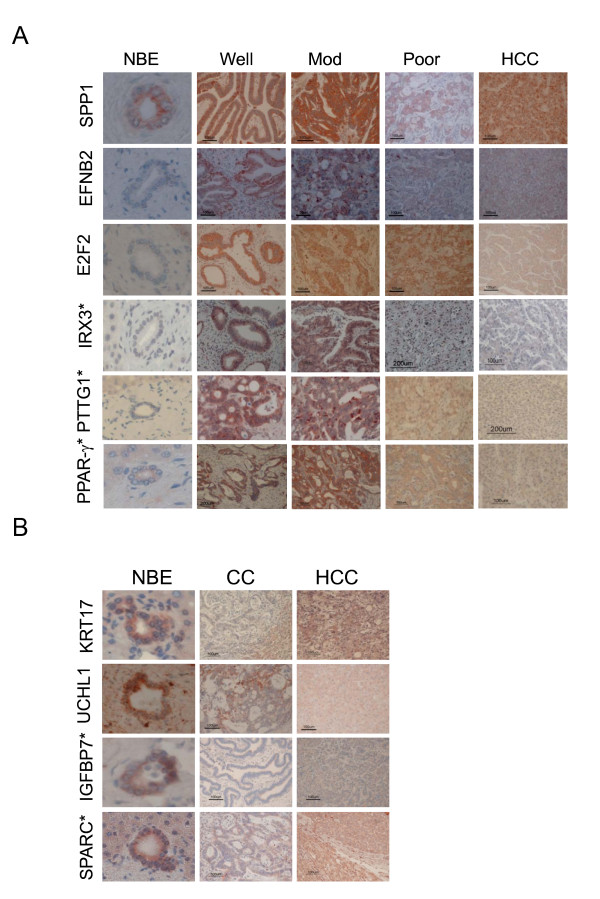
**Immunohistochemical staining of differentially expressed proteins in the CC tissues**. (A) Immunohistochemical staining with anti-SPP1, anti-EFNB2, anti-E2F2, anti-IRX3, anti-PTTG1 or anti-PPARγ in NBE, human CC tissues with good differentiation (well), moderate differentiation (mod) or poor differentiation (poor), and HCC tissues. The representative genes were selected from the list of top 25 commonly upregulated genes, according to antibody available for immunohistochemistry. *, selected from only cell-based microarray database. The representative picture is one of three immunohistochemical staining replicates of different specimens. (B) Immunohistochemical staining with anti-KRT-17, anti-UCHL1, anti-IGFBP7 or anti-SPARC in the human CC and HCC tissues. *, selected from only cell-based microarray database. The representative picture is one of three immunohistochemical staining replicates of different specimens

To confirm the reliability of the microarray data and the robustness of the strategy for identifying genes with altered expression, we examined the protein levels of the identified genes using immunohistochemical analysis of human tissues (Figure 4A). We selected three upregulated genes from the genes that were upregulated in both cell and tissue samples. The SPP1, EFNB2 and E2F2 proteins were abnormally overexpressed in the CC cell cytoplasm, and weakly or barely expressed in HCC. We also examined the IRX3, PTTG1, and PPARγ proteins, which were highly upregulated in only the cell samples. IRX3 was the most highly upregulated, and we was strongly expressed in the nucleus of CC cells in the tissue sections, but was barely detectable in the NBE nuclei. PTTG1 and PPARγ were abnormally overexpressed in the CC cell cytoplasm, and their expression was attenuated in poorly differentiated CC. Next, we also used immunohistochemical staining of human CC to examine the KRT17 and UCHL1 proteins, whose genes were both downregulated in CC cells and tissues, and the IGFBP7 and SPARC proteins, which were downregulated in CC cells only. Human NBE showed substantial expression of the CK-17, UCHL1, IGFBP7, and SPARC proteins, but these were barely detectable in CC tissue. However, KRT-17 was clearly positive in HCC (Figure 4B).

### Immunohistochemical analysis in hamster model of CC

Although it is unknown whether antibodies raised to human proteins recognize hamster proteins, we examined the protein levels of the identified genes using immunohistochemical analysis of hamster CC tissues (Additional file 5). As in humans, the SPP1, EFNB2, and E2F2 proteins were abnormally overexpressed in the hamster CC cell cytoplasm. IRX3 was also similarly expressed in the CC cell nucleus, and PTTG1 was differentially expressed in the CC cell cytoplasm. Interestingly, in contrast to human CC cells, PPARγ was preferentially expressed in the hamster CC cell nuclei. Therefore, the immunoreactivity of identified gene proteins in hamster CC seemed to be substantially consistent with that in human CC.

### Gene expression patterns distinguish the SCK cell line from three CC cell lines

**Figure 5:**
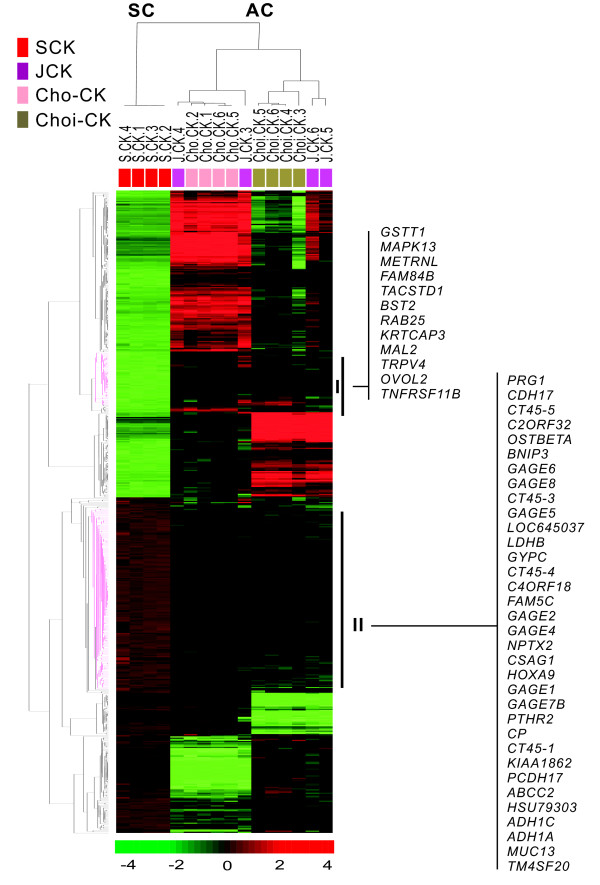
**Unsupervised hierarchical cluster analysis of differentially expressed genes illustrated in a heat-map**. Unsupervised hierarchical clustering separated the samples into two main groups: SC and AC. The samples were independently prepared from the cultured cells four times, and four kinds of CC cells were used: Choi-CK, Cho-CK, JCK and SCK cells. Samples were clustered closer within their own group than in samples from other groups. We selected 559 unique genes with a four-fold or greater difference from the mean and a *P *< 0.005 by *t*-test for hierarchical clustering analysis. Cluster I included genes differentially downregulated in the sarcomatoid CC cells compared to three adenomatous CC lines. Cluster II contained genes differentially upregulated in the sarcomatoid CC cells compared to three adenomatous CC lines.

Previously, we established four human CC cell lines and characterized one with a typical sarcomatoid phenotype of SCK. We classified the other cell lines according to tumor cell differentition, as a poorly differentiated JCK, a moderately differentiated Cho-CK, and a well-differentiated Choi-CK cell line [34]. Two-way unsupervised hierarchical clustering analysis of quadruplicate samples for each cell line was conducted, based on the similarity of expression patterns of all genes (Figure 5). We selected 559 unique genes whose expression differed from the mean by four-fold or more with *P *< 0.005 by *t*-test. Cell samples were separated into two main groups, sarcomatoid (SC) and ordinary or adenocarcinomatous CC (AC), by the gene axis. The SC group contained 292 differentially upregulated genes (>four-fold change), and 267 downregulated genes (<0.25-fold change), compared to the AC group. The top 25 genes that were differentially expressed in the sarcomatoid SCK cells compared to the three adenocarcinomatous CC lines are in Additional file 6. Clustering data within groups revealed that the core clusters I and II were associated with transdifferentiation. Genes in cluster I appeared to be downregulated in the SCK cells, compared to ordinary CC cells. In contrast, the genes in cluster II were upregulated in the SCK cells and downregulated in the ordinary CC cells. Cluster I contained the *GSTT1*, *TACSTD*, *BST2*, *RAB25*, and *MAL2 *genes. Cluster II contained genes associated with tumor progression and metastasis, including *HOXA9, MUC13*, and members of the *GAGE *and *CT-45 *families [35-38]. Expression of methylation-silenced genes, such as *LDHB, BNIP3, UCHL1*, and *NPTX2 *[39-42], was barely detectable in the AC group, but appeared in this cluster.

### Expressions of transdifferentiation-related genes

**Figure 6:**
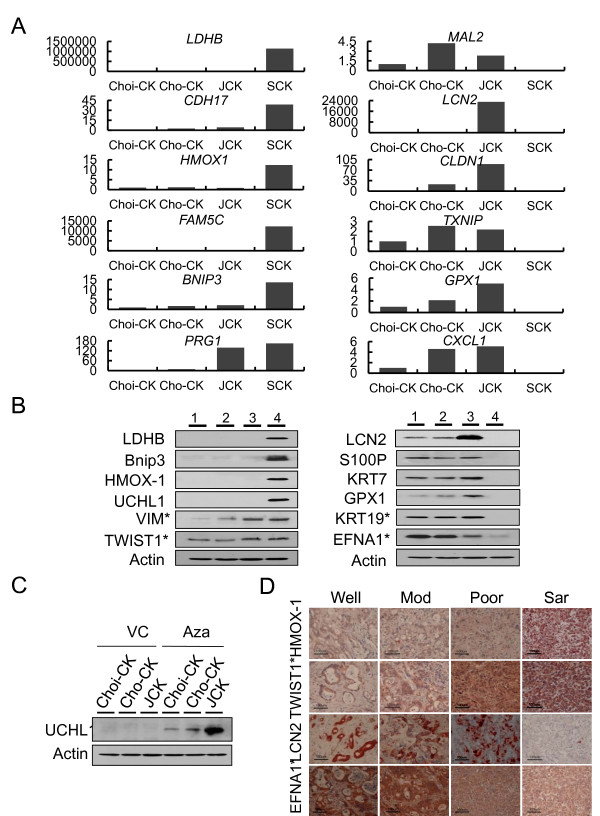
**Genes and proteins differentially expressed in sarcomatoid CC and adenomatous CC cells**. (A) Real-time RT-PCR analysis of upregulated (left) and downregulated (right) genes selected from the list of top 25 genes differentially expressed in sarcomatoid SCK cells and three adenocarcinomatous CC cell lines. (B) Immunoblot of upregulated (left) and down-regulated (right) proteins in sarcomtoid SCK cells compared to three adenocarcinomatous CC cell lines. *, selected from the top 100 genes differentially expressed in sarcomatoid SCK cells compared to three adenocarcinomatous CC cell lines. Lane 1, Choi-CK cells. Lane 2, Cho-CK cells. Lane 3, JCK cells, Lane 4, SCK cells. (C) Immunoblot of UCHL1. Expression was restored in adenocarcimatous CC cells by treatment of 5 μM of Aza for 4 days, compared to vehicle control (VC). (D) Immunohistochemical staining of down- or upregulated proteins according to tumor dedifferentiation. The representative picture is one of three immunohistochemical staining replicates of different specimens. Well, well-differentiated CC. Mod, moderately differentiated CC. Poor, poorly differentiated CC. Sar, sarcomatoid CC. *, selected from the top 100 genes differentially expressed in sarcomatoid SCK cells compared to three adenocarcinomatous CC cell lines.

From 559 genes that were differentially regulated between SCK cells and the three ordinary CC lines, we selected six upregulated genes and six downregulated genes, and examined their mRNA expression using real-time RT-PCR (Figure 6A), which verified the differential expression. We examined protein expression by Western blot analysis of the four CC lines. LDHB, Bnip3, HO-1, and UCHL1 were overexpressed exclusively in SCK cells. The expression of VIM and TWIST1 increased according to tumor dedifferentiation and was highest in SCK cells (Figure 6B, left). In contrast, LCN2, S100P, KRT7, KRT19, GPX1, and EFNA1 were preferentially expressed in Choi-CK, Cho-CK and JCK cells, but minimally expressed in SCK cells (Figure 6B, right). Because *LDHB*, *BNIP3*, and *UCHL1 *are well-known methylation-silenced genes in tumors [39-41], and are highly expressed in SCK cells, this suggested that DNA demethylation was involved in CC. To confirm this hypothesis, we treated the AC cells with the demethylating agent Aza, which dramatically restorated expression of the silenced *UCHL1 *gene in these cells (Figure 6C). In addition, we performed immunohistochemical examination of protein expression according to tumor dedifferentiation in human CC tissue (Figure 6D). As expected, HO-1 was exclusively overexpressed in SC, while TWIST1 was overexpressed in the poorly differentiated and SC cells. In contrast, LCN2 was exclusively downregulated in SC, while EFNA1 expression decreased with tumor dedifferentiation. Therefore, expression of these proteins clearly correlated with clinicopathological features such as tumor differentiation and EMT change, in CC tissues.

## Discussion

In this study, our experimental design primarily investigated the gene expression profiles of 10 cell lines and 19 CC tissues, and compared these profiles to those from four cultured NBE cell line using genome-wide BeadChip microarray analysis. Transdifferentiation-related genes were analyzed by same method. Using unsupervised hierarchical clustering analysis, we found that the *SPP1*, *EFNB2*, and *E2F *genes were commonly upregulated in both cell and tissue samples. *IRX3*, *PTTG1*, and *PPARγ *were upregulated in the cell samples, and were immunohistochemically verified in human and hamster CC tissues. SPP1 (osteopontin), a secretory adhesive alycoprotein, was identified as a highly overexpressed gene in CC lines and tissues. SPP1 is a ligand of CD44 that binds to αV-containing integrins and is important in malignant cell attachment and tumor invasion [43]. It was a highly overexpressed gene in HCC, and its expression correlated with earlier recurrence, poorer prognosis, and metastasis [44]. Consistent with our findings, a recent oligonucleotide microarray study reported that SPP1 was the most highly expressed gene in intrahepatic cholangiocarcinoma [45]. EFNB2 was identified as a preferentially expressed genes in CC. EFNB2 overexpression is reported to be significantly correlated with the number of lymph node metastases and clinical stage in esophageal cancer [46]. Several reports have examined concomitant expression of the ligand EFNB2 and its receptor EphB4 in leukemia-lymphoma cell lines [47], and in endometrial cancer [48]. E2Fs 1-3 are characterized as "activator E2Fs" since their binding to promoters results in increased transcription, while E2Fs 4 and 5 are "repressor E2Fs" since they form complexes with p130, HDACs, and other factors to block transcription [49]. During hepatocarcinogenesis in c-myc/TGFalpha double-transgenic mice, expression of E2F-1 and E2F-2 increases, and putative E2F target genes are induced [50].

For immunohistochemical verification, the representative genes were selected from the list of top 25 commonly upregulated genes, according to antibody available for immunohistochemistry. In addition, other genes were selected from only cell-based microarray database. The same immunohistochemical staining in hamster CC tissues induced by *Clonorchiasis *infestation was compared with control staining in normal hamster livers. IRX3 is involved in dorsal-ventral patterning in spinal cord development and coordination with other homeobox genes [51]. IRX3 is preferentially expressed in the examined CC tissues and localized to the nucleus of human and hamster malignant biliary epithelial cells, independent of cell differentiation. A methylated CpG island was detected in exon 2 of the IRX3 locus, rather than in the promoter, and is responsible for IRX3 overexpression in brain tumor cells and tissues [52]. PTTG1, a critical mitotic checkpoint protein, is a known proto-oncogene that is highly expressed in HCC [53]. Our data showed that PTTG1 was preferentially expressed in the cytoplasm of the human and hamster CC cells. PPAR-*γ*, a member of the nuclear receptor superfamily, functions as a ligand-activated transcription factor [54]. It is overexpressed in a variety of cancers, including HCC and pancreatic cancer [55,56]. Positive immunostaining was localized in the cytoplasm and nuclei of human CC cells. However, positive immunostaining was exclusively detected in the nuclei of the hamster CC cells. Our data also immunohistochemically validated the downregulation of proteins KRT17, UCHL1, IGFBP7, and SPARC. Our hamster model showed the similar expression patterns of human CC related genes and therefore might be a relevant model to study human CC.

Analysis of genes involved in the transdifferentiation of CC cells showed two clusters in the gene axis, with genes that were upregulated (cluster II), and downregulated (cluster I) in the SC group as compared to the AC group. The mesenchymal antigen VIM and the transcriptional factor TWIST1 were upregulated in JCK and SCK cells by tumor dedifferentiation. The overexpression of these proteins is reported to be associated with the EMT [57,58]. Intriguingly, genes silenced by promoter hypermethylation during CC development were restored at the point of sarcomatous transdifferentation, which implied that the demethylation may be involved in the EMT progression of CC.

In addition to tumor-related genes known to be overexprssed in intrahepatic CC, we identified other strongly and consistently dysregulated genes in CC that are known to be involved in other human cancers. Our data support a correlation between the expression of these genes and CC tumor differentiation, and the gene expression patterns found in this study are consistent with those associated with a poor clinical prognosis for this cancer. gene expression profiling appears to be a useful diagnostic tool, especially for differentiating CC from other liver masses, as well as for the subclassification of intrahepatic CC compared to histopathological findings.

## Conclusions

Gene expression profiling appears to be a useful diagnostic tool, especially for differentiating CC from other liver masses, as well as for the subclassification of intrahepatic CC compared to histopathological findings. The most consistently overexpressed genes are candidate therapeutic targets, and related genes can be used for predicting survival and outcomes for different therapeutic modalities.

## Competing interests

The authors declare that they have no competing interests.

## Authors' contributions

MS and IC performed most of the experiments and drafted the manuscript. ML and GY carried out the tissue collection and the establishment of cell lines. XC participated in the immunohistochemical analysis. BC and IK participated in the design and coordination of the study and helped to draft the manuscript. EA and SL participated in the array data processing and analysis. DK conceived of the study, and participated in its design and coordination. All authors read and approved the final manuscript.

## Pre-publication history

The pre-publication history for this paper can be accessed here:

http://www.biomedcentral.com/1471-2407/11/78/prepub

## Supplementary Material

Additional file 1 **Supplementary Table S1:** Sequences and accession numbers for the forward (FOR) and reverse (REV) primers used in real-time RT-PCR. Click here for file

Additional file2 **Supplementary Table S2**: List of genes differentially expressed between differentiated cholangiocarcinoma and normal biliary epithelium (NBE). Click here for file

Additional file 3 **Supplementary Table S3**: List of genes differentially expressed between undifferentiated cholangiocarcinoma and normal biliary epithelium (NBE). Click here for file

Additional file 4 **Supplementary Table S4**: Top 25 genes commonly regulated in both CC cells and tissues compared with cultured biliary epithelial cells. Click here for file

Additional file 5 **Supplementary Figure S1**: Immunohistochemical staining with anti-SPP1, anti-EFNB2, anti-E2F2, and anti-IRX3 in hamster CC tissues induced by Clonorchiasis infestation. Control stainings were performed in normal hamster livers. *, selected from only cell-based microarray database. 35 Click here for file

Additional file 6 **Supplementary Table 5**. Top 25 genes differentially expressed in the sarcomatoid SCK cells compared with 3 adenocarcinomatous CC cell lines. Click here for file
