# Supplementary material for: Genome-wide expression patterns associated with oncogenesis and sarcomatous transdifferentation of cholangiocarcinoma
Source: BMC Cancer. 2011 Feb 19;11:78. doi: 10.1186/1471-2407-11-78 (PMC3053267; doi:10.1186/1471-2407-11-78)
Supplement: Additional file 1 — Supplementary Table S1: Sequences and accession numbers for the forward (FOR) and reverse (REV) primers used in real-time RT-PCR. [file 1471-2407-11-78-S1.DOCX]

**Supplementary Table 1: Sequences and accession numbers for the forward (FOR) and reverse (REV) primers used in real-time RT-PCR.**

| **Gene** | **Sequences for primers** | **Acession No.** |
| --- | --- | --- |
| SPP1 | FOR : GTGATTTGCTTTTGCCTCCT  REV : GCCACAGCATCTGGGTATTT | NM_000582 |
| PALC8 | FOR : CTGTCTGTGTGGAACAAGCG  REV : GAGGACAGCAAAGAGTTGCC | NM_016691 |
| EFNB2 | FOR : TGGGGTGTTTTGATGGTTTT  REV : ACCAGTCCTTGTCCAGGTAGAA | NM_004093 |
| ZIC2 | FOR : AAGATCCACAAAAGGACCCA  REV : TGCATGTGCTTCTTCCTGTC | NM_007129 |
| E2F2 | FOR : CAAGTTGTGCGATGCCTG  REV : TTGGGAACTCAGGGACGA | NM_004091 |
| IRX3 | FOR : AGTGCCTTGGAAGTGGAGAA  REV : GGAGAGAGCCGATAAGACCA | NM_024336 |
| PTTG1 | FOR : GCCTCAGATGATGCCTATCC  REV : ATCTGGTGCTCTTCAGGCAG | NM_004219 |
| PPARγ | FOR : AAGGCCATTTTCTCAAACGA  REV : GAGAGATCCACGGAGCTGAT | NM_005037 |
| COL1A2 | FOR : GAAAAGGAGTTGGACTTGGC  REV : AGCAGGTCCTTGGAAACCTT | NM_000089 |
| SULF1 | FOR : ATGCAGGTTCTTCAAGGCAG  REV : ATCCTGGTTGAATAATCAATCTCT | NM_015170 |
| UCHL1 | FOR : AACTTGATGGACGAATGCCT  REV : AATTCTCTGCAGACCTTGGC | NM_004181 |
| CDH11 | FOR : GTTGCGTCCACCCTCAAG  REV : TTGGTCACTCAACAAATGACAA | NM_001797 |
| IGFBP7 | FOR : CATCACCCAGGTCAGCAAG  REV : GCTCAAGTACACCTGGGCAC | NM_002178 |
| SPARC | FOR : GCCTGTCTCTAAACCCCTCC  REV : GAGAAAGAAGATCCAGGCCC | NM_003118 |

| LDHB | FOR : GGTATGGCGTGTGCTATCAG  REV : TGCTGCAGATCCATCATTTC | NM_002300 |
| --- | --- | --- |
| CDH17 | FOR : TTTACATTTTCCCTCGGCAG  REV : CCTCAAACTCTGTGTGCCTG | NM_004063 |
| HMOX1 | FOR : GCCAGCAACAAAGTGCAAG  REV : GAGTGTAAGGACCCATCGGA | NM_002133 |
| FAM5C | FOR : CCATCGCTCACAGGAATACA  REV : TATTTACTTTCCAGCGGCCA | NM_199051 |
| BNIP3 | FOR : GATGCAGGAGGAGAGCCTG  REV : AATAGAAACCGAGGCTGGAA | NM_004052 |
| PRG1 | FOR : TCCTGGTTCTGGAATCCTCA  REV : TTCTTCAAGGCAGTTTGCAG | NM_002727 |
| MAL2 | FOR : ATGTTCCTCTCTGGCATGGT  REV : GCTTCCAATAAAAAGGCTCC | NM_052886 |
| LCN2 | FOR : ATGTCACCTCCGTCCTGTTT  REV : ACTCTTAATGTTGCCCAGCG | NM_005564 |
| CLDN1 | FOR : GCAGATCCAGTGCAAAGTCT  REV : CATACACTTCATGCCAACGG | NM_021101 |
| TXNIP | FOR : ACGCTTCTTCTGGAAGACCA  REV : AGGAAGCTCAAAGCCGAACT | NM_006472 |
| GPX1 | FOR : ACCTACGAGGGAGGAACACC  REV : ATTAGTGGGGAAACTCGCCT | NM_201397 |
| CSCL1 | FOR : GAAAGCTTGCCTCAATCCTG  REV : CTTCCTCCTCCCTTCTGGTC | NM_001511 |
