# Supplementary material for: Genome-wide expression patterns associated with oncogenesis and sarcomatous transdifferentation of cholangiocarcinoma
Source: BMC Cancer. 2011 Feb 19;11:78. doi: 10.1186/1471-2407-11-78 (PMC3053267; doi:10.1186/1471-2407-11-78)
Supplement: Additional file2 — Supplementary Table S2: List of genes differentially expressed between differentiated cholangiocarcinoma and normal biliary epithelium (NBE). [file 1471-2407-11-78-S2.DOC]

Supplementary Table 2: List of genes differentially expressed between differntiated cholangiocarcinoma and normal biliary epithelium (NBE).

| **Rank** | **Accession No.** | **Title** | **Symbol** | **Mean fold change** | ***P* Value** | ***q*-Value** |
| --- | --- | --- | --- | --- | --- | --- |
| 1 | NM_003226.2 | Trefoil factor 3 | *TFF3* | 107.7 | 5.09E-05 | 0.0036166 |
| 2 | NM_031310.1 | Plasmalemma vesicle associated protein | *PLVAP* | 51.2 | 1.32E-08 | 7.67E-05 |
| 3 | NM_000239.1 | Lysozyme | *LYZ* | 48.0 | 3.09E-06 | 0.0008631 |
| 4 | NM_000014.4 | Alpha-2-macroglobulin | *A2M* | 43.7 | 4.22E-08 | 0.0001272 |
| 5 | NM_002909.3 | Regenerating islet-derived 1 alpha | *REG1A* | 40.5 | 0.0026752 | 0.0282643 |
| 6 | NM_002644.2 | Polymeric immunoglobulin receptor | *PIGR* | 38.9 | 8.33E-05 | 0.0045209 |
| 7 | NM_000582.2 | Secreted phosphoprotein 1 | *SPP1* | 38.3 | 7.22E-05 | 0.004169 |
| 8 | NM_000518.4 | Hemoglobin, beta | *HBB* | 36.6 | 0.0003096 | 0.0090942 |
| 9 | NM_019111.3 | Major histocompatibility complex, class II, DR alpha | *HLA-DRA* | 32.4 | 0.0005339 | 0.0118713 |
| 10 | NM_000477.3 | Albumin | *ALB* | 32.1 | 0.0005077 | 0.0115122 |
| 11 | NM_004063.2 | Cadherin 17, LI cadherin | *CDH17* | 32.0 | 0.0010892 | 0.0176053 |
| 12 | NM_003332.2 | TYRO protein tyrosine kinase binding protein , transcript variant 1 | *TYROBP* | 29.4 | 1.34E-06 | 0.0006013 |
| 13 | NM_015991.1 | Complement component 1, q subcomponent, alpha polypeptide | *C1QA* | 27.0 | 1.83E-06 | 0.0006631 |
| 14 | XM_941953.1 | PREDICTED: similar to Ig kappa chain V-I region HK102 precursor | *LOC652493* | 25.9 | 7.49E-05 | 0.0042504 |
| 15 | NM_001629.2 | Arachidonate 5-lipoxygenase-activating protein | *ALOX5AP* | 22.4 | 2.80E-07 | 0.0004472 |
| 16 | NM_005518.2 | 3-hydroxy-3-methylglutaryl-Coenzyme A synthase 2 | *HMGCS2* | 22.1 | 0.0004254 | 0.0108127 |
| 17 | NM_002964.3 | S100 calcium binding protein A8 | *S100A8* | 21.1 | 6.88E-06 | 0.0013229 |
| 18 | XM_942302.1 | PREDICTED: similar to Ig kappa chain V-I region HK102 precursor | *LOC652694* | 20.5 | 0.0001721 | 0.0067823 |
| 19 | NM_002153.1 | Hydroxysteroid dehydrogenase 2 | *HSD17B2* | 20.4 | 1.36E-06 | 0.0006013 |
| 20 | NM_000035.2 | Aldolase B, fructose-bisphosphate | *ALDOB* | 20.2 | 0.0001992 | 0.0073217 |
| 21 | NM_000295.3 | Serpin peptidase inhibitor, clade A , member 1 , transcript variant 1 | *SERPINA1* | 20.1 | 0.0013744 | 0.0198888 |
| 22 | NM_000667.2 | Alcohol dehydrogenase 1A , alpha polypeptide | *ADH1A* | 19.6 | 0.0026784 | 0.0282814 |
| 23 | NM_000777.2 | Cytochrome P450, family 3, subfamily A, polypeptide 5 | *CYP3A5* | 19.5 | 3.00E-06 | 0.0008489 |
| 24 | NM_003122.2 | Serine peptidase inhibitor, Kazal type 1 | *SPINK1* | 19.1 | 0.0014657 | 0.0206436 |
| 25 | NM_012072.2 | Complement component 1, q subcomponent, receptor 1 | *C1QR1* | 19.0 | 7.20E-08 | 0.000178 |
| 26 | NM_000039.1 | Apolipoprotein A-I | *APOA1* | 18.9 | 0.0005854 | 0.0125602 |
| 27 | NM_000552.2 | Von Willebrand factor | *VWF* | 18.8 | 4.43E-07 | 0.0004727 |
| 28 | NM_000558.3 | Hemoglobin, alpha 1 | *HBA1* | 18.7 | 0.0006906 | 0.0137823 |
| 29 | NM_176813.3 | Breast cancer membrane protein 11 | *BCMP11* | 18.6 | 0.0012071 | 0.0186228 |
| 30 | NM_002922.3 | Regulator of G-protein signalling 1 | *RGS1* | 18.5 | 1.99E-05 | 0.0024002 |
| 31 | XM_936253.1 | PREDICTED: similar to Ig kappa chain V-III region HAH precursor | *LOC642113* | 18.5 | 6.62E-05 | 0.0040261 |
| 32 | NM_000583.2 | Group-specific component | *GC* | 18.4 | 0.0002561 | 0.0083025 |
| 33 | NM_006762.1 | Lysosomal associated multispanning membrane protein 5 | *LAPTM5* | 17.3 | 1.39E-06 | 0.0006013 |
| 34 | NM_000491.2 | Complement component 1, q subcomponent, beta polypeptide | *C1QB* | 16.8 | 3.47E-06 | 0.0009415 |
| 35 | NM_005211.2 | Colony stimulating factor 1 receptor, formerly mcdonough feline sarcoma viral oncogene homolog | *CSF1R* | 16.7 | 2.92E-06 | 0.0008459 |
| 36 | NM_005410.2 | Selenoprotein P, plasma, 1 | *SEPP1* | 16.5 | 2.94E-05 | 0.0028148 |
| 37 | NM_000567.2 | C-reactive protein, pentraxin-related | *CRP* | 16.3 | 0.0070056 | 0.049033 |
| 38 | NM_002083.2 | Glutathione peroxidase 2 | *GPX2* | 16.1 | 0.0001424 | 0.0060544 |
| 39 | NM_000211.1 | Integrin, beta 2 , lymphocyte function-associated antigen 1; macrophage antigen 1 beta subunit) | *ITGB2* | 16.1 | 0.0005706 | 0.0123813 |
| 40 | NM_173596.1 | Solute carrier family 39 , member 5 | *SLC39A5* | 15.9 | 0.0002752 | 0.0086092 |
| 41 | NM_005141.2 | Fibrinogen beta chain | *FGB* | 15.9 | 0.0035749 | 0.0334441 |
| 42 | NM_000067.1 | Carbonic anhydrase II | *CA2* | 15.9 | 7.76E-05 | 0.0043597 |
| 43 | NM_001633.2 | Alpha-1-microglobulin/bikunin precursor | *AMBP* | 15.8 | 0.0011787 | 0.0183401 |
| 44 | NM_021983.4 | Major histocompatibility complex, class II, DR beta 4 | *HLA-DRB4* | 15.4 | 3.00E-05 | 0.0028431 |
| 45 | NM_002474.1 | Myosin, heavy polypeptide 11, smooth muscle , transcript variant SM1 | *MYH11* | 15.3 | 0.0002301 | 0.0078409 |
| 46 | XM_939003.1 | PREDICTED: similar to Ig gamma-2 chain C region | *LOC649923* | 15.1 | 0.0001175 | 0.0054731 |
| 47 | NM_000597.2 | Insulin-like growth factor binding protein 2, 36kda | *IGFBP2* | 15.1 | 0.00052 | 0.0116937 |
| 48 | NM_006149.2 | Lectin, galactoside-binding, soluble, 4 | *LGALS4* | 15.1 | 0.0003694 | 0.0099631 |
| 49 | NM_001004430.1 | Mucin 17 | *MUC17* | 15.0 | 0.0083789 | 0.054313 |
| 50 | NM_003225.2 | Trefoil factor 1 | *TFF1* | 14.9 | 0.0004004 | 0.0104707 |
| 51 | NM_002118.3 | Major histocompatibility complex, class II, DM beta | *HLA-DMB* | 14.7 | 0.0001319 | 0.0058189 |
| 52 | NM_001645.3 | Apolipoprotein C-I | *APOC1* | 14.4 | 0.0001515 | 0.0063107 |
| 53 | XM_936518.1 | PREDICTED: similar to Ig kappa chain V-I region HK101 precursor | *LOC647450* | 14.3 | 0.0002036 | 0.0073714 |
| 54 | NM_172369.2 | Complement component 1, q subcomponent, C chain | *C1QC* | 14.1 | 1.10E-05 | 0.0017058 |
| 55 | NM_002298.2 | Lymphocyte cytosolic protein 1 , transcript variant 5 | *LCP1* | 14.1 | 1.98E-08 | 7.67E-05 |
| 56 | NM_001443.1 | Fatty acid binding protein 1, liver | *FABP1* | 13.9 | 0.0007116 | 0.013955 |
| 57 | NM_001025158.1 | CD74 antigen , transcript variant 3 | *CD74* | 13.7 | 0.0011081 | 0.0177624 |
| 58 | NM_033049.2 | Mucin 13, epithelial transmembrane | *MUC13* | 13.7 | 0.0022227 | 0.0254996 |
| 59 | NM_000343.1 | Solute carrier family 5 , member 1 | *SLC5A1* | 13.6 | 3.91E-05 | 0.0031756 |
| 60 | NM_004684.2 | SPARC-like 1 | *SPARCL1* | 13.3 | 1.06E-05 | 0.0016716 |
| 61 | NM_000790.2 | Dopa decarboxylase | *DDC* | 13.3 | 0.0012466 | 0.018937 |
| 62 | NM_000669.3 | Alcohol dehydrogenase 1C , gamma polypeptide | *ADH1C* | 13.2 | 0.0020681 | 0.0244798 |
| 63 | NM_000385.3 | Aquaporin 1 , transcript variant 2 | *AQP1* | 13.1 | 1.89E-05 | 0.0023784 |
| 64 | NM_004497.2 | Forkhead box A3 | *FOXA3* | 13.0 | 6.35E-06 | 0.0013036 |
| 65 | NM_002980.1 | Secretin receptor | *SCTR* | 13.0 | 0.003833 | 0.0347948 |
| 66 | NM_001216.1 | Carbonic anhydrase IX | *CA9* | 12.8 | 0.0005762 | 0.0124218 |
| 67 | NM_017899.1 | Tescalcin | *TESC* | 12.7 | 0.0061156 | 0.0453545 |
| 68 | NM_001012993.1 | Chromosome 9 open reading frame 152 | *C9ORF152* | 12.6 | 0.0004474 | 0.0109221 |
| 69 | NM_020796.2 | Sema domain, transmembrane domain , and cytoplasmic domain, 6A | *SEMA6A* | 12.4 | 1.80E-06 | 0.0006631 |
| 70 | NM_004363.1 | Carcinoembryonic antigen-related cell adhesion molecule 5 | *CEACAM5* | 12.2 | 0.0066345 | 0.0474393 |
| 71 | NM_000371.1 | Transthyretin | *TTR* | 12.2 | 0.0003029 | 0.0090067 |
| 72 | NM_020384.2 | Claudin 2 | *CLDN2* | 11.9 | 0.0009378 | 0.0161183 |
| 73 | XM_936120.1 | PREDICTED: major histocompatibility complex, class II, DQ alpha 1, transcript variant 2 | *HLA-DQA1* | 11.8 | 6.09E-05 | 0.0039049 |
| 74 | NM_015234.3 | G protein-coupled receptor 116 | *GPR116* | 11.7 | 4.63E-06 | 0.001101 |
| 75 | NM_018419.2 | SRY -box 18 | *SOX18* | 11.7 | 8.07E-07 | 0.0005331 |
| 76 | NM_001175.4 | Rho GDP dissociation inhibitor beta | *ARHGDIB* | 11.4 | 0.0014086 | 0.0202264 |
| 77 | NM_000508.3 | Fibrinogen alpha chain , transcript variant alpha-E | *FGA* | 11.4 | 0.0058885 | 0.0445135 |
| 78 | NM_005143.2 | Haptoglobin | *HP* | 11.3 | 0.0088957 | 0.0558635 |
| 79 | NM_000591.1 | CD14 antigen | *CD14* | 11.1 | 1.75E-06 | 0.0006631 |
| 80 | NM_032782.3 | Hepatitis A virus cellular receptor 2 | *HAVCR2* | 11.1 | 3.35E-06 | 0.0009177 |
| 81 | NM_022349.2 | Membrane-spanning 4-domains, subfamily A, member 6A , transcript variant 2 | *MS4A6A* | 11.1 | 2.83E-06 | 0.0008457 |
| 82 | NM_033554.2 | Major histocompatibility complex, class II, DP alpha 1 | *HLA-DPA1* | 10.9 | 0.0011553 | 0.018111 |
| 83 | NM_006418.3 | Olfactomedin 4 | *OLFM4* | 10.6 | 0.0082904 | 0.0539843 |
| 84 | NM_012067.2 | Aldo-keto reductase family 7, member A3 | *AKR7A3* | 10.5 | 2.13E-05 | 0.0024707 |
| 85 | XM_939969.1 | PREDICTED: similar to HSPC323 | *LOC284422* | 10.4 | 0.0001771 | 0.0068827 |
| 86 | NM_005565.3 | Lymphocyte cytosolic protein 2 | *LCP2* | 10.4 | 7.00E-10 | 1.90E-05 |
| 87 | NM_001091.2 | Amiloride binding protein 1 ) | *ABP1* | 10.3 | 0.0012661 | 0.0190866 |
| 88 | NM_004364.2 | CCAAT/enhancer binding protein , alpha | *CEBPA* | 10.3 | 1.58E-06 | 0.0006376 |
| 89 | NM_016619.1 | Placenta-specific 8 | *PLAC8* | 10.0 | 0.0005937 | 0.012619 |
| 90 | NM_001063.2 | Transferrin | *TF* | 10.0 | 0.0018292 | 0.0231388 |
| 91 | NM_014571.2 | Hairy/enhancer-of-split related with YRPW motif-like | *HEYL* | 10.0 | 0.0005168 | 0.0116514 |
| 92 | NM_005849.1 | Immunoglobulin superfamily, member 6 | *IGSF6* | 9.9 | 2.26E-06 | 0.000775 |
| 93 | NM_001482.1 | Glycine amidinotransferase | *GATM* | 9.9 | 0.0017714 | 0.0227902 |
| 94 | NM_024889.3 | Chromosome 10 open reading frame 81 | *C10ORF81* | 9.7 | 0.0001569 | 0.0064512 |
| 95 | NM_005379.2 | Myosin IA | *MYO1A* | 9.7 | 0.0014389 | 0.020436 |
| 96 | NM_000384.1 | Apolipoprotein B antigen) | *APOB* | 9.7 | 0.0007545 | 0.01439 |
| 97 | NM_018326.2 | Gtpase, IMAP family member 4 | *GIMAP4* | 9.4 | 9.82E-07 | 0.0005664 |
| 98 | NM_002928.2 | Regulator of G-protein signalling 16 | *RGS16* | 9.3 | 5.02E-06 | 0.0011448 |
| 99 | NM_000587.2 | Complement component 7 | *C7* | 9.3 | 0.0014426 | 0.0204421 |
| 100 | NM_022555.3 | Major histocompatibility complex, class II, DR beta 3 | *HLA-DRB3* | 9.2 | 2.19E-05 | 0.0024935 |
| 101 | NM_006408.2 | Anterior gradient 2 homolog | *AGR2* | 9.2 | 0.0005606 | 0.012262 |
| 102 | NM_006120.2 | Major histocompatibility complex, class II, DM alpha | *HLA-DMA* | 9.0 | 0.0001018 | 0.0050215 |
| 103 | NM_005940.3 | Matrix metallopeptidase 11 | *MMP11* | 8.9 | 0.0011481 | 0.0180976 |
| 104 | NM_000142.2 | Fibroblast growth factor receptor 3 , transcript variant 1 | *FGFR3* | 8.6 | 9.67E-05 | 0.0048583 |
| 105 | XM_940969.1 | PREDICTED: similar to Ig kappa chain V-II region RPMI 6410 precursor | *LOC651751* | 8.6 | 0.0006242 | 0.0129436 |
| 106 | NM_001643.1 | Apolipoprotein A-II | *APOA2* | 8.5 | 0.0069081 | 0.0486044 |
| 107 | NM_005170.2 | Achaete-scute complex-like 2 | *ASCL2* | 8.4 | 0.007498 | 0.0509787 |
| 108 | NM_018487.2 | Hepatocellular carcinoma-associated antigen 112 | *HCA112* | 8.2 | 0.0010418 | 0.0172024 |
| 109 | NM_024795.1 | Transmembrane 4 L six family member 20 | *TM4SF20* | 8.2 | 0.0021789 | 0.0252217 |
| 110 | NM_033229.1 | Tripartite motif-containing 15 , transcript variant 1 | *TRIM15* | 8.2 | 4.11E-05 | 0.0032584 |
| 111 | NM_004877.1 | Glia maturation factor, gamma | *GMFG* | 8.2 | 5.02E-07 | 0.0005046 |
| 112 | NM_002163.2 | Interferon regulatory factor 8 | *IRF8* | 8.1 | 6.31E-05 | 0.0039881 |
| 113 | NM_206939.1 | Membrane-spanning 4-domains, subfamily A, member 7 , transcript variant 3 | *MS4A7* | 8.0 | 6.01E-05 | 0.0039049 |
| 114 | NM_000784.2 | Cytochrome P450, family 27, subfamily A, polypeptide 1 , nuclear gene encoding mitochondrial protein | *CYP27A1* | 7.9 | 2.57E-06 | 0.0008237 |
| 115 | NM_002664.1 | Pleckstrin | *PLEK* | 7.9 | 1.80E-08 | 7.67E-05 |
| 116 | XM_944822.1 | PREDICTED: similar to HLA class II histocompatibility antigen, DRB1-9 beta chain precursor , transcript variant 2 | *LOC649143* | 7.7 | 5.78E-05 | 0.0038231 |
| 117 | NM_004120.3 | Guanylate binding protein 2, interferon-inducible | *GBP2* | 7.7 | 1.04E-05 | 0.0016659 |
| 118 | NM_004004.3 | Gap junction protein, beta 2, 26kda | *GJB2* | 7.6 | 6.56E-05 | 0.0040208 |
| 119 | NM_000773.3 | Cytochrome P450, family 2, subfamily E, polypeptide 1 | *CYP2E1* | 7.6 | 0.0074229 | 0.0507223 |
| 120 | NM_003196.1 | Transcription elongation factor A , 3 | *TCEA3* | 7.6 | 0.0002998 | 0.0089553 |
| 121 | NM_000341.2 | Solute carrier family 3 , member 1 | *SLC3A1* | 7.5 | 0.0006417 | 0.0131952 |
| 122 | NM_001145.2 | Angiogenin, ribonuclease, rnase A family, 5 | *ANG* | 7.5 | 2.03E-05 | 0.0024002 |
| 123 | NM_174896.2 | Chromosome 1 open reading frame 162 | *C1ORF162* | 7.5 | 3.31E-05 | 0.0029394 |
| 124 | NM_001463.2 | Frizzled-related protein | *FRZB* | 7.3 | 1.07E-06 | 0.0005945 |
| 125 | NM_021175.2 | Hepcidin antimicrobial peptide | *HAMP* | 7.2 | 0.0025776 | 0.0277607 |
| 126 | NM_004106.1 | Fc fragment of ige, high affinity I, receptor for; gamma polypeptide | *FCER1G* | 7.2 | 5.18E-05 | 0.0036597 |
| 127 | NM_024320.2 | Atpase family, AAA domain containing 4 | *ATAD4* | 7.2 | 4.86E-05 | 0.003528 |
| 128 | NM_001150.1 | Alanyl aminopeptidase | *ANPEP* | 7.2 | 0.0054114 | 0.0423382 |
| 129 | NM_020980.2 | Aquaporin 9 | *AQP9* | 7.1 | 2.51E-05 | 0.0026584 |
| 130 | NM_005241.1 | Ecotropic viral integration site 1 | *EVI1* | 7.1 | 0.0006133 | 0.0128451 |
| 131 | NM_022138.1 | SPARC related modular calcium binding 2 | *SMOC2* | 7.1 | 6.93E-05 | 0.0040925 |
| 132 | NM_000715.3 | Complement component 4 binding protein, alpha | *C4BPA* | 6.9 | 0.0014366 | 0.020436 |
| 133 | NM_032044.2 | Regenerating islet-derived family, member 4 | *REG4* | 6.8 | 0.0080019 | 0.0528806 |
| 134 | NM_000507.2 | Fructose-1,6-bisphosphatase 1 | *FBP1* | 6.8 | 9.91E-05 | 0.0049501 |
| 135 | NM_001031733.1 | Calmodulin-like 4 , transcript variant 1 | *CALML4* | 6.8 | 1.22E-05 | 0.0018546 |
| 136 | NM_005630.1 | Solute carrier organic anion transporter family, member 2A1 | *SLCO2A1* | 6.8 | 0.0012258 | 0.0187136 |
| 137 | BG545303 | 602572519F1 NIH_MGC_77 cdna clone IMAGE:4700548 5, mrna sequence | *HS.137274* | 6.6 | 0.006857 | 0.0483415 |
| 138 | NM_002029.3 | Formyl peptide receptor 1 | *FPR1* | 6.6 | 4.19E-07 | 0.0004727 |
| 139 | NM_001007595.1 | Nuclear localized factor 2 | *NLF2* | 6.5 | 0.0011973 | 0.0185348 |
| 140 | NM_003810.2 | Tumor necrosis factor superfamily, member 10 | *TNFSF10* | 6.5 | 0.0024508 | 0.0269916 |
| 141 | NM_152311.1 | Transmembrane protein 12 | *TMEM12* | 6.5 | 4.84E-05 | 0.003528 |
| 142 | NM_001666.2 | Rho gtpase activating protein 4 | *ARHGAP4* | 6.5 | 4.33E-06 | 0.0010797 |
| 143 | NM_000494.2 | Collagen, type XVII, alpha 1 , transcript variant long | *COL17A1* | 6.3 | 0.0053616 | 0.0420878 |
| 144 | NM_002885.1 | RAP1, gtpase activating protein 1 | *RAP1GA1* | 6.3 | 0.0009469 | 0.0161828 |
| 145 | NM_005161.2 | Angiotensin II receptor-like 1 | *AGTRL1* | 6.3 | 3.91E-05 | 0.0031756 |
| 146 | NM_004591.1 | Chemokine ligand 20 | *CCL20* | 6.3 | 0.0039133 | 0.0352603 |
| 147 | NM_001074.1 | UDP glucuronosyltransferase 2 family, polypeptide B7 | *UGT2B7* | 6.3 | 0.0013473 | 0.0196959 |
| 148 | XM_939056.1 | PREDICTED: similar to creatine kinase, mitochondrial 1B precursor | *LOC649970* | 6.2 | 0.0008331 | 0.0150193 |
| 149 | NM_031457.1 | Membrane-spanning 4-domains, subfamily A, member 8B | *MS4A8B* | 6.2 | 0.0021863 | 0.0252396 |
| 150 | NM_000377.1 | Wiskott-Aldrich syndrome | *WAS* | 6.2 | 7.86E-07 | 0.000533 |
| 151 | NM_138788.2 | Transmembrane protein 45B | *TMEM45B* | 6.1 | 0.000269 | 0.0085235 |
| 152 | NM_004847.2 | Allograft inflammatory factor 1 , transcript variant 2 | *AIF1* | 6.1 | 2.60E-06 | 0.0008237 |
| 153 | NM_003641.2 | Interferon induced transmembrane protein 1 | *IFITM1* | 6.1 | 0.0021817 | 0.0252234 |
| 154 | NM_000856.2 | Guanylate cyclase 1, soluble, alpha 3 | *GUCY1A3* | 6.1 | 0.0008254 | 0.0149945 |
| 155 | NM_001311.3 | Cysteine-rich protein 1 | *CRIP1* | 6.1 | 0.0025309 | 0.0275289 |
| 156 | NM_001185.2 | Alpha-2-glycoprotein 1, zinc | *AZGP1* | 6.1 | 4.27E-05 | 0.0033117 |
| 157 | X00437 | Human mrna for T-cell specific protein | *HS.534427* | 6.0 | 1.37E-06 | 0.0006013 |
| 158 | NM_019062.1 | Ring finger protein 186 | *RNF186* | 6.0 | 0.0065195 | 0.0471258 |
| 159 | NM_001066.2 | Tumor necrosis factor receptor superfamily, member 1B | *TNFRSF1B* | 6.0 | 2.35E-05 | 0.0025994 |
| 160 | NM_000493.2 | Collagen, type X, alpha 1 | *COL10A1* | 6.0 | 0.0047442 | 0.0392278 |
| 161 | NM_001979.4 | Epoxide hydrolase 2, cytoplasmic | *EPHX2* | 5.9 | 0.0002569 | 0.0083148 |
| 162 | NM_007256.2 | Solute carrier organic anion transporter family, member 2B1 | *SLCO2B1* | 5.9 | 1.32E-07 | 0.0002974 |
| 163 | NM_013261.2 | Peroxisome proliferative activated receptor, gamma, coactivator 1, alpha | *PPARGC1A* | 5.9 | 1.81E-05 | 0.0023355 |
| 164 | NM_000574.2 | CD55 antigen, decay accelerating factor for complement | *CD55* | 5.8 | 0.0044161 | 0.0378339 |
| 165 | XM_937586.1 | PREDICTED: hypothetical protein FLJ21438 | *FLJ21438* | 5.7 | 5.87E-06 | 0.0012344 |
| 166 | NM_153338.1 | Gamma-glutamyltransferase 6 homolog | *GGT6* | 5.7 | 8.20E-05 | 0.0044757 |
| 167 | NM_002304.1 | Lunatic fringe homolog | *LFNG* | 5.7 | 0.0025782 | 0.0277607 |
| 168 | NM_014033.2 | Methyltransferase like 7A | *METTL7A* | 5.7 | 0.006868 | 0.0484068 |
| 169 | NM_024027.3 | Collectin sub-family member 11 , transcript variant 1 | *COLEC11* | 5.7 | 0.0027301 | 0.0285555 |
| 170 | BC038512 | Cdna clone IMAGE:5262734 | *HS.296031* | 5.6 | 6.90E-05 | 0.0040925 |
| 171 | NM_002155.3 | Heat shock 70kda protein 6 | *HSPA6* | 5.6 | 2.06E-05 | 0.0024231 |
| 172 | NM_145740.2 | Glutathione S-transferase A1 | *GSTA1* | 5.6 | 0.0027975 | 0.0289007 |
| 173 | NM_145202.3 | Proline-rich acidic protein 1 | *PRAP1* | 5.6 | 0.0028748 | 0.0294001 |
| 174 | NM_002983.1 | Chemokine ligand 3 | *CCL3* | 5.6 | 0.0036207 | 0.0336752 |
| 175 | NM_173561.1 | Unc-5 homolog C -like | *UNC5CL* | 5.5 | 1.55E-05 | 0.0021017 |
| 176 | NM_003621.1 | PTPRF interacting protein, binding protein 2 | *PPFIBP2* | 5.5 | 3.99E-06 | 0.0010164 |
| 177 | NM_153206.1 | Adhesion molecule, interacts with CXADR antigen 1 | *AMICA1* | 5.5 | 2.37E-07 | 0.0004287 |
| 178 | CR627122 | Mrna; cdna dkfzp779m2422 | *HS.291319* | 5.5 | 5.50E-06 | 0.0012228 |
| 179 | NM_025047.1 | ADP-ribosylation factor-like 14 | *ARL14* | 5.5 | 0.0012632 | 0.0190542 |
| 180 | NM_002432.1 | Myeloid cell nuclear differentiation antigen | *MNDA* | 5.5 | 1.03E-06 | 0.0005845 |
| 181 | NM_199327.1 | Sprouty homolog 1, antagonist of FGF signaling , transcript variant 2 | *SPRY1* | 5.5 | 0.0004505 | 0.0109868 |
| 182 | NM_194431.1 | Ribonuclease, rnase A family, 4 , transcript variant 3 | *RNASE4* | 5.4 | 2.92E-05 | 0.0028142 |
| 183 | NM_025243.2 | Solute carrier family 19, member 3 | *SLC19A3* | 5.4 | 0.0014101 | 0.0202264 |
| 184 | NM_006113.3 | Vav 3 oncogene | *VAV3* | 5.4 | 2.42E-06 | 0.0008005 |
| 185 | NM_001001437.2 | Chemokine ligand 3-like 3 | *CCL3L3* | 5.4 | 0.003619 | 0.0336752 |
| 186 | NM_000958.2 | Prostaglandin E receptor 4 | *PTGER4* | 5.4 | 7.39E-05 | 0.0042199 |
| 187 | NM_006209.2 | Ectonucleotide pyrophosphatase/phosphodiesterase 2 | *ENPP2* | 5.4 | 0.0035476 | 0.0332695 |
| 188 | NM_006748.1 | Src-like-adaptor | *SLA* | 5.3 | 4.53E-07 | 0.0004727 |
| 189 | NM_003105.3 | Sortilin-related receptor, L A repeats-containing | *SORL1* | 5.3 | 0.000254 | 0.0082508 |
| 190 | NM_014694.2 | ADAMTS-like 2 | *ADAMTSL2* | 5.3 | 0.0015527 | 0.0212722 |
| 191 | NM_007028.3 | Tripartite motif-containing 31 , transcript variant 1 | *TRIM31* | 5.3 | 0.0036128 | 0.03366 |
| 192 | NM_003467.2 | Chemokine receptor 4 , transcript variant 2 | *CXCR4* | 5.3 | 0.0003915 | 0.0103284 |
| 193 | NM_000300.2 | Phospholipase A2, group IIA | *PLA2G2A* | 5.2 | 0.0088454 | 0.0557739 |
| 194 | NM_173452.1 | Ficolin 3 , transcript variant 2 | *FCN3* | 5.2 | 0.0039183 | 0.0352933 |
| 195 | NM_006096.2 | N-myc downstream regulated gene 1 | *NDRG1* | 5.2 | 0.0013368 | 0.0196084 |
| 196 | NM_024743.2 | UDP glucuronosyltransferase 2 family, polypeptide A3 | *UGT2A3* | 5.2 | 0.0023482 | 0.0262887 |
| 197 | NM_001073.1 | UDP glucuronosyltransferase 2 family, polypeptide B11 | *UGT2B11* | 5.1 | 0.0007607 | 0.0144178 |
| 198 | NM_004093.2 | Ephrin-B2 | *EFNB2* | 5.1 | 0.0007387 | 0.0142293 |
| 199 | XM_937295.1 | PREDICTED: arachidonate 5-lipoxygenase | *ALOX5* | 5.1 | 0.0018139 | 0.023042 |
| 200 | NM_001033047.1 | Nephronectin | *NPNT* | 5.1 | 0.0007078 | 0.0139504 |
| 201 | NM_001010919.1 | Hypothetical protein LOC441168 | *LOC441168* | 5.1 | 7.66E-05 | 0.0043308 |
| 202 | NM_024829.4 | Hypothetical protein FLJ22662 | *FLJ22662* | 5.0 | 0.0028994 | 0.0295512 |
| 203 | NM_001461.1 | Flavin containing monooxygenase 5 | *FMO5* | 5.0 | 9.38E-05 | 0.0047769 |
| 204 | NM_023944.1 | Cytochrome P450, family 4, subfamily F, polypeptide 12 | *CYP4F12* | 5.0 | 0.0005105 | 0.0115475 |
| 205 | NM_138933.1 | Apobec-1 complementation factor , transcript variant 3 | *ACF* | 5.0 | 2.87E-06 | 0.0008457 |
| 206 | NM_198998.1 | Aquaporin 12A | *AQP12A* | 5.0 | 0.0051261 | 0.0411079 |
| 207 | NM_016323.1 | Hect domain and RLD 5 | *HERC5* | 5.0 | 0.0002183 | 0.0076103 |
| 208 | NM_006864.1 | Leukocyte immunoglobulin-like receptor, subfamily B , member 3 | *LILRB3* | 5.0 | 1.18E-06 | 0.0006013 |
| 209 | NM_133367.2 | Progestin and adipoq receptor family member VIII | *PAQR8* | 5.0 | 0.0009779 | 0.0165966 |
| 210 | NM_017817.1 | RAB20, member RAS oncogene family | *RAB20* | 4.9 | 0.0002266 | 0.0078091 |
| 211 | NM_031311.2 | Carboxypeptidase, vitellogenic-like , transcript variant 1 | *CPVL* | 4.9 | 0.0011213 | 0.0178875 |
| 212 | NM_005461.3 | V-maf musculoaponeurotic fibrosarcoma oncogene homolog B | *MAFB* | 4.9 | 3.32E-05 | 0.0029455 |
| 213 | NM_000531.3 | Ornithine carbamoyltransferase | *OTC* | 4.9 | 0.00577 | 0.0439543 |
| 214 | NM_032501.2 | Acyl-coa synthetase short-chain family member 1 , nuclear gene encoding mitochondrial protein | *ACSS1* | 4.9 | 3.93E-05 | 0.0031768 |
| 215 | NM_144765.1 | Epithelial V-like antigen 1 , transcript variant 2 | *EVA1* | 4.9 | 0.0014972 | 0.0208143 |
| 216 | NM_001005915.1 | V-erb-b2 erythroblastic leukemia viral oncogene homolog 3 , transcript variant s | *ERBB3* | 4.9 | 0.0001718 | 0.0067823 |
| 217 | NM_004079.3 | Cathepsin S | *CTSS* | 4.9 | 1.06E-05 | 0.0016716 |
| 218 | NM_005335.3 | Hematopoietic cell-specific Lyn substrate 1 | *HCLS1* | 4.9 | 0.0005725 | 0.0123845 |
| 219 | NM_006332.3 | Interferon, gamma-inducible protein 30 | *IFI30* | 4.8 | 1.55E-05 | 0.0021017 |
| 220 | NM_000716.3 | Complement component 4 binding protein, beta , transcript variant 1 | *C4BPB* | 4.8 | 0.0002622 | 0.0084024 |
| 221 | NM_014234.3 | Hydroxysteroid dehydrogenase 8 | *HSD17B8* | 4.8 | 7.19E-05 | 0.004169 |
| 222 | NM_001075.2 | UDP glucuronosyltransferase 2 family, polypeptide B10 | *UGT2B10* | 4.8 | 0.0004745 | 0.0112321 |
| 223 | NM_020247.3 | Chaperone, ABC1 activity of bc1 complex like | *CABC1* | 4.8 | 0.0002389 | 0.0080294 |
| 224 | XM_941789.1 | PREDICTED: similar to Glutathione S-transferase A1 | *LOC652358* | 4.8 | 0.0041593 | 0.0364948 |
| 225 | NM_003645.2 | Solute carrier family 27 , member 2 | *SLC27A2* | 4.8 | 0.0001995 | 0.0073225 |
| 226 | NM_001306.2 | Claudin 3 | *CLDN3* | 4.8 | 0.0056283 | 0.0434022 |
| 227 | XM_496697.2 | PREDICTED: similar to UDP-glucuronosyltransferase 2B7 precursor | *LOC441018* | 4.8 | 0.0018949 | 0.0234235 |
| 228 | BQ438671 | AGENCOURT_7908292 NIH_MGC_82 cdna clone IMAGE:6102595 5, mrna sequence | *HS.583806* | 4.7 | 0.0002811 | 0.0086625 |
| 229 | NM_000846.3 | Glutathione S-transferase A2 | *GSTA2* | 4.7 | 0.0039643 | 0.0355304 |
| 230 | NM_014266.3 | Hematopoietic cell signal transducer , transcript variant 1 | *HCST* | 4.7 | 0.0007334 | 0.0141769 |
| 231 | NM_003774.3 | UDP-N-acetyl-alpha-D-galactosamine:polypeptide N-acetylgalactosaminyltransferase 4 | *GALNT4* | 4.7 | 0.000315 | 0.0091874 |
| 232 | NM_001003954.1 | Annexin A13 , transcript variant 2 | *ANXA13* | 4.7 | 0.0007249 | 0.0141441 |
| 233 | NM_138393.1 | Receptor accessory protein 6 | *REEP6* | 4.7 | 0.0019697 | 0.0239493 |
| 234 | NM_021220.2 | Ovo-like 2 | *OVOL2* | 4.7 | 0.0066012 | 0.0473885 |
| 235 | NM_001001435.2 | Chemokine ligand 4-like 1 | *CCL4L1* | 4.6 | 0.0005741 | 0.0123865 |
| 236 | NM_000492.2 | Cystic fibrosis transmembrane conductance regulator, ATP-binding cassette | *CFTR* | 4.6 | 0.0034013 | 0.0323901 |
| 237 | NM_006144.2 | Granzyme A | *GZMA* | 4.6 | 1.49E-08 | 7.67E-05 |
| 238 | NM_033086.1 | FYVE, rhogef and PH domain containing 3 | *FGD3* | 4.6 | 0.0001019 | 0.0050215 |
| 239 | NM_001024912.1 | Carcinoembryonic antigen-related cell adhesion molecule 1 , transcript variant 2 | *CEACAM1* | 4.6 | 0.0080154 | 0.0529057 |
| 240 | NM_025195.2 | Tribbles homolog 1 | *TRIB1* | 4.6 | 5.04E-05 | 0.0036166 |
| 241 | NM_006059.2 | Laminin, gamma 3 | *LAMC3* | 4.6 | 0.0017956 | 0.0229601 |
| 242 | NM_002612.2 | Pyruvate dehydrogenase kinase, isozyme 4 | *PDK4* | 4.6 | 0.0088803 | 0.0558108 |
| 243 | NM_016339.1 | Rap guanine nucleotide exchange factor -like 1 | *RAPGEFL1* | 4.5 | 0.0002782 | 0.0086178 |
| 244 | NM_001015001.1 | Creatine kinase, mitochondrial 1A , nuclear gene encoding mitochondrial protein | *CKMT1A* | 4.5 | 0.0018886 | 0.0233751 |
| 245 | NM_000397.2 | Cytochrome b-245, beta polypeptide | *CYBB* | 4.5 | 1.44E-05 | 0.0020406 |
| 246 | NM_014585.3 | Solute carrier family 40 , member 1 | *SLC40A1* | 4.5 | 0.0002525 | 0.0082221 |
| 247 | NM_001251.1 | CD68 antigen | *CD68* | 4.5 | 0.000102 | 0.0050215 |
| 248 | NM_018490.1 | Leucine-rich repeat-containing G protein-coupled receptor 4 | *LGR4* | 4.5 | 2.11E-06 | 0.0007445 |
| 249 | NM_052972.2 | Leucine-rich alpha-2-glycoprotein 1 | *LRG1* | 4.5 | 0.0037409 | 0.0342878 |
| 250 | NM_015888.3 | Hook homolog 1 | *HOOK1* | 4.5 | 0.0005713 | 0.0123845 |
| 251 | NM_020998.2 | Macrophage stimulating 1 | *MST1* | 4.4 | 0.0001845 | 0.0070581 |
| 252 | NM_004951.3 | Epstein-Barr virus induced gene 2 | *EBI2* | 4.4 | 4.64E-05 | 0.0035072 |
| 253 | NM_001077.2 | UDP glucuronosyltransferase 2 family, polypeptide B17 | *UGT2B17* | 4.4 | 0.0001738 | 0.0067929 |
| 254 | NM_013314.2 | B-cell linker | *BLNK* | 4.4 | 0.0007956 | 0.0147091 |
| 255 | NM_000096.1 | Ceruloplasmin | *CP* | 4.4 | 0.0044947 | 0.038154 |
| 256 | NM_005693.1 | Nuclear receptor subfamily 1, group H, member 3 | *NR1H3* | 4.4 | 1.28E-05 | 0.0019228 |
| 257 | XM_941665.1 | PREDICTED: hypothetical LOC387763 | *LOC387763* | 4.3 | 0.0050513 | 0.040797 |
| 258 | NM_003120.1 | Spleen focus forming virus proviral integration oncogene spi1 | *SPI1* | 4.3 | 7.22E-06 | 0.0013593 |
| 259 | NM_018593.3 | Solute carrier family 16 , member 10 | *SLC16A10* | 4.3 | 0.0012146 | 0.0186709 |
| 260 | NM_203416.1 | CD163 antigen , transcript variant 2 | *CD163* | 4.3 | 0.0002415 | 0.0080666 |
| 261 | NM_006472.1 | Thioredoxin interacting protein | *TXNIP* | 4.3 | 0.0027032 | 0.0283983 |
| 262 | NM_000130.2 | Coagulation factor V | *F5* | 4.3 | 0.0001921 | 0.007195 |
| 263 | NM_005615.2 | Ribonuclease, rnase A family, k6 | *RNASE6* | 4.3 | 2.32E-05 | 0.0025823 |
| 264 | NM_015393.2 | DKFZP564O0823 protein | *DKFZP564O0823* | 4.3 | 0.0045048 | 0.0381603 |
| 265 | XM_938054.1 | PREDICTED: hypothetical gene supported by BC052596 | *LOC388564* | 4.2 | 0.0032414 | 0.0315434 |
| 266 | AV735490 | AV735490 CB cdna clone CBFBDD05 5, mrna sequence | *HS.580229* | 4.2 | 0.000454 | 0.0110132 |
| 267 | NM_031469.1 | SH3 domain binding glutamic acid-rich protein like 2 | *SH3BGRL2* | 4.2 | 0.0031337 | 0.0309163 |
| 268 | NM_152637.1 | Methyltransferase like 7B | *METTL7B* | 4.2 | 2.05E-05 | 0.0024201 |
| 269 | NM_007127.1 | Villin 1 | *VIL1* | 4.2 | 0.0070617 | 0.0492594 |
| 270 | NM_014430.1 | Cell death-inducing DFFA-like effector b | *CIDEB* | 4.2 | 0.0004625 | 0.0111166 |
| 271 | NM_198053.1 | CD3Z antigen, zeta polypeptide , transcript variant 1 | *CD3Z* | 4.2 | 5.90E-07 | 0.0005177 |
| 272 | NM_033255.2 | Epithelial stromal interaction 1 , transcript variant 2 | *EPSTI1* | 4.1 | 0.0002734 | 0.0085806 |
| 273 | NM_007029.2 | Stathmin-like 2 | *STMN2* | 4.1 | 0.0030337 | 0.0304391 |
| 274 | NM_015419.1 | Matrix-remodelling associated 5 | *MXRA5* | 4.1 | 0.0041026 | 0.0362321 |
| 275 | NM_005258.2 | GTP cyclohydrolase I feedback regulator | *GCHFR* | 4.1 | 0.0086425 | 0.0550967 |
| 276 | NM_015136.2 | Stabilin 1 | *STAB1* | 4.1 | 8.73E-05 | 0.0046429 |
| 277 | NM_005949.1 | Metallothionein 1F | *MT1F* | 4.1 | 0.002287 | 0.0258872 |
| 278 | NM_004288.3 | Pleckstrin homology, Sec7 and coiled-coil domains, binding protein | *PSCDBP* | 4.1 | 4.83E-05 | 0.003528 |
| 279 | NM_004946.1 | Dedicator of cytokinesis 2 | *DOCK2* | 4.1 | 3.01E-05 | 0.0028431 |
| 280 | NM_002661.1 | Phospholipase C, gamma 2 | *PLCG2* | 4.1 | 6.82E-05 | 0.0040836 |
| 281 | NM_020990.3 | Creatine kinase, mitochondrial 1B , nuclear gene encoding mitochondrial protein | *CKMT1B* | 4.1 | 0.0018793 | 0.0233237 |
| 282 | NM_002763.3 | Prospero-related homeobox 1 | *PROX1* | 4.0 | 0.0009217 | 0.0159791 |
| 283 | NM_006633.1 | IQ motif containing gtpase activating protein 2 | *IQGAP2* | 4.0 | 9.07E-06 | 0.0016192 |
| 284 | NM_174918.1 | Mast cell-expressed membrane protein 1 | *MCEMP1* | 4.0 | 0.0073945 | 0.0506156 |
| 285 | NM_175571.2 | Gtpase, IMAP family member 8 | *GIMAP8* | 4.0 | 0.0001187 | 0.0055009 |
| 286 | NM_017786.2 | Hypothetical protein FLJ20366 | *FLJ20366* | 4.0 | 0.0040415 | 0.0359301 |
| 287 | NM_021784.3 | Forkhead box A2 , transcript variant 1 | *FOXA2* | 4.0 | 0.0016758 | 0.0220421 |
| 288 | NM_001767.2 | CD2 antigen , sheep red blood cell receptor | *CD2* | 4.0 | 3.12E-05 | 0.0028582 |
| 289 | NM_005623.2 | Chemokine ligand 8 | *CCL8* | 4.0 | 0.0019016 | 0.0234635 |
| 290 | NM_152309.2 | Phosphoinositide-3-kinase adaptor protein 1 | *PIK3AP1* | 4.0 | 0.0017199 | 0.0223797 |
| 291 | BC035116 | Cdna clone IMAGE:5263177 | *HS.19339* | 4.0 | 0.0002225 | 0.0077066 |
| 292 | NM_000442.2 | Platelet/endothelial cell adhesion molecule | *PECAM1* | 4.0 | 4.34E-06 | 0.0010797 |
| 293 | NM_001776.3 | Ectonucleoside triphosphate diphosphohydrolase 1 | *ENTPD1* | 4.0 | 1.75E-05 | 0.0023005 |
| 294 | NM_021969.1 | Nuclear receptor subfamily 0, group B, member 2 | *NR0B2* | 4.0 | 0.0019811 | 0.0239775 |
| 295 | NM_014141.3 | Contactin associated protein-like 2 | *CNTNAP2* | 4.0 | 0.0085192 | 0.0547133 |
| 296 | NM_001467.3 | Solute carrier family 37 , member 4 | *SLC37A4* | 4.0 | 0.0003002 | 0.0089558 |
| 297 | NM_003294.3 | Tryptase alpha/beta 1 | *TPSAB1* | 4.0 | 0.0027822 | 0.0288554 |
| 298 | NM_000247.1 | MHC class I polypeptide-related sequence A | *MICA* | -4.0 | 0.0027865 | 0.0288775 |
| 299 | NM_145244.2 | DNA-damage-inducible transcript 4-like | *DDIT4L* | -4.0 | 0.0019033 | 0.0234743 |
| 300 | NM_017420.2 | Sine oculis homeobox homolog 4 | *SIX4* | -4.0 | 0.0001163 | 0.0054659 |
| 301 | NM_052839.2 | Pannexin 2 | *PANX2* | -4.0 | 0.0007162 | 0.0140247 |
| 302 | NM_003596.2 | Tyrosylprotein sulfotransferase 1 | *TPST1* | -4.0 | 0.0049835 | 0.0404664 |
| 303 | NM_052947.2 | Heart alpha-kinase | *HAK* | -4.0 | 0.0075176 | 0.0510192 |
| 304 | NM_013451.2 | Fer-1-like 3, myoferlin , transcript variant 1 | *FER1L3* | -4.0 | 0.0002766 | 0.0086154 |
| 305 | NM_007085.3 | Follistatin-like 1 | *FSTL1* | -4.0 | 0.0074643 | 0.0508771 |
| 306 | NM_207371.2 | FLJ45187 protein | *FLJ45187* | -4.0 | 0.0005895 | 0.012606 |
| 307 | NM_002064.1 | Glutaredoxin | *GLRX* | -4.1 | 0.0001728 | 0.0067894 |
| 308 | NM_012068.2 | Activating transcription factor 5 | *ATF5* | -4.1 | 0.0068359 | 0.0482557 |
| 309 | NM_002430.2 | Meningioma 1 | *MN1* | -4.1 | 0.0013252 | 0.0195327 |
| 310 | NM_033184.2 | Keratin associated protein 2-4 | *KRTAP2-4* | -4.1 | 0.0057712 | 0.0439543 |
| 311 | NM_000992.2 | Ribosomal protein L29 | *RPL29* | -4.2 | 0.0033561 | 0.0321172 |
| 312 | NM_022475.1 | Hedgehog interacting protein | *HHIP* | -4.2 | 0.0001932 | 0.0072166 |
| 313 | NM_203394.1 | E2F transcription factor 7 | *E2F7* | -4.2 | 6.09E-05 | 0.0039049 |
| 314 | NM_024692.3 | Restin-like 2 | *RSNL2* | -4.2 | 0.0001308 | 0.0057963 |
| 315 | NM_014365.2 | Heat shock 22kda protein 8 | *HSPB8* | -4.2 | 0.0044207 | 0.0378458 |
| 316 | NM_014903.3 | Neuron navigator 3 | *NAV3* | -4.2 | 2.13E-05 | 0.0024707 |
| 317 | NM_013943.1 | Chloride intracellular channel 4 | *CLIC4* | -4.2 | 0.0010428 | 0.0172024 |
| 318 | NM_005953.2 | Metallothionein 2A | *MT2A* | -4.2 | 0.001983 | 0.0239775 |
| 319 | NM_078467.1 | Cyclin-dependent kinase inhibitor 1A , transcript variant 2 | *CDKN1A* | -4.2 | 0.0004952 | 0.0114111 |
| 320 | NM_001235.2 | Serpin peptidase inhibitor, clade H , member 1, | *SERPINH1* | -4.3 | 0.0042161 | 0.0367627 |
| 321 | NM_002300.3 | Lactate dehydrogenase B | *LDHB* | -4.3 | 0.0065512 | 0.0471928 |
| 322 | NM_017946.2 | FK506 binding protein 14, 22 kda | *FKBP14* | -4.3 | 8.11E-05 | 0.0044556 |
| 323 | NM_032211.6 | Lysyl oxidase-like 4 | *LOXL4* | -4.3 | 0.0030203 | 0.0303631 |
| 324 | NM_003798.1 | Catenin , alpha-like 1 | *CTNNAL1* | -4.3 | 0.0001033 | 0.0050775 |
| 325 | NM_005168.3 | Rho family gtpase 3 | *RND3* | -4.4 | 5.68E-06 | 0.0012279 |
| 326 | NM_080655.1 | Chromosome 9 open reading frame 30 | *C9ORF30* | -4.4 | 2.61E-06 | 0.0008237 |
| 327 | NM_206833.1 | Cortexin 1 | *CTXN1* | -4.4 | 0.0036828 | 0.033995 |
| 328 | NM_022356.2 | Leucine proline-enriched proteoglycan 1 | *LEPRE1* | -4.4 | 0.0003678 | 0.0099631 |
| 329 | XM_940079.1 | PREDICTED: tubulin, beta 6 | *TUBB6* | -4.4 | 0.0002845 | 0.0087177 |
| 330 | NM_152359.1 | Carnitine palmitoyltransferase 1C | *CPT1C* | -4.5 | 0.0009439 | 0.0161717 |
| 331 | NM_001699.3 | AXL receptor tyrosine kinase , transcript variant 2 | *AXL* | -4.5 | 0.0032601 | 0.0316232 |
| 332 | NM_005458.5 | Gamma-aminobutyric acid B receptor, 2 | *GABBR2* | -4.5 | 0.0025613 | 0.027631 |
| 333 | NM_020814.1 | Membrane-associated ring finger 4 | *04-Mar* | -4.5 | 0.0001937 | 0.0072198 |
| 334 | NM_003246.2 | Thrombospondin 1 | *THBS1* | -4.5 | 0.0004727 | 0.0112321 |
| 335 | NM_145753.1 | Pleckstrin homology-like domain, family B, member 2 | *PHLDB2* | -4.5 | 0.0002752 | 0.0086092 |
| 336 | NM_002526.1 | 5'-nucleotidase, ecto | *NT5E* | -4.5 | 0.0009369 | 0.0161183 |
| 337 | NM_013363.2 | Procollagen C-endopeptidase enhancer 2 | *PCOLCE2* | -4.6 | 0.0030204 | 0.0303631 |
| 338 | NM_000700.1 | Annexin A1 | *ANXA1* | -4.6 | 0.0045044 | 0.0381603 |
| 339 | NM_014601.2 | EH-domain containing 2 | *EHD2* | -4.6 | 0.0001576 | 0.0064512 |
| 340 | NM_003486.5 | Solute carrier family 7 , member 5 | *SLC7A5* | -4.6 | 0.0001772 | 0.0068827 |
| 341 | NM_000963.1 | Prostaglandin-endoperoxide synthase 2 | *PTGS2* | -4.7 | 0.0012741 | 0.0191438 |
| 342 | NM_003632.1 | Contactin associated protein 1 | *CNTNAP1* | -4.7 | 0.0007277 | 0.0141588 |
| 343 | NM_006851.1 | GLI pathogenesis-related 1 | *GLIPR1* | -4.7 | 0.0003362 | 0.0094774 |
| 344 | NM_182507.1 | Hypothetical protein LOC144501 | *LOC144501* | -4.8 | 0.0068451 | 0.0483055 |
| 345 | NM_001554.3 | Cysteine-rich, angiogenic inducer, 61 | *CYR61* | -4.9 | 0.0005159 | 0.0116401 |
| 346 | NM_006206.3 | Platelet-derived growth factor receptor, alpha polypeptide | *PDGFRA* | -4.9 | 0.0060692 | 0.0452224 |
| 347 | NM_005429.2 | Vascular endothelial growth factor C | *VEGFC* | -4.9 | 0.0012043 | 0.0186087 |
| 348 | NM_006825.2 | Cytoskeleton-associated protein 4 | *CKAP4* | -4.9 | 4.07E-05 | 0.0032504 |
| 349 | NM_005279.2 | G protein-coupled receptor 1 | *GPR1* | -5.0 | 0.0035321 | 0.0331811 |
| 350 | NM_030786.1 | Syncoilin, intermediate filament 1 | *SYNC1* | -5.0 | 0.0014926 | 0.0207812 |
| 351 | NM_052913.2 | Kiaa1913 | *KIAA1913* | -5.0 | 0.0009445 | 0.0161717 |
| 352 | CD640673 | AGENCOURT_14535501 NIH_MGC_191 cdna clone IMAGE:30415823 5, mrna sequence | *HS.543887* | -5.2 | 1.34E-08 | 7.67E-05 |
| 353 | NM_024563.2 | Hypothetical protein FLJ14054 | *FLJ14054* | -5.3 | 0.0004292 | 0.0108174 |
| 354 | NM_014286.2 | Frequenin homolog | *FREQ* | -5.3 | 4.95E-06 | 0.0011448 |
| 355 | NM_006216.2 | Serpin peptidase inhibitor, clade E , member 2 | *SERPINE2* | -5.3 | 0.0010166 | 0.0169564 |
| 356 | NM_007026.1 | Dual specificity phosphatase 14 | *DUSP14* | -5.3 | 2.40E-06 | 0.0008005 |
| 357 | NM_002373.4 | Microtubule-associated protein 1A | *MAP1A* | -5.4 | 0.0013351 | 0.0196084 |
| 358 | XM_927536.1 | PREDICTED: aldehyde dehydrogenase 1 family, member L2 | *ALDH1L2* | -5.5 | 0.0043156 | 0.0373464 |
| 359 | NM_002658.2 | Plasminogen activator, urokinase | *PLAU* | -5.6 | 0.0044889 | 0.0381528 |
| 360 | NM_001451.1 | Forkhead box F1 | *FOXF1* | -5.6 | 0.0004521 | 0.0110078 |
| 361 | NM_173213.1 | Keratin 23 , transcript variant 2 | *KRT23* | -5.7 | 0.0066876 | 0.0475793 |
| 362 | NM_013409.1 | Follistatin , transcript variant FST344 | *FST* | -5.7 | 0.0069086 | 0.0486044 |
| 363 | NM_019102.2 | Homeobox A5 | *HOXA5* | -5.7 | 0.0065274 | 0.0471331 |
| 364 | NM_005504.4 | Branched chain aminotransferase 1, cytosolic | *BCAT1* | -5.8 | 0.0043153 | 0.0373464 |
| 365 | NM_001001991.1 | Regeneration associated muscle protease , transcript variant 2 | *DKFZP586H2123* | -6.0 | 0.0073797 | 0.0505547 |
| 366 | NM_006307.2 | Sushi-repeat-containing protein, X-linked | *SRPX* | -6.1 | 0.0024584 | 0.0270482 |
| 367 | NM_001628.2 | Aldo-keto reductase family 1, member B1 | *AKR1B1* | -6.1 | 0.0005778 | 0.0124477 |
| 368 | NM_000641.2 | Interleukin 11 | *IL11* | -6.3 | 7.62E-06 | 0.0014162 |
| 369 | NM_152330.2 | FERM domain containing 6 | *FRMD6* | -6.5 | 0.0009865 | 0.016638 |
| 370 | NM_138440.1 | Slit-like 2 | *SLITL2* | -6.5 | 0.0024298 | 0.0268757 |
| 371 | XM_934895.1 | PREDICTED: similar to keratin 17, transcript variant 3 | *LOC440421* | -6.5 | 0.0066872 | 0.0475793 |
| 372 | NM_001008397.1 | Similar to RIKEN cdna 2310016C16 | *LOC493869* | -6.6 | 0.0002934 | 0.0088416 |
| 373 | NM_002982.3 | Chemokine ligand 2 | *CCL2* | -6.7 | 0.003253 | 0.0315787 |
| 374 | NM_021013.3 | Keratin, hair, acidic, 4 | *KRTHA4* | -6.8 | 0.0051248 | 0.0411079 |
| 375 | NM_002781.2 | Pregnancy specific beta-1-glycoprotein 5 | *PSG5* | -6.8 | 0.0004275 | 0.0108127 |
| 376 | NM_003330.2 | Thioredoxin reductase 1 , transcript variant 1 | *TXNRD1* | -6.9 | 6.41E-07 | 0.0005177 |
| 377 | NM_058179.2 | Phosphoserine aminotransferase 1 , transcript variant 1 | *PSAT1* | -6.9 | 0.0006932 | 0.0138048 |
| 378 | XM_498969.2 | PREDICTED: hypothetical LOC441019 | *LOC441019* | -7.0 | 0.0002766 | 0.0086154 |
| 379 | NM_144617.1 | Heat shock protein, alpha-crystallin-related, B6 | *HSPB6* | -7.0 | 0.0031221 | 0.0308747 |
| 380 | NM_197958.1 | La ribonucleoprotein domain family, member 6 , transcript variant 2 | *LARP6* | -7.1 | 0.0001148 | 0.0054244 |
| 381 | NM_014241.2 | Protein tyrosine phosphatase-like , member a | *PTPLA* | -7.2 | 6.49E-05 | 0.0040208 |
| 382 | XM_087386.8 | PREDICTED: HEG homolog 1 | *HEG1* | -7.4 | 0.0015096 | 0.020887 |
| 383 | NM_004932.2 | Cadherin 6, type 2, K-cadherin | *CDH6* | -7.5 | 3.62E-05 | 0.00305 |
| 384 | NM_006086.2 | Tubulin, beta 3 | *TUBB3* | -7.6 | 0.0018495 | 0.0231938 |
| 385 | NM_001958.2 | Eukaryotic translation elongation factor 1 alpha 2 | *EEF1A2* | -8.0 | 0.0022402 | 0.0255741 |
| 386 | NM_004750.2 | Cytokine receptor-like factor 1 | *CRLF1* | -8.1 | 6.22E-05 | 0.003948 |
| 387 | NM_006042.1 | Heparan sulfate 3-O-sulfotransferase 3A1 | *HS3ST3A1* | -8.1 | 2.10E-05 | 0.0024522 |
| 388 | XM_938715.1 | PREDICTED: hyaluronan synthase 2 | *HAS2* | -8.4 | 8.45E-07 | 0.0005331 |
| 389 | NM_004472.1 | Forkhead box D1 | *FOXD1* | -8.7 | 0.0011154 | 0.0178367 |
| 390 | NM_003012.3 | Secreted frizzled-related protein 1 | *SFRP1* | -9.5 | 0.0003217 | 0.0092982 |
| 391 | NM_001031692.1 | Leucine rich repeat containing 17 , transcript variant 1 | *LRRC17* | -9.8 | 2.51E-05 | 0.0026584 |
| 392 | NM_013372.5 | Gremlin 1, cysteine knot superfamily, homolog | *GREM1* | -10.0 | 0.0012765 | 0.0191598 |
| 393 | NM_001878.2 | Cellular retinoic acid binding protein 2 | *CRABP2* | -10.2 | 7.08E-05 | 0.0041469 |
| 394 | NM_000710.2 | Bradykinin receptor B1 | *BDKRB1* | -10.3 | 1.45E-05 | 0.0020416 |
| 395 | NM_001458.2 | Filamin C, gamma | *FLNC* | -10.3 | 3.29E-05 | 0.0029374 |
| 396 | NM_001323.2 | Cystatin E/M | *CST6* | -10.4 | 0.0016168 | 0.021643 |
| 397 | NM_080927.3 | Discoidin, CUB and LCCL domain containing 2 | *DCBLD2* | -10.5 | 6.94E-05 | 0.0040925 |
| 398 | NM_002581.3 | Pregnancy-associated plasma protein A, pappalysin 1 | *PAPPA* | -10.9 | 0.0001174 | 0.0054731 |
| 399 | XM_937100.1 | PREDICTED: keratin associated protein 2-1, transcript variant 3 | *KRTAP2-1* | -11.3 | 0.0001969 | 0.0072816 |
| 400 | NM_002704.2 | Pro-platelet basic protein ligand 7) | *PPBP* | -11.6 | 0.0092201 | 0.0569149 |
| 401 | NM_003714.2 | Stanniocalcin 2 | *STC2* | -11.8 | 3.73E-05 | 0.0031017 |
| 402 | NM_005613.3 | Regulator of G-protein signalling 4 | *RGS4* | -12.0 | 9.98E-06 | 0.001656 |
| 403 | NM_002178.2 | Insulin-like growth factor binding protein 6 | *IGFBP6* | -13.0 | 0.0006727 | 0.0135451 |
| 404 | NM_002317.3 | Lysyl oxidase | *LOX* | -13.3 | 0.0002356 | 0.007969 |
| 405 | NM_004598.2 | Sparc/osteonectin, cwcv and kazal-like domains proteoglycan | *SPOCK* | -13.6 | 0.0003278 | 0.0093462 |
| 406 | NM_012242.2 | Dickkopf homolog 1 | *DKK1* | -14.1 | 0.0007496 | 0.0143321 |
| 407 | NM_000600.1 | Interleukin 6 | *IL6* | -14.2 | 0.0001714 | 0.0067749 |
| 408 | NM_153360.1 | Hypothetical protein FLJ90166 | *FLJ90166* | -14.4 | 0.0001074 | 0.0052011 |
| 409 | NM_001792.2 | Cadherin 2, type 1, N-cadherin | *CDH2* | -15.9 | 0.0006107 | 0.0128001 |
| 410 | NM_006528.2 | Tissue factor pathway inhibitor 2 | *TFPI2* | -17.1 | 0.0002148 | 0.0075745 |
| 411 | NM_006818.3 | Myeloid/lymphoid or mixed-lineage leukemia ; translocated to, 11 | *MLLT11* | -17.9 | 6.16E-07 | 0.0005177 |
| 412 | NM_007281.1 | Scrapie responsive protein 1 | *SCRG1* | -18.1 | 0.0003639 | 0.009939 |
| 413 | NM_000602.1 | Serpin peptidase inhibitor, clade E , member 1 | *SERPINE1* | -18.8 | 2.29E-05 | 0.0025793 |
| 414 | NM_002852.2 | Pentraxin-related gene, rapidly induced by IL-1 beta | *PTX3* | -19.3 | 0.0002407 | 0.0080659 |
| 415 | NM_003480.2 | Microfibrillar associated protein 5 | *MFAP5* | -21.3 | 0.0001319 | 0.0058189 |
| 416 | NM_153370.2 | Peptidase inhibitor 16 | *PI16* | -23.9 | 9.37E-05 | 0.0047769 |
| 417 | NM_004181.3 | Ubiquitin carboxyl-terminal esterase L1 | *UCHL1* | -27.7 | 3.15E-05 | 0.002872 |
| 418 | NM_014391.2 | Ankyrin repeat domain 1 | *ANKRD1* | -32.4 | 2.77E-07 | 0.0004472 |
| 419 | NM_002575.1 | Serpin peptidase inhibitor, clade B , member 2 | *SERPINB2* | -35.6 | 1.84E-05 | 0.0023392 |
| 420 | NM_000693.1 | Aldehyde dehydrogenase 1 family, member A3 | *ALDH1A3* | -50.5 | 4.01E-06 | 0.0010164 |

A univariate *t*-test based on 10,000 random permutations in R packages was used to statistically analyze the differentially expressed genes. Genes with a *q*-value < 0.057 and with a mean difference > 4 were selected.
