# Supplementary material for: Genome-wide expression patterns associated with oncogenesis and sarcomatous transdifferentation of cholangiocarcinoma
Source: BMC Cancer. 2011 Feb 19;11:78. doi: 10.1186/1471-2407-11-78 (PMC3053267; doi:10.1186/1471-2407-11-78)
Supplement: Additional file 3 — Supplementary Table S3: List of genes differentially expressed between undifferentiated cholangiocarcinoma and normal biliary epithelium (NBE). [file 1471-2407-11-78-S3.DOC]

Supplementary Table 3: List of genes differentially expressed between undifferntiated cholangiocarcinoma and normal biliary epithelium (NBE).

| **Rank** | **Accession No.** | **Title** | **Symbol** | **Mean fold change** | ***P* Value** | ***q*-Value** |
| --- | --- | --- | --- | --- | --- | --- |
| 1 | NM_000477.3 | Albumin | *ALB* | 252.4 | 0 | 0 |
| 2 | NM_001643.1 | Apolipoprotein A-II | *APOA2* | 221.8 | 4.41E-08 | 2.21E-05 |
| 3 | NM_001063.2 | Transferrin | *TF* | 221.2 | 3.20E-09 | 3.41E-06 |
| 4 | NM_001633.2 | Alpha-1-microglobulin/bikunin precursor | *AMBP* | 199.4 | 3.40E-09 | 3.41E-06 |
| 5 | NM_005141.2 | Fibrinogen beta chain | *FGB* | 173.6 | 5.10E-09 | 4.93E-06 |
| 6 | NM_000607.1 | Orosomucoid 1 | *ORM1* | 153.4 | 2.54E-08 | 1.54E-05 |
| 7 | NM_000039.1 | Apolipoprotein A-I | *APOA1* | 150.7 | 8.30E-09 | 6.56E-06 |
| 8 | NM_000583.2 | Group-specific component | *GC* | 141.6 | 4.90E-08 | 2.30E-05 |
| 9 | NM_000638.2 | Vitronectin | *VTN* | 134.7 | 3.00E-10 | 7.12E-07 |
| 10 | NM_005143.2 | Haptoglobin | *HP* | 129.6 | 8.00E-09 | 6.56E-06 |
| 11 | NM_000508.3 | Fibrinogen alpha chain , transcript variant alpha-E | *FGA* | 123.7 | 0 | 0 |
| 12 | NM_000014.4 | Alpha-2-macroglobulin | *A2M* | 119.4 | 0 | 0 |
| 13 | NM_001622.1 | Alpha-2-HS-glycoprotein | *AHSG* | 98.9 | 2.35E-06 | 0.000258 |
| 14 | NM_001645.3 | Apolipoprotein C-I | *APOC1* | 98.2 | 0 | 0 |
| 15 | NM_000608.2 | Orosomucoid 2 | *ORM2* | 86.4 | 5.88E-07 | 0.0001088 |
| 16 | NM_000506.2 | Coagulation factor II | *F2* | 86.1 | 4.22E-07 | 8.79E-05 |
| 17 | NM_000384.1 | Apolipoprotein B antigen) | *APOB* | 84.8 | 2.55E-07 | 6.17E-05 |
| 18 | NM_000096.1 | Ceruloplasmin | *CP* | 79.5 | 0 | 0 |
| 19 | NM_000371.1 | Transthyretin | *TTR* | 79.3 | 7.48E-08 | 2.83E-05 |
| 20 | NM_000567.2 | C-reactive protein, pentraxin-related | *CRP* | 70.9 | 4.24E-06 | 0.0003463 |
| 21 | NM_021870.2 | Fibrinogen gamma chain , transcript variant gamma-B | *FGG* | 65.9 | 2.00E-10 | 5.22E-07 |
| 22 | NM_015991.1 | Complement component 1, q subcomponent, alpha polypeptide | *C1QA* | 65.7 | 1.00E-09 | 1.69E-06 |
| 23 | NM_001639.2 | Amyloid P component, serum | *APCS* | 63.5 | 7.60E-08 | 2.83E-05 |
| 24 | NM_000042.1 | Apolipoprotein H | *APOH* | 63.4 | 1.63E-07 | 4.77E-05 |
| 25 | NM_005410.2 | Selenoprotein P, plasma, 1 | *SEPP1* | 53.8 | 7.60E-09 | 6.56E-06 |
| 26 | NM_000715.3 | Complement component 4 binding protein, alpha | *C4BPA* | 53.1 | 8.35E-07 | 0.0001313 |
| 27 | NM_000488.2 | Serpin peptidase inhibitor, clade C , member 1 | *SERPINC1* | 49.9 | 1.34E-06 | 0.0001761 |
| 28 | NM_000491.2 | Complement component 1, q subcomponent, beta polypeptide | *C1QB* | 49.3 | 2.30E-09 | 2.86E-06 |
| 29 | NM_005518.2 | 3-hydroxy-3-methylglutaryl-Coenzyme A synthase 2 | *HMGCS2* | 44.9 | 5.59E-06 | 0.0003956 |
| 30 | NM_019111.3 | Major histocompatibility complex, class II, DR alpha | *HLA-DRA* | 44.2 | 0.000135 | 0.0028351 |
| 31 | NM_000239.1 | Lysozyme | *LYZ* | 44.2 | 5.89E-08 | 2.39E-05 |
| 32 | NM_000295.3 | Serpin peptidase inhibitor, clade A , member 1 , transcript variant 1 | *SERPINA1* | 42.9 | 3.03E-05 | 0.001133 |
| 33 | NM_000518.4 | Hemoglobin, beta | *HBB* | 42.8 | 2.24E-07 | 5.78E-05 |
| 34 | NM_000041.2 | Apolipoprotein E | *APOE* | 42.0 | 1.27E-05 | 0.0006634 |
| 35 | NM_001735.2 | Complement component 5 | *C5* | 42.0 | 6.02E-07 | 0.0001098 |
| 36 | NM_000040.1 | Apolipoprotein C-III | *APOC3* | 40.3 | 1.98E-06 | 0.0002289 |
| 37 | NM_003332.2 | TYRO protein tyrosine kinase binding protein , transcript variant 1 | *TYROBP* | 39.6 | 9.20E-09 | 6.86E-06 |
| 38 | NM_000035.2 | Aldolase B, fructose-bisphosphate | *ALDOB* | 39.3 | 1.91E-05 | 0.0008477 |
| 39 | XM_941953.1 | PREDICTED: similar to Ig kappa chain V-I region HK102 precursor | *LOC652493* | 36.8 | 1.98E-05 | 0.000865 |
| 40 | NM_004139.2 | Lipopolysaccharide binding protein | *LBP* | 36.5 | 1.70E-09 | 2.22E-06 |
| 41 | NM_006744.3 | Retinol binding protein 4, plasma | *RBP4* | 35.5 | 2.28E-06 | 0.0002512 |
| 42 | NM_000483.3 | Apolipoprotein C-II | *APOC2* | 35.3 | 9.68E-07 | 0.000146 |
| 43 | NM_172369.2 | Complement component 1, q subcomponent, C chain | *C1QC* | 34.8 | 1.20E-09 | 1.74E-06 |
| 44 | NM_031310.1 | Plasmalemma vesicle associated protein | *PLVAP* | 34.2 | 0 | 0 |
| 45 | NM_002216.2 | Inter-alpha inhibitor H2 | *ITIH2* | 32.5 | 9.55E-06 | 0.0005629 |
| 46 | NM_005211.2 | Colony stimulating factor 1 receptor, formerly mcdonough feline sarcoma viral oncogene homolog | *CSF1R* | 32.3 | 1.10E-09 | 1.69E-06 |
| 47 | NM_001671.2 | Asialoglycoprotein receptor 1 | *ASGR1* | 32.0 | 7.58E-07 | 0.000126 |
| 48 | NR_002196.1 | H19, imprinted maternally expressed untranslated mrna on chromosome 11. | *H19* | 31.8 | 6.68E-05 | 0.0018295 |
| 49 | NM_000029.2 | Angiotensinogen | *AGT* | 31.4 | 5.25E-07 | 0.0001008 |
| 50 | NM_000613.1 | Hemopexin | *HPX* | 31.4 | 6.59E-06 | 0.0004448 |
| 51 | NM_000893.2 | Kininogen 1 | *KNG1* | 30.9 | 1.08E-05 | 0.0006068 |
| 52 | NM_004364.2 | CCAAT/enhancer binding protein , alpha | *CEBPA* | 30.6 | 3.40E-09 | 3.41E-06 |
| 53 | XM_942302.1 | PREDICTED: similar to Ig kappa chain V-I region HK102 precursor | *LOC652694* | 30.3 | 5.89E-05 | 0.001705 |
| 54 | AK129542 | Cdna FLJ26031 fis, clone PNC08078 | *HS.223856* | 30.2 | 3.43E-06 | 0.0003141 |
| 55 | NM_000624.3 | Serpin peptidase inhibitor, clade A , member 5 | *SERPINA5* | 29.4 | 0.0002427 | 0.0040882 |
| 56 | NM_001872.2 | Carboxypeptidase B2 , transcript variant 1 | *CPB2* | 29.0 | 3.00E-06 | 0.0002952 |
| 57 | NM_021175.2 | Hepcidin antimicrobial peptide | *HAMP* | 28.5 | 6.35E-05 | 0.0017915 |
| 58 | NM_000030.1 | Alanine-glyoxylate aminotransferase | *AGXT* | 27.9 | 1.44E-05 | 0.0007161 |
| 59 | NM_000064.1 | Complement component 3 | *C3* | 27.6 | 1.41E-07 | 4.43E-05 |
| 60 | NM_000591.1 | CD14 antigen | *CD14* | 27.6 | 8.90E-09 | 6.83E-06 |
| 61 | NM_000354.3 | Serpin peptidase inhibitor, clade A , member 7 | *SERPINA7* | 26.6 | 1.05E-06 | 0.0001528 |
| 62 | NM_001756.3 | Serpin peptidase inhibitor, clade A , member 6 | *SERPINA6* | 25.9 | 4.56E-06 | 0.0003573 |
| 63 | NM_000582.2 | Secreted phosphoprotein 1 | *SPP1* | 25.6 | 2.26E-06 | 0.0002502 |
| 64 | NM_001073.1 | UDP glucuronosyltransferase 2 family, polypeptide B11 | *UGT2B11* | 24.9 | 2.00E-05 | 0.0008719 |
| 65 | NM_000211.1 | Integrin, beta 2 , lymphocyte function-associated antigen 1; macrophage antigen 1 beta subunit) | *ITGB2* | 24.5 | 2.08E-05 | 0.0008938 |
| 66 | NM_001443.1 | Fatty acid binding protein 1, liver | *FABP1* | 24.2 | 2.49E-05 | 0.0010067 |
| 67 | XM_936120.1 | PREDICTED: major histocompatibility complex, class II, DQ alpha 1, transcript variant 2 | *HLA-DQA1* | 24.2 | 7.79E-07 | 0.0001286 |
| 68 | NM_000596.2 | Insulin-like growth factor binding protein 1 , transcript variant 1 | *IGFBP1* | 23.8 | 2.28E-05 | 0.0009553 |
| 69 | NM_000773.3 | Cytochrome P450, family 2, subfamily E, polypeptide 1 | *CYP2E1* | 23.5 | 8.74E-05 | 0.0021694 |
| 70 | NM_002922.3 | Regulator of G-protein signalling 1 | *RGS1* | 23.5 | 1.01E-07 | 3.34E-05 |
| 71 | NM_152889.1 | Carbohydrate sulfotransferase 13 | *CHST13* | 23.5 | 1.99E-07 | 5.52E-05 |
| 72 | XM_939003.1 | PREDICTED: similar to Ig gamma-2 chain C region | *LOC649923* | 23.4 | 0.0001052 | 0.0024457 |
| 73 | NM_000667.2 | Alcohol dehydrogenase 1A , alpha polypeptide | *ADH1A* | 23.3 | 0.0012208 | 0.0116166 |
| 74 | NM_002298.2 | Lymphocyte cytosolic protein 1 , transcript variant 5 | *LCP1* | 23.0 | 1.00E-09 | 1.69E-06 |
| 75 | NM_000558.3 | Hemoglobin, alpha 1 | *HBA1* | 22.6 | 1.34E-06 | 0.0001761 |
| 76 | NM_006762.1 | Lysosomal associated multispanning membrane protein 5 | *LAPTM5* | 22.5 | 2.70E-09 | 3.20E-06 |
| 77 | XM_936253.1 | PREDICTED: similar to Ig kappa chain V-III region HAH precursor | *LOC642113* | 22.5 | 5.80E-05 | 0.001686 |
| 78 | NM_001482.1 | Glycine amidinotransferase | *GATM* | 22.5 | 1.97E-06 | 0.0002289 |
| 79 | NM_001074.1 | UDP glucuronosyltransferase 2 family, polypeptide B7 | *UGT2B7* | 22.5 | 2.81E-05 | 0.0010866 |
| 80 | NM_001737.2 | Complement component 9 | *C9* | 22.5 | 2.54E-05 | 0.0010135 |
| 81 | NM_002118.3 | Major histocompatibility complex, class II, DM beta | *HLA-DMB* | 22.5 | 1.26E-05 | 0.0006607 |
| 82 | NM_022349.2 | Membrane-spanning 4-domains, subfamily A, member 6A , transcript variant 2 | *MS4A6A* | 22.1 | 7.20E-09 | 6.48E-06 |
| 83 | NM_000063.3 | Complement component 2 | *C2* | 22.0 | 0 | 0 |
| 84 | NM_002215.1 | Inter-alpha inhibitor H1 | *ITIH1* | 21.8 | 1.51E-05 | 0.0007316 |
| 85 | NM_000045.2 | Arginase, liver | *ARG1* | 21.6 | 5.39E-05 | 0.0016085 |
| 86 | NM_000130.2 | Coagulation factor V | *F5* | 21.4 | 2.96E-07 | 6.84E-05 |
| 87 | NM_000606.1 | Complement component 8, gamma polypeptide | *C8G* | 21.1 | 3.06E-06 | 0.0002965 |
| 88 | NM_001075.2 | UDP glucuronosyltransferase 2 family, polypeptide B10 | *UGT2B10* | 20.5 | 4.55E-05 | 0.0014423 |
| 89 | NM_021139.1 | UDP glucuronosyltransferase 2 family, polypeptide B4 | *UGT2B4* | 20.3 | 4.48E-05 | 0.001442 |
| 90 | NM_002964.3 | S100 calcium binding protein A8 | *S100A8* | 20.2 | 1.52E-05 | 0.0007316 |
| 91 | XM_936518.1 | PREDICTED: similar to Ig kappa chain V-I region HK101 precursor | *LOC647450* | 20.0 | 6.68E-05 | 0.0018295 |
| 92 | NM_000778.2 | Cytochrome P450, family 4, subfamily A, polypeptide 11 | *CYP4A11* | 20.0 | 0.000123 | 0.0026993 |
| 93 | NM_002153.1 | Hydroxysteroid dehydrogenase 2 | *HSD17B2* | 19.9 | 1.51E-05 | 0.0007316 |
| 94 | NM_145740.2 | Glutathione S-transferase A1 | *GSTA1* | 19.8 | 0.0005382 | 0.0067923 |
| 95 | NM_033554.2 | Major histocompatibility complex, class II, DP alpha 1 | *HLA-DPA1* | 19.1 | 9.10E-05 | 0.0022282 |
| 96 | NM_000301.1 | Plasminogen | *PLG* | 18.9 | 7.54E-05 | 0.0019723 |
| 97 | NM_001025158.1 | CD74 antigen , transcript variant 3 | *CD74* | 18.7 | 0.0002709 | 0.0043375 |
| 98 | NM_014495.2 | Angiopoietin-like 3 | *ANGPTL3* | 18.6 | 1.06E-05 | 0.0005968 |
| 99 | NM_012072.2 | Complement component 1, q subcomponent, receptor 1 | *C1QR1* | 18.4 | 1.00E-10 | 2.90E-07 |
| 100 | NM_000562.1 | Complement component 8, alpha polypeptide | *C8A* | 17.9 | 6.39E-06 | 0.0004411 |
| 101 | NM_021983.4 | Major histocompatibility complex, class II, DR beta 4 | *HLA-DRB4* | 17.8 | 1.13E-05 | 0.0006241 |
| 102 | NM_032782.3 | Hepatitis A virus cellular receptor 2 | *HAVCR2* | 17.5 | 7.00E-10 | 1.52E-06 |
| 103 | NM_001085.4 | Serpin peptidase inhibitor, clade A , member 3 | *SERPINA3* | 17.3 | 2.66E-06 | 0.0002847 |
| 104 | XM_496697.2 | PREDICTED: similar to UDP-glucuronosyltransferase 2B7 precursor | *LOC441018* | 17.3 | 4.52E-05 | 0.001442 |
| 105 | NM_000846.3 | Glutathione S-transferase A2 | *GSTA2* | 17.1 | 0.0006189 | 0.0074573 |
| 106 | NM_001145.2 | Angiogenin, ribonuclease, rnase A family, 5 | *ANG* | 17.0 | 6.99E-06 | 0.0004563 |
| 107 | NM_004484.2 | Glypican 3 | *GPC3* | 16.9 | 0.0002228 | 0.003871 |
| 108 | NM_018419.2 | SRY -box 18 | *SOX18* | 16.7 | 3.94E-07 | 8.44E-05 |
| 109 | NM_000340.1 | Solute carrier family 2 , member 2 | *SLC2A2* | 16.6 | 3.40E-05 | 0.0012174 |
| 110 | NM_004132.2 | Hyaluronan binding protein 2 | *HABP2* | 16.6 | 6.51E-06 | 0.0004439 |
| 111 | XM_942072.1 | PREDICTED: carboxypeptidase N, polypeptide 2, 83kd | *CPN2* | 16.6 | 1.24E-05 | 0.0006564 |
| 112 | NM_018326.2 | Gtpase, IMAP family member 4 | *GIMAP4* | 16.3 | 1.60E-09 | 2.20E-06 |
| 113 | NM_005849.1 | Immunoglobulin superfamily, member 6 | *IGSF6* | 16.3 | 4.61E-08 | 2.27E-05 |
| 114 | XM_941789.1 | PREDICTED: similar to Glutathione S-transferase A1 | *LOC652358* | 16.0 | 0.0006469 | 0.0076742 |
| 115 | CR627122 | Mrna; cdna dkfzp779m2422 | *HS.291319* | 16.0 | 4.38E-07 | 8.85E-05 |
| 116 | NM_000185.3 | Serpin peptidase inhibitor, clade D , member 1 | *SERPIND1* | 15.9 | 1.69E-05 | 0.0007773 |
| 117 | NM_031311.2 | Carboxypeptidase, vitellogenic-like , transcript variant 1 | *CPVL* | 15.8 | 2.81E-07 | 6.55E-05 |
| 118 | XM_936226.1 | PREDICTED: similar to Complement C3 precursor | *LOC653879* | 15.8 | 1.19E-06 | 0.0001656 |
| 119 | NM_000412.2 | Histidine-rich glycoprotein | *HRG* | 15.7 | 0.0004239 | 0.0057563 |
| 120 | XM_937886.1 | PREDICTED: fibrinogen-like 1, transcript variant 5 | *FGL1* | 15.5 | 1.03E-06 | 0.000151 |
| 121 | NM_052972.2 | Leucine-rich alpha-2-glycoprotein 1 | *LRG1* | 15.4 | 1.26E-06 | 0.0001702 |
| 122 | NM_016347.1 | Putative N-acetyltransferase Camello 2 | *CML2* | 15.0 | 3.22E-06 | 0.0003002 |
| 123 | NM_000552.2 | Von Willebrand factor | *VWF* | 14.7 | 3.77E-06 | 0.0003281 |
| 124 | XM_940969.1 | PREDICTED: similar to Ig kappa chain V-II region RPMI 6410 precursor | *LOC651751* | 14.7 | 0.0003663 | 0.0052341 |
| 125 | NM_002113.1 | Complement factor H-related 1 | *CFHR1* | 14.6 | 0.0001227 | 0.0026962 |
| 126 | NM_206939.1 | Membrane-spanning 4-domains, subfamily A, member 7 , transcript variant 3 | *MS4A7* | 14.5 | 1.26E-08 | 8.89E-06 |
| 127 | NM_000065.1 | Complement component 6 | *C6* | 14.4 | 0.0007491 | 0.0084273 |
| 128 | NM_001818.2 | Aldo-keto reductase family 1, member C4 | *AKR1C4* | 14.3 | 3.83E-05 | 0.0013002 |
| 129 | NM_001077.2 | UDP glucuronosyltransferase 2 family, polypeptide B17 | *UGT2B17* | 14.2 | 8.76E-05 | 0.0021695 |
| 130 | XM_940101.1 | PREDICTED: serpin peptidase inhibitor, clade A , member 11 | *SERPINA11* | 14.0 | 0.0002206 | 0.0038509 |
| 131 | NM_177550.2 | Solute carrier family 13 , member 5 | *SLC13A5* | 14.0 | 0.0001241 | 0.0027114 |
| 132 | NM_000934.1 | Serpin peptidase inhibitor, clade F , member 2 | *SERPINF2* | 13.9 | 5.28E-05 | 0.0015951 |
| 133 | NM_014033.2 | Methyltransferase like 7A | *METTL7A* | 13.9 | 8.20E-05 | 0.0020704 |
| 134 | NM_018487.2 | Hepatocellular carcinoma-associated antigen 112 | *HCA112* | 13.6 | 5.33E-05 | 0.0016024 |
| 135 | NM_003167.2 | Sulfotransferase family, cytosolic, 2A, dehydroepiandrosterone -preferring, member 1 | *SULT2A1* | 13.6 | 0.0004428 | 0.0059338 |
| 136 | NM_001175.4 | Rho GDP dissociation inhibitor beta | *ARHGDIB* | 13.5 | 0.0004503 | 0.0060122 |
| 137 | NM_138933.1 | Apobec-1 complementation factor , transcript variant 3 | *ACF* | 13.4 | 4.46E-06 | 0.0003541 |
| 138 | NM_000067.1 | Carbonic anhydrase II | *CA2* | 13.4 | 8.80E-06 | 0.0005318 |
| 139 | NM_194431.1 | Ribonuclease, rnase A family, 4 , transcript variant 3 | *RNASE4* | 13.3 | 1.21E-05 | 0.0006477 |
| 140 | NM_022555.3 | Major histocompatibility complex, class II, DR beta 3 | *HLA-DRB3* | 13.2 | 6.99E-06 | 0.0004563 |
| 141 | NM_000716.3 | Complement component 4 binding protein, beta , transcript variant 1 | *C4BPB* | 13.0 | 1.19E-05 | 0.0006392 |
| 142 | NM_000133.2 | Coagulation factor IX | *F9* | 13.0 | 0.0002498 | 0.0041575 |
| 143 | NM_000253.1 | Microsomal triglyceride transfer protein | *MTTP* | 13.0 | 2.27E-05 | 0.0009546 |
| 144 | NM_000669.3 | Alcohol dehydrogenase 1C , gamma polypeptide | *ADH1C* | 13.0 | 0.0022466 | 0.0172365 |
| 145 | NM_006894.4 | Flavin containing monooxygenase 3 , transcript variant 1 | *FMO3* | 12.8 | 0.000303 | 0.0046382 |
| 146 | NM_000777.2 | Cytochrome P450, family 3, subfamily A, polypeptide 5 | *CYP3A5* | 12.8 | 1.95E-05 | 0.0008568 |
| 147 | NM_004106.1 | Fc fragment of ige, high affinity I, receptor for; gamma polypeptide | *FCER1G* | 12.7 | 5.44E-08 | 2.39E-05 |
| 148 | NM_000277.1 | Phenylalanine hydroxylase | *PAH* | 12.4 | 5.41E-05 | 0.0016112 |
| 149 | NM_005651.1 | Tryptophan 2,3-dioxygenase | *TDO2* | 12.4 | 3.77E-05 | 0.0012834 |
| 150 | NM_019101.2 | Apolipoprotein M | *APOM* | 12.4 | 0.0003416 | 0.0050265 |
| 151 | NM_000066.2 | Complement component 8, beta polypeptide | *C8B* | 12.0 | 0.0001681 | 0.0032482 |
| 152 | NM_002664.1 | Pleckstrin | *PLEK* | 12.0 | 7.39E-07 | 0.0001237 |
| 153 | NM_006183.3 | Neurotensin | *NTS* | 11.9 | 0.0037433 | 0.0240739 |
| 154 | NM_024027.3 | Collectin sub-family member 11 , transcript variant 1 | *COLEC11* | 11.9 | 4.32E-05 | 0.0014233 |
| 155 | NM_007256.2 | Solute carrier organic anion transporter family, member 2B1 | *SLCO2B1* | 11.8 | 2.12E-08 | 1.35E-05 |
| 156 | NM_015234.3 | G protein-coupled receptor 116 | *GPR116* | 11.7 | 3.86E-08 | 2.06E-05 |
| 157 | NM_000770.2 | Cytochrome P450, family 2, subfamily C, polypeptide 8 , transcript variant Hp1-1 | *CYP2C8* | 11.7 | 0.0014308 | 0.0128376 |
| 158 | NM_001701.2 | Bile acid Coenzyme A: amino acid N-acyltransferase | *BAAT* | 11.7 | 0.0013362 | 0.0123027 |
| 159 | NM_006120.2 | Major histocompatibility complex, class II, DM alpha | *HLA-DMA* | 11.5 | 4.46E-05 | 0.001442 |
| 160 | NM_002889.2 | Retinoic acid receptor responder 2 | *RARRES2* | 11.4 | 0.0002233 | 0.0038746 |
| 161 | NM_053039.1 | UDP glucuronosyltransferase 2 family, polypeptide B28 | *UGT2B28* | 11.3 | 0.000166 | 0.0032287 |
| 162 | NM_174896.2 | Chromosome 1 open reading frame 162 | *C1ORF162* | 11.3 | 4.93E-08 | 2.30E-05 |
| 163 | NM_003122.2 | Serine peptidase inhibitor, Kazal type 1 | *SPINK1* | 11.1 | 0.0025112 | 0.0185761 |
| 164 | NM_004877.1 | Glia maturation factor, gamma | *GMFG* | 11.1 | 7.00E-09 | 6.48E-06 |
| 165 | NM_005565.3 | Lymphocyte cytosolic protein 2 | *LCP2* | 11.1 | 2.75E-08 | 1.63E-05 |
| 166 | NM_000940.1 | Paraoxonase 3 | *PON3* | 10.9 | 0.0003117 | 0.004716 |
| 167 | NM_020980.2 | Aquaporin 9 | *AQP9* | 10.9 | 9.80E-05 | 0.0023276 |
| 168 | NM_013322.2 | Sorting nexin 10 | *SNX10* | 10.8 | 0.00042 | 0.0057217 |
| 169 | NM_080912.1 | Asialoglycoprotein receptor 2 , transcript variant H2' | *ASGR2* | 10.7 | 1.13E-05 | 0.0006215 |
| 170 | NM_021232.1 | Proline dehydrogenase 2 | *PRODH2* | 10.7 | 1.18E-05 | 0.0006392 |
| 171 | NM_000784.2 | Cytochrome P450, family 27, subfamily A, polypeptide 1 , nuclear gene encoding mitochondrial protein | *CYP27A1* | 10.7 | 6.81E-06 | 0.0004534 |
| 172 | NM_000186.2 | Complement factor H , transcript variant 1 | *CFH* | 10.7 | 2.81E-06 | 0.0002901 |
| 173 | NM_001565.1 | Chemokine ligand 10 | *CXCL10* | 10.6 | 4.91E-06 | 0.0003717 |
| 174 | NM_198998.1 | Aquaporin 12A | *AQP12A* | 10.6 | 2.81E-06 | 0.0002901 |
| 175 | NM_000612.2 | Insulin-like growth factor 2 | *IGF2* | 10.5 | 0.0031684 | 0.0215694 |
| 176 | NM_002150.2 | 4-hydroxyphenylpyruvate dioxygenase | *HPD* | 10.5 | 0.0009803 | 0.0099833 |
| 177 | NM_003725.2 | Hydroxysteroid dehydrogenase 6 | *HSD17B6* | 10.5 | 0.0002636 | 0.0042754 |
| 178 | NM_153699.1 | Glutathione S-transferase A5 | *GSTA5* | 10.4 | 0.0010507 | 0.0104996 |
| 179 | AK130022 | Cdna FLJ26512 fis, clone KDN07513 | *HS.282795* | 10.4 | 6.64E-06 | 0.0004454 |
| 180 | NM_004847.2 | Allograft inflammatory factor 1 , transcript variant 2 | *AIF1* | 10.4 | 2.95E-08 | 1.68E-05 |
| 181 | NM_000587.2 | Complement component 7 | *C7* | 10.3 | 0.0005404 | 0.0068108 |
| 182 | NM_000597.2 | Insulin-like growth factor binding protein 2, 36kda | *IGFBP2* | 10.3 | 0.0006975 | 0.0080496 |
| 183 | NM_001629.2 | Arachidonate 5-lipoxygenase-activating protein | *ALOX5AP* | 10.2 | 2.35E-07 | 5.96E-05 |
| 184 | NM_020998.2 | Macrophage stimulating 1 | *MST1* | 10.2 | 1.69E-05 | 0.0007773 |
| 185 | NM_005807.2 | Proteoglycan 4 | *PRG4* | 10.2 | 0.000115 | 0.0025957 |
| 186 | NM_003645.2 | Solute carrier family 27 , member 2 | *SLC27A2* | 10.2 | 8.10E-06 | 0.0004983 |
| 187 | NM_203416.1 | CD163 antigen , transcript variant 2 | *CD163* | 10.1 | 1.38E-06 | 0.0001794 |
| 188 | NM_000062.2 | Serpin peptidase inhibitor, clade G , member 1, , transcript variant 1 | *SERPING1* | 10.0 | 1.36E-06 | 0.0001776 |
| 189 | NM_002155.3 | Heat shock 70kda protein 6 | *HSPA6* | 9.9 | 2.38E-07 | 5.96E-05 |
| 190 | NM_032049.1 | Angiotensin II receptor, type 1 , transcript variant 5 | *AGTR1* | 9.8 | 0.0001234 | 0.0027037 |
| 191 | NM_005461.3 | V-maf musculoaponeurotic fibrosarcoma oncogene homolog B | *MAFB* | 9.8 | 2.47E-07 | 6.03E-05 |
| 192 | NM_002416.1 | Chemokine ligand 9 | *CXCL9* | 9.8 | 9.52E-05 | 0.0022932 |
| 193 | NM_005666.2 | Complement factor H-related 2 | *CFHR2* | 9.8 | 0.0001744 | 0.0032988 |
| 194 | XM_944822.1 | PREDICTED: similar to HLA class II histocompatibility antigen, DRB1-9 beta chain precursor , transcript variant 2 | *LOC649143* | 9.8 | 5.34E-05 | 0.0016032 |
| 195 | NM_001134.1 | Alpha-fetoprotein | *AFP* | 9.7 | 0.004 | 0.026924 |
| 196 | NM_001875.2 | Carbamoyl-phosphate synthetase 1, mitochondrial | *CPS1* | 9.6 | 0.0007076 | 0.0081116 |
| 197 | NM_182758.1 | WD repeat domain 72 | *WDR72* | 9.3 | 0.008 | 0.0390932 |
| 198 | NM_153206.1 | Adhesion molecule, interacts with CXADR antigen 1 | *AMICA1* | 9.3 | 3.40E-07 | 7.66E-05 |
| 199 | NM_000898.3 | Monoamine oxidase B , nuclear gene encoding mitochondrial protein | *MAOB* | 9.2 | 1.21E-05 | 0.0006477 |
| 200 | NM_004497.2 | Forkhead box A3 | *FOXA3* | 9.1 | 0.0003459 | 0.0050715 |
| 201 | NM_013261.2 | Peroxisome proliferative activated receptor, gamma, coactivator 1, alpha | *PPARGC1A* | 9.1 | 3.69E-06 | 0.0003239 |
| 202 | NM_000204.1 | Complement factor I | *CFI* | 9.0 | 5.33E-08 | 2.39E-05 |
| 203 | NM_002763.3 | Prospero-related homeobox 1 | *PROX1* | 9.0 | 1.32E-05 | 0.0006779 |
| 204 | NM_006657.2 | Formiminotransferase cyclodeaminase , transcript variant B | *FTCD* | 8.9 | 0.0001894 | 0.0034771 |
| 205 | X00437 | Human mrna for T-cell specific protein | *HS.534427* | 8.8 | 1.33E-05 | 0.0006783 |
| 206 | NM_005835.1 | Solute carrier family 17 , member 2 | *SLC17A2* | 8.8 | 0.0003075 | 0.0046764 |
| 207 | NM_021969.1 | Nuclear receptor subfamily 0, group B, member 2 | *NR0B2* | 8.8 | 3.54E-05 | 0.0012491 |
| 208 | NM_003226.2 | Trefoil factor 3 | *TFF3* | 8.8 | 0.0016435 | 0.0139506 |
| 209 | NM_032866.3 | Cingulin-like 1 | *CGNL1* | 8.8 | 1.52E-05 | 0.0007316 |
| 210 | NM_182983.1 | Hepsin , transcript variant 1 | *HPN* | 8.6 | 1.14E-06 | 0.0001614 |
| 211 | NM_004120.3 | Guanylate binding protein 2, interferon-inducible | *GBP2* | 8.5 | 1.58E-05 | 0.0007491 |
| 212 | NM_014430.1 | Cell death-inducing DFFA-like effector b | *CIDEB* | 8.5 | 3.44E-06 | 0.0003143 |
| 213 | NM_018242.2 | Hypothetical protein FLJ10847 | *FLJ10847* | 8.4 | 1.05E-05 | 0.0005968 |
| 214 | NM_000377.1 | Wiskott-Aldrich syndrome | *WAS* | 8.4 | 5.96E-08 | 2.39E-05 |
| 215 | NM_001385.1 | Dihydropyrimidinase | *DPYS* | 8.4 | 0.0002429 | 0.0040882 |
| 216 | NM_000954.5 | Prostaglandin D2 synthase 21kda | *PTGDS* | 8.3 | 0.0019992 | 0.0159806 |
| 217 | NM_001713.1 | Betaine-homocysteine methyltransferase | *BHMT* | 8.3 | 0.0001243 | 0.0027114 |
| 218 | NM_001010919.1 | Hypothetical protein LOC441168 | *LOC441168* | 8.3 | 4.56E-05 | 0.0014451 |
| 219 | NM_004684.2 | SPARC-like 1 | *SPARCL1* | 8.3 | 3.05E-06 | 0.0002965 |
| 220 | NM_001461.1 | Flavin containing monooxygenase 5 | *FMO5* | 8.2 | 2.13E-05 | 0.0009128 |
| 221 | NM_002928.2 | Regulator of G-protein signalling 16 | *RGS16* | 8.2 | 6.15E-06 | 0.0004285 |
| 222 | NM_138326.1 | Aminocarboxymuconate semialdehyde decarboxylase | *ACMSD* | 8.0 | 3.66E-05 | 0.0012741 |
| 223 | NM_006209.2 | Ectonucleotide pyrophosphatase/phosphodiesterase 2 | *ENPP2* | 8.0 | 6.40E-05 | 0.001796 |
| 224 | NM_013364.2 | Paraneoplastic antigen MA3 | *PNMA3* | 8.0 | 0.0003507 | 0.0051137 |
| 225 | NM_000771.2 | Cytochrome P450, family 2, subfamily C, polypeptide 9 | *CYP2C9* | 7.9 | 0.003115 | 0.021339 |
| 226 | NM_178562.2 | Tetraspanin 33 | *TSPAN33* | 7.9 | 1.30E-06 | 0.0001734 |
| 227 | NM_002163.2 | Interferon regulatory factor 8 | *IRF8* | 7.8 | 5.84E-06 | 0.000409 |
| 228 | NM_015136.2 | Stabilin 1 | *STAB1* | 7.8 | 4.30E-08 | 2.20E-05 |
| 229 | NM_000397.2 | Cytochrome b-245, beta polypeptide | *CYBB* | 7.8 | 3.14E-07 | 7.12E-05 |
| 230 | NM_012190.2 | Aldehyde dehydrogenase 1 family, member L1 | *ALDH1L1* | 7.8 | 0.0006232 | 0.0074925 |
| 231 | XM_937586.1 | PREDICTED: hypothetical protein FLJ21438 | *FLJ21438* | 7.7 | 2.19E-07 | 5.78E-05 |
| 232 | NM_014333.2 | Immunoglobulin superfamily, member 4 | *IGSF4* | 7.7 | 8.10E-07 | 0.0001295 |
| 233 | NM_016518.2 | Pipecolic acid oxidase | *PIPOX* | 7.6 | 9.20E-05 | 0.0022357 |
| 234 | NM_173596.1 | Solute carrier family 39 , member 5 | *SLC39A5* | 7.6 | 0.0008214 | 0.0089035 |
| 235 | NM_152672.3 | Organic solute transporter alpha | *OSTALPHA* | 7.5 | 0.0004905 | 0.0063403 |
| 236 | NM_000142.2 | Fibroblast growth factor receptor 3 , transcript variant 1 | *FGFR3* | 7.5 | 0.0001749 | 0.0033011 |
| 237 | NM_000504.2 | Coagulation factor X | *F10* | 7.5 | 0.0002148 | 0.0037889 |
| 238 | NM_001066.2 | Tumor necrosis factor receptor superfamily, member 1B | *TNFRSF1B* | 7.5 | 9.25E-07 | 0.0001404 |
| 239 | NM_005335.3 | Hematopoietic cell-specific Lyn substrate 1 | *HCLS1* | 7.5 | 2.76E-05 | 0.001074 |
| 240 | NM_005460.2 | Synuclein, alpha interacting protein | *SNCAIP* | 7.5 | 0.0004704 | 0.0061785 |
| 241 | NM_032607.1 | Camp responsive element binding protein 3-like 3 | *CREB3L3* | 7.4 | 9.15E-05 | 0.0022323 |
| 242 | NM_014266.3 | Hematopoietic cell signal transducer , transcript variant 1 | *HCST* | 7.4 | 1.69E-05 | 0.0007773 |
| 243 | NM_000151.1 | Glucose-6-phosphatase, catalytic | *G6PC* | 7.4 | 0.0005687 | 0.0070767 |
| 244 | NM_175571.2 | Gtpase, IMAP family member 8 | *GIMAP8* | 7.3 | 1.70E-07 | 4.92E-05 |
| 245 | NM_006843.2 | Serine dehydratase | *SDS* | 7.3 | 0.0004393 | 0.0059067 |
| 246 | NM_006705.2 | Growth arrest and DNA-damage-inducible, gamma | *GADD45G* | 7.2 | 6.48E-05 | 0.0018022 |
| 247 | NM_002965.2 | S100 calcium binding protein A9 | *S100A9* | 7.2 | 0.0002136 | 0.0037692 |
| 248 | NM_021021.2 | Syntrophin, beta 1 | *SNTB1* | 7.2 | 0.0006631 | 0.0077816 |
| 249 | NM_173452.1 | Ficolin 3 , transcript variant 2 | *FCN3* | 7.2 | 0.0021182 | 0.0165971 |
| 250 | NM_152309.2 | Phosphoinositide-3-kinase adaptor protein 1 | *PIK3AP1* | 7.2 | 8.03E-07 | 0.0001294 |
| 251 | NM_030769.1 | N-acetylneuraminate pyruvate lyase | *NPL* | 7.2 | 3.23E-06 | 0.0003002 |
| 252 | NM_031481.1 | Solute carrier family 25 , member 18 | *SLC25A18* | 7.1 | 0.000118 | 0.0026389 |
| 253 | NM_000353.1 | Tyrosine aminotransferase , nuclear gene encoding mitochondrial protein | *TAT* | 7.1 | 0.0010933 | 0.0107849 |
| 254 | NM_003196.1 | Transcription elongation factor A , 3 | *TCEA3* | 7.1 | 0.0006182 | 0.0074529 |
| 255 | NM_024758.3 | Agmatine ureohydrolase | *AGMAT* | 7.0 | 4.27E-06 | 0.0003463 |
| 256 | NM_001666.2 | Rho gtpase activating protein 4 | *ARHGAP4* | 7.0 | 5.33E-08 | 2.39E-05 |
| 257 | NM_000531.3 | Ornithine carbamoyltransferase | *OTC* | 6.9 | 0.0010502 | 0.0104987 |
| 258 | NM_002124.1 | Major histocompatibility complex, class II, DR beta 1 | *HLA-DRB1* | 6.8 | 0.0015398 | 0.0134233 |
| 259 | NM_152435.1 | Amidohydrolase domain containing 1 | *AMDHD1* | 6.8 | 1.94E-05 | 0.000854 |
| 260 | NM_032562.2 | Phospholipase A2, group XIIB | *PLA2G12B* | 6.7 | 6.86E-05 | 0.0018579 |
| 261 | NM_001979.4 | Epoxide hydrolase 2, cytoplasmic | *EPHX2* | 6.6 | 6.95E-05 | 0.0018691 |
| 262 | NM_000790.2 | Dopa decarboxylase | *DDC* | 6.6 | 0.0002997 | 0.0046041 |
| 263 | NM_003810.2 | Tumor necrosis factor superfamily, member 10 | *TNFSF10* | 6.6 | 0.0014615 | 0.0130188 |
| 264 | NM_173217.1 | ST6 beta-galactosamide alpha-2,6-sialyltranferase 1 , transcript variant 3 | *ST6GAL1* | 6.5 | 1.02E-06 | 0.000151 |
| 265 | NM_000313.1 | Protein S | *PROS1* | 6.5 | 1.04E-05 | 0.0005968 |
| 266 | NM_002474.1 | Myosin, heavy polypeptide 11, smooth muscle , transcript variant SM1 | *MYH11* | 6.5 | 2.69E-05 | 0.0010573 |
| 267 | NM_006332.3 | Interferon, gamma-inducible protein 30 | *IFI30* | 6.5 | 1.93E-07 | 5.43E-05 |
| 268 | NM_000163.2 | Growth hormone receptor | *GHR* | 6.4 | 0.005 | 0.0307359 |
| 269 | NM_152637.1 | Methyltransferase like 7B | *METTL7B* | 6.4 | 3.13E-06 | 0.0002997 |
| 270 | NM_001710.4 | Complement factor B | *CFB* | 6.3 | 0.0002624 | 0.0042674 |
| 271 | NM_006748.1 | Src-like-adaptor | *SLA* | 6.3 | 2.34E-07 | 5.96E-05 |
| 272 | NM_000242.1 | Mannose-binding lectin 2, soluble | *MBL2* | 6.2 | 0.0002505 | 0.004167 |
| 273 | NM_006944.2 | Secreted phosphoprotein 2, 24kda | *SPP2* | 6.2 | 0.0037516 | 0.0241061 |
| 274 | NM_006144.2 | Granzyme A | *GZMA* | 6.2 | 0.0001117 | 0.0025545 |
| 275 | NM_002084.2 | Glutathione peroxidase 3 | *GPX3* | 6.2 | 0.0010153 | 0.0102629 |
| 276 | NM_005615.2 | Ribonuclease, rnase A family, k6 | *RNASE6* | 6.2 | 2.02E-07 | 5.54E-05 |
| 277 | NM_000312.1 | Protein C | *PROC* | 6.2 | 0.0002776 | 0.0043887 |
| 278 | NM_018404.1 | Centaurin, alpha 2 | *CENTA2* | 6.1 | 3.90E-07 | 8.44E-05 |
| 279 | NM_000896.1 | Cytochrome P450, family 4, subfamily F, polypeptide 3 | *CYP4F3* | 6.1 | 0.0003772 | 0.0053223 |
| 280 | NM_153236.3 | Gtpase, IMAP family member 7 | *GIMAP7* | 6.1 | 2.74E-07 | 6.43E-05 |
| 281 | NM_005258.2 | GTP cyclohydrolase I feedback regulator | *GCHFR* | 6.1 | 0.0005096 | 0.0065163 |
| 282 | NM_014571.2 | Hairy/enhancer-of-split related with YRPW motif-like | *HEYL* | 6.1 | 4.52E-06 | 0.0003568 |
| 283 | NM_033086.1 | FYVE, rhogef and PH domain containing 3 | *FGD3* | 6.0 | 6.85E-06 | 0.000455 |
| 284 | NM_006210.1 | Paternally expressed 3 | *PEG3* | 6.0 | 0.0038112 | 0.0243325 |
| 285 | NM_001767.2 | CD2 antigen , sheep red blood cell receptor | *CD2* | 6.0 | 6.88E-06 | 0.0004558 |
| 286 | NM_000478.2 | Alkaline phosphatase, liver/bone/kidney | *ALPL* | 5.9 | 0.0004868 | 0.0063117 |
| 287 | NM_005084.2 | Phospholipase A2, group VII | *PLA2G7* | 5.9 | 0.0001689 | 0.0032582 |
| 288 | NM_001638.2 | Apolipoprotein F | *APOF* | 5.9 | 0.0029984 | 0.0208054 |
| 289 | NM_000300.2 | Phospholipase A2, group IIA | *PLA2G2A* | 5.8 | 0.006 | 0.0322845 |
| 290 | NM_002432.1 | Myeloid cell nuclear differentiation antigen | *MNDA* | 5.8 | 9.97E-08 | 3.34E-05 |
| 291 | NM_001308.1 | Carboxypeptidase N, polypeptide 1, 50kd | *CPN1* | 5.8 | 0.0002932 | 0.0045441 |
| 292 | NM_024660.1 | U2 small nuclear RNA auxiliary factor 1-like 4 | *U2AF1L4* | 5.8 | 4.37E-07 | 8.85E-05 |
| 293 | NM_003120.1 | Spleen focus forming virus proviral integration oncogene spi1 | *SPI1* | 5.8 | 6.01E-07 | 0.0001098 |
| 294 | NM_001778.2 | CD48 antigen | *CD48* | 5.8 | 1.53E-05 | 0.0007316 |
| 295 | NM_002405.2 | Manic fringe homolog | *MFNG* | 5.8 | 4.82E-05 | 0.0014937 |
| 296 | NM_004946.1 | Dedicator of cytokinesis 2 | *DOCK2* | 5.8 | 4.70E-06 | 0.0003615 |
| 297 | NM_005623.2 | Chemokine ligand 8 | *CCL8* | 5.8 | 0.000373 | 0.0052884 |
| 298 | NM_001185.2 | Alpha-2-glycoprotein 1, zinc | *AZGP1* | 5.8 | 0.0012463 | 0.0117859 |
| 299 | NM_020796.2 | Sema domain, transmembrane domain , and cytoplasmic domain, 6A | *SEMA6A* | 5.7 | 2.14E-07 | 5.78E-05 |
| 300 | NM_018687.3 | Hepatocellular carcinoma-associated gene TD26 | *LOC55908* | 5.7 | 0.0003588 | 0.0051918 |
| 301 | NM_005601.3 | Natural killer cell group 7 sequence | *NKG7* | 5.7 | 0.0001871 | 0.0034506 |
| 302 | NM_000765.2 | Cytochrome P450, family 3, subfamily A, polypeptide 7 | *CYP3A7* | 5.7 | 0.0033727 | 0.0225485 |
| 303 | NM_003708.2 | Retinol dehydrogenase 16 | *RDH16* | 5.7 | 0.000978 | 0.0099637 |
| 304 | NM_003621.1 | PTPRF interacting protein, binding protein 2 | *PPFIBP2* | 5.7 | 1.45E-07 | 4.46E-05 |
| 305 | NM_005123.1 | Nuclear receptor subfamily 1, group H, member 4 | *NR1H4* | 5.6 | 1.24E-06 | 0.0001702 |
| 306 | NM_001093.2 | Acetyl-Coenzyme A carboxylase beta | *ACACB* | 5.6 | 7.23E-05 | 0.0019284 |
| 307 | NM_000429.2 | Methionine adenosyltransferase I, alpha | *MAT1A* | 5.6 | 0.0003178 | 0.0047831 |
| 308 | NM_006633.1 | IQ motif containing gtpase activating protein 2 | *IQGAP2* | 5.6 | 4.44E-07 | 8.85E-05 |
| 309 | NM_006864.1 | Leukocyte immunoglobulin-like receptor, subfamily B , member 3 | *LILRB3* | 5.5 | 2.83E-06 | 0.0002901 |
| 310 | XM_940876.1 | PREDICTED: similar to HLA class II histocompatibility antigen, DQ alpha chain precursor , transcript variant 1 | *LOC650946* | 5.5 | 0.0016717 | 0.0140852 |
| 311 | NM_000507.2 | Fructose-1,6-bisphosphatase 1 | *FBP1* | 5.5 | 0.0009709 | 0.0099261 |
| 312 | NM_016327.2 | Ureidopropionase, beta | *UPB1* | 5.5 | 0.0024732 | 0.0183855 |
| 313 | NM_025243.2 | Solute carrier family 19, member 3 | *SLC19A3* | 5.5 | 0.0003059 | 0.0046664 |
| 314 | NM_001159.3 | Aldehyde oxidase 1 | *AOX1* | 5.5 | 0.0029016 | 0.020337 |
| 315 | NM_017614.3 | Betaine-homocysteine methyltransferase 2 | *BHMT2* | 5.4 | 0.0005603 | 0.0070003 |
| 316 | NM_005360.3 | V-maf musculoaponeurotic fibrosarcoma oncogene homolog , transcript variant 1 | *MAF* | 5.4 | 1.75E-07 | 5.01E-05 |
| 317 | NM_002885.1 | RAP1, gtpase activating protein 1 | *RAP1GA1* | 5.4 | 0.0011629 | 0.011258 |
| 318 | NM_002302.2 | Leukocyte cell-derived chemotaxin 2 | *LECT2* | 5.4 | 0.0019449 | 0.0156967 |
| 319 | NM_020995.3 | Haptoglobin-related protein | *HPR* | 5.4 | 0.0008582 | 0.0091467 |
| 320 | NM_003500.2 | Acyl-Coenzyme A oxidase 2, branched chain | *ACOX2* | 5.3 | 0.005 | 0.0274059 |
| 321 | XM_945034.1 | PREDICTED: carboxylesterase 1 , transcript variant 5 | *CES1* | 5.3 | 0.0003382 | 0.004995 |
| 322 | NM_001774.1 | CD37 antigen | *CD37* | 5.3 | 2.91E-06 | 0.0002918 |
| 323 | NM_004271.3 | Lymphocyte antigen 86 | *LY86* | 5.3 | 1.15E-07 | 3.69E-05 |
| 324 | NM_002729.2 | Hematopoietically expressed homeobox | *HHEX* | 5.3 | 5.03E-06 | 0.0003774 |
| 325 | NM_016323.1 | Hect domain and RLD 5 | *HERC5* | 5.3 | 7.49E-06 | 0.0004677 |
| 326 | NM_000772.1 | Cytochrome P450, family 2, subfamily C, polypeptide 18 | *CYP2C18* | 5.2 | 0.0010197 | 0.0102995 |
| 327 | NM_000187.1 | Homogentisate 1,2-dioxygenase | *HGD* | 5.2 | 2.41E-05 | 0.000986 |
| 328 | NM_001251.1 | CD68 antigen | *CD68* | 5.2 | 2.99E-05 | 0.0011295 |
| 329 | NM_000160.1 | Glucagon receptor | *GCGR* | 5.2 | 0.007 | 0.0346732 |
| 330 | NM_138280.3 | Citrate lyase beta like , transcript variant 1 | *CLYBL* | 5.1 | 1.30E-06 | 0.0001734 |
| 331 | NM_138393.1 | Receptor accessory protein 6 | *REEP6* | 5.1 | 0.0008984 | 0.0094256 |
| 332 | NM_002119.3 | Major histocompatibility complex, class II, DO alpha | *HLA-DOA* | 5.1 | 8.69E-05 | 0.0021593 |
| 333 | NM_198053.1 | CD3Z antigen, zeta polypeptide , transcript variant 1 | *CD3Z* | 5.1 | 3.26E-05 | 0.0011845 |
| 334 | BQ438671 | AGENCOURT_7908292 NIH_MGC_82 cdna clone IMAGE:6102595 5, mrna sequence | *HS.583806* | 5.1 | 2.01E-05 | 0.0008751 |
| 335 | NM_000892.2 | Kallikrein B, plasma 1 | *KLKB1* | 5.1 | 0.0004564 | 0.0060617 |
| 336 | NM_002104.2 | Granzyme K | *GZMK* | 5.1 | 7.54E-05 | 0.0019723 |
| 337 | NM_015900.1 | Phospholipase A1 member A | *PLA1A* | 5.1 | 0.0009099 | 0.0095101 |
| 338 | NM_021784.3 | Forkhead box A2 , transcript variant 1 | *FOXA2* | 5.1 | 0.0013503 | 0.0124007 |
| 339 | NM_153281.1 | Hyaluronoglucosaminidase 1 , transcript variant 8 | *HYAL1* | 5.1 | 0.0001154 | 0.0026024 |
| 340 | NM_001086.2 | Arylacetamide deacetylase | *AADAC* | 5.0 | 0.0013602 | 0.0124701 |
| 341 | NM_018490.1 | Leucine-rich repeat-containing G protein-coupled receptor 4 | *LGR4* | 5.0 | 2.26E-06 | 0.0002502 |
| 342 | NM_013385.2 | Pleckstrin homology, Sec7 and coiled-coil domains 4 | *PSCD4* | 5.0 | 2.69E-06 | 0.0002868 |
| 343 | NM_001216.1 | Carbonic anhydrase IX | *CA9* | 5.0 | 0.0028407 | 0.0200826 |
| 344 | BG545303 | 602572519F1 NIH_MGC_77 cdna clone IMAGE:4700548 5, mrna sequence | *HS.137274* | 5.0 | 0.0001243 | 0.0027114 |
| 345 | BC038512 | Cdna clone IMAGE:5262734 | *HS.296031* | 5.0 | 2.29E-05 | 0.0009553 |
| 346 | NM_016186.1 | Serpin peptidase inhibitor, clade A , member 10 | *SERPINA10* | 5.0 | 0.0001218 | 0.0026936 |
| 347 | NM_000047.1 | Arylsulfatase E | *ARSE* | 5.0 | 4.23E-06 | 0.0003463 |
| 348 | NM_000442.2 | Platelet/endothelial cell adhesion molecule | *PECAM1* | 5.0 | 2.21E-07 | 5.78E-05 |
| 349 | NM_001646.1 | Apolipoprotein C-IV | *APOC4* | 5.0 | 0.004215 | 0.0259835 |
| 350 | NM_020247.3 | Chaperone, ABC1 activity of bc1 complex like | *CABC1* | 5.0 | 1.96E-06 | 0.0002289 |
| 351 | NM_002985.2 | Chemokine ligand 5 | *CCL5* | 5.0 | 0.0002056 | 0.0036849 |
| 352 | NM_005161.2 | Angiotensin II receptor-like 1 | *AGTRL1* | 5.0 | 5.44E-05 | 0.0016165 |
| 353 | NM_018384.3 | Gtpase, IMAP family member 5 | *GIMAP5* | 4.9 | 4.74E-06 | 0.0003619 |
| 354 | NM_002030.3 | Formyl peptide receptor-like 2 | *FPRL2* | 4.9 | 5.77E-07 | 0.0001076 |
| 355 | NM_000803.2 | Folate receptor 2 | *FOLR2* | 4.9 | 3.70E-05 | 0.0012783 |
| 356 | NM_130759.2 | Gtpase, IMAP family member 1 | *GIMAP1* | 4.9 | 2.11E-06 | 0.0002381 |
| 357 | NM_004951.3 | Epstein-Barr virus induced gene 2 | *EBI2* | 4.9 | 6.87E-07 | 0.0001164 |
| 358 | NM_002029.3 | Formyl peptide receptor 1 | *FPR1* | 4.9 | 1.30E-05 | 0.0006685 |
| 359 | NM_153609.2 | Transmembrane protease, serine 6 | *TMPRSS6* | 4.9 | 0.0002015 | 0.0036335 |
| 360 | NM_000131.2 | Coagulation factor VII , transcript variant 1 | *F7* | 4.8 | 0.0001348 | 0.0028325 |
| 361 | NM_000856.2 | Guanylate cyclase 1, soluble, alpha 3 | *GUCY1A3* | 4.8 | 0.0005585 | 0.0069853 |
| 362 | NM_001133.2 | Afamin | *AFM* | 4.8 | 0.005 | 0.0271484 |
| 363 | NM_004079.3 | Cathepsin S | *CTSS* | 4.8 | 9.79E-06 | 0.0005693 |
| 364 | NM_014234.3 | Hydroxysteroid dehydrogenase 8 | *HSD17B8* | 4.8 | 1.62E-05 | 0.0007609 |
| 365 | NM_005989.2 | Aldo-keto reductase family 1, member D1 | *AKR1D1* | 4.8 | 0.0030312 | 0.0209412 |
| 366 | NM_014270.3 | Solute carrier family 7 , member 9 | *SLC7A9* | 4.7 | 0.0003067 | 0.0046709 |
| 367 | NM_205860.1 | Nuclear receptor subfamily 5, group A, member 2 , transcript variant 1 | *NR5A2* | 4.7 | 1.28E-05 | 0.0006634 |
| 368 | NM_174977.2 | SEC14-like 4 | *SEC14L4* | 4.7 | 7.83E-05 | 0.0020111 |
| 369 | NM_006059.2 | Laminin, gamma 3 | *LAMC3* | 4.7 | 0.0007159 | 0.0081663 |
| 370 | NM_014880.3 | CD302 antigen | *CD302* | 4.7 | 4.31E-05 | 0.0014233 |
| 371 | NM_032962.2 | Chemokine ligand 14 , transcript variant 2 | *CCL14* | 4.7 | 0.0003477 | 0.005089 |
| 372 | NM_033054.1 | Myosin IG | *MYO1G* | 4.7 | 1.10E-05 | 0.0006128 |
| 373 | NM_020142.3 | NADH:ubiquinone oxidoreductase MLRQ subunit homolog | *LOC56901* | 4.7 | 0.0039553 | 0.0249436 |
| 374 | NM_002123.2 | Major histocompatibility complex, class II, DQ beta 1 | *HLA-DQB1* | 4.7 | 0.0023787 | 0.0179644 |
| 375 | NM_005693.1 | Nuclear receptor subfamily 1, group H, member 3 | *NR1H3* | 4.7 | 2.74E-06 | 0.000289 |
| 376 | NM_207117.2 | Chromosome 14 open reading frame 68 | *C14ORF68* | 4.7 | 0.0017682 | 0.014712 |
| 377 | NM_018593.3 | Solute carrier family 16 , member 10 | *SLC16A10* | 4.6 | 1.71E-06 | 0.000206 |
| 378 | NM_014694.2 | ADAMTS-like 2 | *ADAMTSL2* | 4.6 | 0.000419 | 0.005714 |
| 379 | NM_020384.2 | Claudin 2 | *CLDN2* | 4.6 | 0.0025547 | 0.0187354 |
| 380 | NM_014585.3 | Solute carrier family 40 , member 1 | *SLC40A1* | 4.6 | 3.44E-07 | 7.67E-05 |
| 381 | NM_005045.2 | Reelin , transcript variant 1 | *RELN* | 4.6 | 0.0010539 | 0.010515 |
| 382 | NM_016533.4 | Ninjurin 2 | *NINJ2* | 4.5 | 3.83E-07 | 8.39E-05 |
| 383 | NM_021187.2 | Cytochrome P450, family 4, subfamily F, polypeptide 11 | *CYP4F11* | 4.5 | 0.0036767 | 0.0238037 |
| 384 | NM_052941.2 | Guanylate binding protein 4 | *GBP4* | 4.5 | 0.0016533 | 0.0139924 |
| 385 | NM_016582.1 | Solute carrier family 15, member 3 | *SLC15A3* | 4.5 | 8.11E-05 | 0.0020606 |
| 386 | NM_000443.2 | ATP-binding cassette, sub-family B , member 4 , transcript variant A | *ABCB4* | 4.5 | 6.86E-05 | 0.0018579 |
| 387 | NM_003227.2 | Transferrin receptor 2 | *TFR2* | 4.5 | 0.0004669 | 0.0061457 |
| 388 | NM_023944.1 | Cytochrome P450, family 4, subfamily F, polypeptide 12 | *CYP4F12* | 4.5 | 0.0003228 | 0.0048262 |
| 389 | NM_183240.1 | Transmembrane protein 37 | *TMEM37* | 4.5 | 1.58E-07 | 4.68E-05 |
| 390 | NM_003467.2 | Chemokine receptor 4 , transcript variant 2 | *CXCR4* | 4.5 | 0.0007706 | 0.0085518 |
| 391 | NM_199335.2 | FYN binding protein , transcript variant 2 | *FYB* | 4.4 | 0.0001564 | 0.0031048 |
| 392 | NM_170726.1 | Aldehyde dehydrogenase 4 family, member A1 , nuclear gene encoding mitochondrial protein, transcript variant p5cdhs | *ALDH4A1* | 4.4 | 0.0001464 | 0.0029916 |
| 393 | NM_002661.1 | Phospholipase C, gamma 2 | *PLCG2* | 4.4 | 1.37E-05 | 0.000692 |
| 394 | NM_002110.2 | Hemopoietic cell kinase | *HCK* | 4.4 | 7.83E-07 | 0.0001286 |
| 395 | NM_025202.2 | EF-hand domain family, member D1 | *EFHD1* | 4.4 | 0.0004624 | 0.0061075 |
| 396 | NM_014573.1 | Transmembrane protein 97 | *TMEM97* | 4.4 | 0.0019488 | 0.0157139 |
| 397 | NM_000055.1 | Butyrylcholinesterase | *BCHE* | 4.4 | 0.0021927 | 0.0169746 |
| 398 | CR591180 | Full-length cdna clone CS0DM004YG21 of Fetal liver of | *HS.282703* | 4.4 | 0.0005441 | 0.0068412 |
| 399 | NM_006841.3 | Solute carrier family 38, member 3 | *SLC38A3* | 4.4 | 0.0013414 | 0.0123375 |
| 400 | NM_145202.3 | Proline-rich acidic protein 1 | *PRAP1* | 4.4 | 0.0006957 | 0.0080378 |
| 401 | NM_001801.2 | Cysteine dioxygenase, type I | *CDO1* | 4.4 | 0.0014986 | 0.0132146 |
| 402 | NM_002217.2 | Inter-alpha inhibitor H3 | *ITIH3* | 4.4 | 0.0002317 | 0.0039627 |
| 403 | NM_003627.4 | Solute carrier family 43, member 1 | *SLC43A1* | 4.4 | 4.64E-05 | 0.0014573 |
| 404 | NM_002612.2 | Pyruvate dehydrogenase kinase, isozyme 4 | *PDK4* | 4.3 | 0.0040613 | 0.0254084 |
| 405 | NM_001775.2 | CD38 antigen | *CD38* | 4.3 | 8.83E-05 | 0.0021831 |
| 406 | NM_001463.2 | Frizzled-related protein | *FRZB* | 4.3 | 9.16E-05 | 0.0022323 |
| 407 | BC035116 | Cdna clone IMAGE:5263177 | *HS.19339* | 4.3 | 7.35E-05 | 0.0019426 |
| 408 | NM_001024912.1 | Carcinoembryonic antigen-related cell adhesion molecule 1 , transcript variant 2 | *CEACAM1* | 4.3 | 0.000913 | 0.0095321 |
| 409 | NM_148918.1 | Serine hydroxymethyltransferase 1 , transcript variant 2 | *SHMT1* | 4.3 | 0.0003878 | 0.0054248 |
| 410 | NM_002983.1 | Chemokine ligand 3 | *CCL3* | 4.3 | 0.0006115 | 0.0073992 |
| 411 | NM_020439.2 | Calcium/calmodulin-dependent protein kinase IG | *CAMK1G* | 4.2 | 0.0023952 | 0.0180477 |
| 412 | NM_001001435.2 | Chemokine ligand 4-like 1 | *CCL4L1* | 4.2 | 0.0002403 | 0.0040571 |
| 413 | NM_001430.3 | Endothelial PAS domain protein 1 | *EPAS1* | 4.2 | 0.0024028 | 0.0180629 |
| 414 | NM_004004.3 | Gap junction protein, beta 2, 26kda | *GJB2* | 4.2 | 0.0002852 | 0.0044629 |
| 415 | NM_017424.2 | Cat eye syndrome chromosome region, candidate 1 , transcript variant 1 | *CECR1* | 4.2 | 1.63E-05 | 0.000761 |
| 416 | NM_020125.1 | SLAM family member 8 | *SLAMF8* | 4.2 | 0.0002551 | 0.0042176 |
| 417 | XM_375558 | PREDICTED: KIAA1881 , mrna | *HS.567652* | 4.2 | 0.000415 | 0.0056829 |
| 418 | NM_022437.2 | ATP-binding cassette, sub-family G , member 8 | *ABCG8* | 4.2 | 0.0002765 | 0.004379 |
| 419 | NM_012067.2 | Aldo-keto reductase family 7, member A3 | *AKR7A3* | 4.1 | 0.006 | 0.0333964 |
| 420 | NM_025195.2 | Tribbles homolog 1 | *TRIB1* | 4.1 | 9.16E-05 | 0.0022323 |
| 421 | NM_199327.1 | Sprouty homolog 1, antagonist of FGF signaling , transcript variant 2 | *SPRY1* | 4.1 | 0.0004218 | 0.0057431 |
| 422 | NM_012258.2 | Hairy/enhancer-of-split related with YRPW motif 1 | *HEY1* | 4.1 | 0.0006592 | 0.0077556 |
| 423 | NM_006573.3 | Tumor necrosis factor superfamily, member 13b | *TNFSF13B* | 4.1 | 0.0008358 | 0.0090143 |
| 424 | NM_024709.2 | Chromosome 1 open reading frame 115 | *C1ORF115* | 4.1 | 0.0013342 | 0.0123027 |
| 425 | NM_032496.1 | Rho gtpase activating protein 9 | *ARHGAP9* | 4.1 | 6.16E-06 | 0.0004285 |
| 426 | NM_001467.3 | Solute carrier family 37 , member 4 | *SLC37A4* | 4.1 | 6.67E-05 | 0.0018295 |
| 427 | NM_004288.3 | Pleckstrin homology, Sec7 and coiled-coil domains, binding protein | *PSCDBP* | 4.1 | 2.37E-05 | 0.0009755 |
| 428 | NM_004391.1 | Cytochrome P450, family 8, subfamily B, polypeptide 1 | *CYP8B1* | 4.1 | 0.007 | 0.0371094 |
| 429 | NM_001336.2 | Cathepsin Z | *CTSZ* | 4.0 | 1.43E-06 | 0.0001848 |
| 430 | NM_004117.2 | FK506 binding protein 5 | *FKBP5* | 4.0 | 1.88E-06 | 0.0002219 |
| 431 | NM_013314.2 | B-cell linker | *BLNK* | 4.0 | 2.04E-05 | 0.0008809 |
| 432 | NM_032041.1 | Neurocalcin delta | *NCALD* | 4.0 | 2.88E-06 | 0.0002901 |
| 433 | XM_926323.1 | PREDICTED: similar to Apolipoprotein precursor ) ) | *LOC653172* | 4.0 | 0.0009406 | 0.009702 |
| 434 | NM_005589.2 | Aldehyde dehydrogenase 6 family, member A1 , nuclear gene encoding mitochondrial protein | *ALDH6A1* | 4.0 | 0.0002585 | 0.0042351 |
| 435 | NM_145888.1 | Kallikrein 10 , transcript variant 2 | *KLK10* | -4.0 | 0.0016019 | 0.0137156 |
| 436 | NM_003633.1 | Ectodermal-neural cortex | *ENC1* | -4.0 | 2.86E-05 | 0.0011015 |
| 437 | NM_007026.1 | Dual specificity phosphatase 14 | *DUSP14* | -4.0 | 2.90E-05 | 0.0011107 |
| 438 | XM_932346.1 | PREDICTED: hypothetical protein LOC644743 | *LOC644743* | -4.0 | 0.0008402 | 0.0090325 |
| 439 | XM_166571.4 | PREDICTED: KIAA0363 protein, transcript variant 1 | *KIAA0363* | -4.0 | 4.50E-05 | 0.001442 |
| 440 | NM_004431.2 | EPH receptor A2 | *EPHA2* | -4.0 | 0.0025295 | 0.0186611 |
| 441 | NM_033512.2 | TSPY-like 5 | *TSPYL5* | -4.1 | 3.94E-07 | 8.44E-05 |
| 442 | NM_001197.3 | BCL2-interacting killer | *BIK* | -4.1 | 0.008 | 0.0392549 |
| 443 | NM_006404.3 | Protein C receptor, endothelial | *PROCR* | -4.1 | 3.95E-06 | 0.0003402 |
| 444 | NM_003486.5 | Solute carrier family 7 , member 5 | *SLC7A5* | -4.1 | 0.0001829 | 0.0034006 |
| 445 | NM_022450.2 | Rhomboid 5 homolog 1 | *RHBDF1* | -4.1 | 3.13E-07 | 7.12E-05 |
| 446 | NM_013246.2 | Cardiotrophin-like cytokine factor 1 | *CLCF1* | -4.1 | 0.0005018 | 0.0064513 |
| 447 | NM_001699.3 | AXL receptor tyrosine kinase , transcript variant 2 | *AXL* | -4.1 | 0.0003227 | 0.0048262 |
| 448 | NM_005860.1 | Follistatin-like 3 | *FSTL3* | -4.1 | 0.0024052 | 0.0180727 |
| 449 | NM_144569.3 | SPOC domain containing 1 | *SPOCD1* | -4.1 | 2.82E-05 | 0.0010892 |
| 450 | NM_024642.2 | UDP-N-acetyl-alpha-D-galactosamine:polypeptide N-acetylgalactosaminyltransferase 12 | *GALNT12* | -4.2 | 0.0006101 | 0.0073896 |
| 451 | NM_000599.2 | Insulin-like growth factor binding protein 5 | *IGFBP5* | -4.2 | 0.007 | 0.0347491 |
| 452 | NM_020826.1 | Synaptotagmin XIII | *SYT13* | -4.2 | 0.0027536 | 0.019695 |
| 453 | NM_031934.3 | RAB34, member RAS oncogene family | *RAB34* | -4.2 | 7.73E-05 | 0.0019996 |
| 454 | NR_001562.1 | Annexin A2 pseudogene 1 on chromosome 4. | *ANXA2P1* | -4.2 | 0.0002001 | 0.0036119 |
| 455 | NM_015187.1 | KIAA0746 protein | *KIAA0746* | -4.2 | 6.31E-06 | 0.0004381 |
| 456 | NM_003280.1 | Troponin C type 1 | *TNNC1* | -4.2 | 0.0001381 | 0.0028795 |
| 457 | NM_053056.1 | Cyclin D1 | *CCND1* | -4.2 | 0.0006951 | 0.0080347 |
| 458 | NM_002448.1 | Msh homeo box homolog 1 | *MSX1* | -4.3 | 0.0002584 | 0.0042351 |
| 459 | XM_942831.1 | PREDICTED: hypothetical LOC401074 | *LOC401074* | -4.3 | 2.66E-05 | 0.0010471 |
| 460 | NM_016639.1 | Tumor necrosis factor receptor superfamily, member 12A | *TNFRSF12A* | -4.3 | 4.70E-06 | 0.0003615 |
| 461 | NM_005928.1 | Milk fat globule-EGF factor 8 protein | *MFGE8* | -4.3 | 0.0003957 | 0.0054939 |
| 462 | NM_006779.2 | CDC42 effector protein 2 | *CDC42EP2* | -4.3 | 5.94E-08 | 2.39E-05 |
| 463 | NM_004696.1 | Solute carrier family 16 , member 4 | *SLC16A4* | -4.3 | 1.61E-06 | 0.0001978 |
| 464 | NM_005987.2 | Small proline-rich protein 1A | *SPRR1A* | -4.3 | 1.92E-05 | 0.0008497 |
| 465 | NM_002354.1 | Tumor-associated calcium signal transducer 1 | *TACSTD1* | -4.4 | 0.009 | 0.0406633 |
| 466 | CD640673 | AGENCOURT_14535501 NIH_MGC_191 cdna clone IMAGE:30415823 5, mrna sequence | *HS.543887* | -4.4 | 8.10E-09 | 6.56E-06 |
| 467 | NM_174911.3 | Family with sequence similarity 84, member B | *FAM84B* | -4.4 | 0.0006462 | 0.0076702 |
| 468 | NM_005270.2 | GLI-Kruppel family member GLI2 , transcript variant 4 | *GLI2* | -4.4 | 1.47E-05 | 0.0007231 |
| 469 | NM_001235.2 | Serpin peptidase inhibitor, clade H , member 1, | *SERPINH1* | -4.4 | 0.0014783 | 0.0131108 |
| 470 | NM_012232.2 | Polymerase I and transcript release factor | *PTRF* | -4.4 | 4.20E-05 | 0.0013901 |
| 471 | NM_000903.2 | NADH dehydrogenase, quinone 1 , transcript variant 1 | *NQO1* | -4.4 | 0.0006862 | 0.0079746 |
| 472 | NM_006039.2 | Mannose receptor, C type 2 | *MRC2* | -4.4 | 0.000364 | 0.0052275 |
| 473 | NM_019007.3 | Armadillo repeat containing, X-linked 6 , transcript variant 1 | *ARMCX6* | -4.5 | 2.77E-06 | 0.0002901 |
| 474 | NM_020873.3 | Leucine rich repeat neuronal 1 | *LRRN1* | -4.5 | 0.000792 | 0.0086822 |
| 475 | NM_025217.2 | UL16 binding protein 2 | *ULBP2* | -4.5 | 5.09E-06 | 0.0003791 |
| 476 | NM_020814.1 | Membrane-associated ring finger 4 | *04-Mar* | -4.5 | 0.0002876 | 0.0044836 |
| 477 | NM_006379.2 | Sema domain, immunoglobulin domain , short basic domain, secreted, 3C | *SEMA3C* | -4.5 | 0.0035699 | 0.02337 |
| 478 | NM_021978.2 | Suppression of tumorigenicity 14 | *ST14* | -4.5 | 0.0016175 | 0.0138025 |
| 479 | NM_000950.1 | Proline rich Gla 1 | *PRRG1* | -4.6 | 0.0005697 | 0.0070779 |
| 480 | NM_057164.2 | Collagen, type VI, alpha 3 , transcript variant 2 | *COL6A3* | -4.6 | 0.0020551 | 0.0162498 |
| 481 | XM_929387.1 | PREDICTED: similar to Ubiquitin-conjugating enzyme E2 H | *LOC646463* | -4.6 | 5.30E-06 | 0.0003821 |
| 482 | XM_926996.1 | PREDICTED: similar to Keratin, type I cytoskeletal 18 | *LOC340598* | -4.6 | 0.0009826 | 0.0099952 |
| 483 | NM_005279.2 | G protein-coupled receptor 1 | *GPR1* | -4.6 | 0.0039775 | 0.0250642 |
| 484 | NM_001002857.1 | Annexin A2 , transcript variant 2 | *ANXA2* | -4.6 | 8.00E-05 | 0.0020393 |
| 485 | NM_006516.1 | Solute carrier family 2 , member 1 | *SLC2A1* | -4.7 | 0.0007072 | 0.0081116 |
| 486 | NM_004370.4 | Collagen, type XII, alpha 1 , transcript variant long | *COL12A1* | -4.7 | 0.0007517 | 0.0084427 |
| 487 | NM_014241.2 | Protein tyrosine phosphatase-like , member a | *PTPLA* | -4.7 | 0.0011543 | 0.011213 |
| 488 | NM_014603.1 | Cerebellar degeneration-related protein 2-like | *CDR2L* | -4.8 | 4.27E-06 | 0.0003463 |
| 489 | NM_173490.4 | Proline-rich protein PRP2 | *PRP2* | -4.8 | 0.0001402 | 0.0028988 |
| 490 | NM_002546.2 | Tumor necrosis factor receptor superfamily, member 11b | *TNFRSF11B* | -4.8 | 0.0001814 | 0.0033859 |
| 491 | NM_014601.2 | EH-domain containing 2 | *EHD2* | -4.8 | 2.87E-06 | 0.0002901 |
| 492 | NM_024616.1 | Chromosome 3 open reading frame 52 | *C3ORF52* | -4.8 | 6.36E-05 | 0.0017915 |
| 493 | NM_152330.2 | FERM domain containing 6 | *FRMD6* | -4.9 | 7.16E-06 | 0.0004579 |
| 494 | NM_207376.1 | Hypothetical protein | *LOC387882* | -4.9 | 0.0001345 | 0.0028311 |
| 495 | NM_003246.2 | Thrombospondin 1 | *THBS1* | -4.9 | 0.0002569 | 0.0042254 |
| 496 | NM_152359.1 | Carnitine palmitoyltransferase 1C | *CPT1C* | -4.9 | 0.0001594 | 0.0031475 |
| 497 | NM_006307.2 | Sushi-repeat-containing protein, X-linked | *SRPX* | -4.9 | 0.0028826 | 0.0202472 |
| 498 | NM_002309.2 | Leukemia inhibitory factor | *LIF* | -5.0 | 0.0001343 | 0.0028302 |
| 499 | BX647541 | Mrna; cdna dkfzp686p0492 | *HS.22907* | -5.0 | 1.84E-06 | 0.0002188 |
| 500 | NM_030786.1 | Syncoilin, intermediate filament 1 | *SYNC1* | -5.0 | 0.0009021 | 0.0094372 |
| 501 | NM_032413.2 | Chromosome 15 open reading frame 48 , transcript variant 2 | *C15ORF48* | -5.1 | 0.0008822 | 0.0093016 |
| 502 | NM_014286.2 | Frequenin homolog | *FREQ* | -5.1 | 2.09E-08 | 1.35E-05 |
| 503 | NM_005504.4 | Branched chain aminotransferase 1, cytosolic | *BCAT1* | -5.1 | 0.0015415 | 0.0134283 |
| 504 | BX349502 | BX349502 NEUROBLASTOMA COT 25-NORMALIZED cdna clone CS0DC026YA14 3-PRIME, mrna sequence | *HS.573268* | -5.2 | 0.0006865 | 0.0079746 |
| 505 | NM_001307.3 | Claudin 7 | *CLDN7* | -5.2 | 0.0014387 | 0.012878 |
| 506 | NM_145244.2 | DNA-damage-inducible transcript 4-like | *DDIT4L* | -5.2 | 4.00E-06 | 0.0003402 |
| 507 | NM_005242.3 | Coagulation factor II receptor-like 1 | *F2RL1* | -5.2 | 3.04E-05 | 0.0011369 |
| 508 | NM_152888.1 | Collagen, type XXII, alpha 1 | *COL22A1* | -5.2 | 0.004 | 0.0267152 |
| 509 | NM_020650.2 | Reticulocalbin 3, EF-hand calcium binding domain | *RCN3* | -5.2 | 0.0003563 | 0.0051664 |
| 510 | NM_006670.3 | Trophoblast glycoprotein | *TPBG* | -5.2 | 0.0001131 | 0.0025736 |
| 511 | NM_020443.2 | Neuron navigator 1 | *NAV1* | -5.3 | 1.06E-05 | 0.0005968 |
| 512 | NM_012242.2 | Dickkopf homolog 1 | *DKK1* | -5.3 | 0.005 | 0.0285347 |
| 513 | XM_087386.8 | PREDICTED: HEG homolog 1 | *HEG1* | -5.3 | 0.0021194 | 0.0165971 |
| 514 | NM_006765.2 | Tumor suppressor candidate 3 , transcript variant 1 | *TUSC3* | -5.4 | 4.57E-05 | 0.0014451 |
| 515 | XM_940079.1 | PREDICTED: tubulin, beta 6 | *TUBB6* | -5.4 | 5.25E-06 | 0.0003799 |
| 516 | NM_014262.2 | Leprecan-like 2 | *LEPREL2* | -5.4 | 0.0006605 | 0.0077617 |
| 517 | NM_005727.2 | Tetraspanin 1 | *TSPAN1* | -5.4 | 0.0002854 | 0.0044629 |
| 518 | NM_001797.2 | Cadherin 11, type 2, OB-cadherin | *CDH11* | -5.4 | 0.0025824 | 0.0188694 |
| 519 | NM_006042.1 | Heparan sulfate 3-O-sulfotransferase 3A1 | *HS3ST3A1* | -5.5 | 5.09E-05 | 0.0015571 |
| 520 | NM_000511.3 | Fucosyltransferase 2 | *FUT2* | -5.5 | 0.0017235 | 0.0144137 |
| 521 | NM_016651.4 | Dapper, antagonist of beta-catenin, homolog 1 | *DACT1* | -5.6 | 0.0001941 | 0.0035323 |
| 522 | NM_014632.2 | Microtubule associated monoxygenase, calponin and LIM domain containing 2 | *MICAL2* | -5.6 | 3.39E-06 | 0.0003114 |
| 523 | NM_000358.1 | Transforming growth factor, beta-induced, 68kda | *TGFBI* | -5.6 | 3.30E-09 | 3.41E-06 |
| 524 | NM_001856.2 | Collagen, type XVI, alpha 1 | *COL16A1* | -5.7 | 0.000235 | 0.0040044 |
| 525 | NM_213674.1 | Tropomyosin 2 , transcript variant 2 | *TPM2* | -5.8 | 0.0001356 | 0.0028434 |
| 526 | NM_207380.1 | FLJ43339 protein | *FLJ43339* | -5.8 | 0.0011922 | 0.0114363 |
| 527 | NM_000641.2 | Interleukin 11 | *IL11* | -5.8 | 1.87E-05 | 0.0008391 |
| 528 | NM_002300.3 | Lactate dehydrogenase B | *LDHB* | -5.9 | 1.24E-05 | 0.0006564 |
| 529 | NM_006636.2 | Methylenetetrahydrofolate dehydrogenase 2, methenyltetrahydrofolate cyclohydrolase , nuclear gene encoding mitochondrial protein | *MTHFD2* | -5.9 | 0.0002824 | 0.0044503 |
| 530 | NM_002204.1 | Integrin, alpha 3 , transcript variant a | *ITGA3* | -5.9 | 0.0008116 | 0.0088297 |
| 531 | NM_005429.2 | Vascular endothelial growth factor C | *VEGFC* | -6.0 | 9.19E-05 | 0.002235 |
| 532 | NM_001017974.1 | Procollagen-proline, 2-oxoglutarate 4-dioxygenase , alpha polypeptide II , transcript variant 3 | *P4HA2* | -6.0 | 0.0001066 | 0.0024708 |
| 533 | NM_005110.1 | Glutamine-fructose-6-phosphate transaminase 2 | *GFPT2* | -6.0 | 0.0001551 | 0.0030941 |
| 534 | NM_002167.2 | Inhibitor of DNA binding 3, dominant negative helix-loop-helix protein | *ID3* | -6.1 | 6.03E-05 | 0.0017342 |
| 535 | NM_012334.1 | Myosin X | *MYO10* | -6.2 | 3.52E-05 | 0.0012478 |
| 536 | NM_003330.2 | Thioredoxin reductase 1 , transcript variant 1 | *TXNRD1* | -6.2 | 4.71E-08 | 2.28E-05 |
| 537 | NM_006216.2 | Serpin peptidase inhibitor, clade E , member 2 | *SERPINE2* | -6.2 | 0.0002029 | 0.0036473 |
| 538 | NM_001005376.1 | Plasminogen activator, urokinase receptor , transcript variant 2 | *PLAUR* | -6.3 | 7.08E-06 | 0.0004573 |
| 539 | NM_002145.2 | Homeo box B2 | *HOXB2* | -6.4 | 7.06E-06 | 0.0004573 |
| 540 | NM_006142.3 | Stratifin | *SFN* | -6.4 | 0.0003847 | 0.0053898 |
| 541 | NM_007085.3 | Follistatin-like 1 | *FSTL1* | -6.4 | 6.65E-05 | 0.0018295 |
| 542 | NM_002982.3 | Chemokine ligand 2 | *CCL2* | -6.5 | 0.0002859 | 0.0044629 |
| 543 | NM_001793.3 | Cadherin 3, type 1, P-cadherin | *CDH3* | -6.5 | 0.0003971 | 0.0055065 |
| 544 | NM_024563.2 | Hypothetical protein FLJ14054 | *FLJ14054* | -6.5 | 1.03E-05 | 0.0005931 |
| 545 | NM_002781.2 | Pregnancy specific beta-1-glycoprotein 5 | *PSG5* | -6.5 | 0.0001995 | 0.0036052 |
| 546 | NM_000963.1 | Prostaglandin-endoperoxide synthase 2 | *PTGS2* | -6.6 | 0.0005688 | 0.0070767 |
| 547 | NM_001004019.1 | Fibulin 2 , transcript variant 1 | *FBLN2* | -6.7 | 0.0019008 | 0.0154457 |
| 548 | XM_290629.6 | PREDICTED: chromosome 14 open reading frame 78, transcript variant 1 | *C14ORF78* | -6.7 | 0.0028466 | 0.0201023 |
| 549 | NM_002373.4 | Microtubule-associated protein 1A | *MAP1A* | -6.7 | 0.0002253 | 0.0038856 |
| 550 | NM_015170.1 | Sulfatase 1 | *SULF1* | -6.7 | 0.0018193 | 0.0150029 |
| 551 | NM_206833.1 | Cortexin 1 | *CTXN1* | -6.8 | 0.0002725 | 0.0043499 |
| 552 | NM_000961.3 | Prostaglandin I2 synthase | *PTGIS* | -6.8 | 0.007 | 0.0348792 |
| 553 | NM_000700.1 | Annexin A1 | *ANXA1* | -6.8 | 5.55E-05 | 0.0016366 |
| 554 | NM_000088.2 | Collagen, type I, alpha 1 | *COL1A1* | -6.8 | 0.0019428 | 0.0156847 |
| 555 | NM_021102.2 | Serine peptidase inhibitor, Kunitz type, 2 | *SPINT2* | -6.9 | 0.0037471 | 0.0240893 |
| 556 | NM_177949.1 | Armadillo repeat containing, X-linked 2 | *ARMCX2* | -6.9 | 1.24E-05 | 0.0006564 |
| 557 | NM_021013.3 | Keratin, hair, acidic, 4 | *KRTHA4* | -6.9 | 0.0030052 | 0.020811 |
| 558 | NM_000852.2 | Glutathione S-transferase pi | *GSTP1* | -6.9 | 8.22E-05 | 0.0020704 |
| 559 | NM_005545.3 | Immunoglobulin superfamily containing leucine-rich repeat , transcript variant 1 | *ISLR* | -7.0 | 0.0003634 | 0.0052262 |
| 560 | NM_000602.1 | Serpin peptidase inhibitor, clade E , member 1 | *SERPINE1* | -7.0 | 9.77E-06 | 0.0005693 |
| 561 | NM_144947.1 | Kallikrein 11 , transcript variant 2 | *KLK11* | -7.0 | 0.0001156 | 0.0026044 |
| 562 | NM_001955.2 | Endothelin 1 | *EDN1* | -7.0 | 0.0005319 | 0.0067335 |
| 563 | NM_002147.2 | Homeo box B5 | *HOXB5* | -7.0 | 9.55E-05 | 0.0022932 |
| 564 | NM_001451.1 | Forkhead box F1 | *FOXF1* | -7.1 | 8.77E-05 | 0.0021695 |
| 565 | NM_004750.2 | Cytokine receptor-like factor 1 | *CRLF1* | -7.1 | 5.56E-05 | 0.0016366 |
| 566 | NM_004932.2 | Cadherin 6, type 2, K-cadherin | *CDH6* | -7.2 | 7.71E-06 | 0.0004791 |
| 567 | NM_001450.3 | Four and a half LIM domains 2 , transcript variant 1 | *FHL2* | -7.2 | 5.98E-05 | 0.0017209 |
| 568 | NM_001001991.1 | Regeneration associated muscle protease , transcript variant 2 | *DKFZP586H2123* | -7.2 | 0.0029204 | 0.0204303 |
| 569 | NM_007115.2 | Tumor necrosis factor, alpha-induced protein 6 | *TNFAIP6* | -7.2 | 0.0003898 | 0.005441 |
| 570 | NM_000093.2 | Collagen, type V, alpha 1 | *COL5A1* | -7.3 | 0.0004329 | 0.0058322 |
| 571 | NM_078467.1 | Cyclin-dependent kinase inhibitor 1A , transcript variant 2 | *CDKN1A* | -7.3 | 6.24E-05 | 0.0017715 |
| 572 | NM_080489.2 | Syndecan binding protein 2 , transcript variant 1 | *SDCBP2* | -7.3 | 0.000148 | 0.0029966 |
| 573 | NM_003236.1 | Transforming growth factor, alpha | *TGFA* | -7.5 | 8.34E-05 | 0.0020922 |
| 574 | NM_001017402.1 | Laminin, beta 3 , transcript variant 2 | *LAMB3* | -7.5 | 0.0001397 | 0.0028943 |
| 575 | NM_144617.1 | Heat shock protein, alpha-crystallin-related, B6 | *HSPB6* | -7.5 | 0.001047 | 0.0104751 |
| 576 | NM_002276.3 | Keratin 19 | *KRT19* | -7.5 | 0.0042028 | 0.0259309 |
| 577 | NM_001018008.1 | Tropomyosin 1 , transcript variant 6 | *TPM1* | -7.5 | 1.10E-07 | 3.59E-05 |
| 578 | NM_000584.2 | Interleukin 8 | *IL8* | -7.6 | 0.0004049 | 0.0055887 |
| 579 | NM_133436.1 | Asparagine synthetase , transcript variant 1 | *ASNS* | -7.7 | 0.0002515 | 0.0041752 |
| 580 | XM_927536.1 | PREDICTED: aldehyde dehydrogenase 1 family, member L2 | *ALDH1L2* | -7.9 | 0.0002102 | 0.0037317 |
| 581 | XM_938715.1 | PREDICTED: hyaluronan synthase 2 | *HAS2* | -8.0 | 8.77E-08 | 3.09E-05 |
| 582 | NM_019102.2 | Homeobox A5 | *HOXA5* | -8.1 | 0.0002766 | 0.004379 |
| 583 | NM_020169.2 | Latexin | *LXN* | -8.1 | 0.0007868 | 0.0086466 |
| 584 | NM_004460.2 | Fibroblast activation protein, alpha | *FAP* | -8.2 | 0.0003194 | 0.0047992 |
| 585 | NM_006952.3 | Uroplakin 1B | *UPK1B* | -8.5 | 1.52E-06 | 0.0001907 |
| 586 | XM_934895.1 | PREDICTED: similar to keratin 17, transcript variant 3 | *LOC440421* | -8.6 | 0.0007718 | 0.0085572 |
| 587 | NM_000089.3 | Collagen, type I, alpha 2 | *COL1A2* | -8.6 | 0.0004721 | 0.0061894 |
| 588 | NM_173213.1 | Keratin 23 , transcript variant 2 | *KRT23* | -8.6 | 0.000298 | 0.0045896 |
| 589 | NM_198541.1 | Insulin growth factor-like family member 1 | *IGFL1* | -8.6 | 0.003671 | 0.0237885 |
| 590 | NM_002317.3 | Lysyl oxidase | *LOX* | -8.9 | 0.0006502 | 0.0076889 |
| 591 | XM_937100.1 | PREDICTED: keratin associated protein 2-1, transcript variant 3 | *KRTAP2-1* | -8.9 | 0.0007688 | 0.008545 |
| 592 | NM_001958.2 | Eukaryotic translation elongation factor 1 alpha 2 | *EEF1A2* | -9.0 | 0.0003719 | 0.005281 |
| 593 | NM_001008397.1 | Similar to RIKEN cdna 2310016C16 | *LOC493869* | -9.0 | 1.60E-05 | 0.0007535 |
| 594 | NM_002627.3 | Phosphofructokinase, platelet | *PFKP* | -9.1 | 3.89E-05 | 0.0013148 |
| 595 | NM_080927.3 | Discoidin, CUB and LCCL domain containing 2 | *DCBLD2* | -9.2 | 2.22E-07 | 5.78E-05 |
| 596 | NM_002203.2 | Integrin, alpha 2 | *ITGA2* | -9.4 | 3.76E-06 | 0.0003281 |
| 597 | NM_000930.2 | Plasminogen activator, tissue , transcript variant 1 | *PLAT* | -9.4 | 0.0008494 | 0.0090865 |
| 598 | NM_001458.2 | Filamin C, gamma | *FLNC* | -9.6 | 0.0001259 | 0.0027372 |
| 599 | NM_020404.2 | CD248 antigen, endosialin | *CD248* | -9.6 | 0.0013704 | 0.0125108 |
| 600 | NM_002773.2 | Protease, serine, 8 | *PRSS8* | -9.7 | 0.0002667 | 0.004316 |
| 601 | NM_003012.3 | Secreted frizzled-related protein 1 | *SFRP1* | -10.1 | 2.18E-05 | 0.0009277 |
| 602 | NM_003714.2 | Stanniocalcin 2 | *STC2* | -10.2 | 9.75E-05 | 0.002323 |
| 603 | NM_004472.1 | Forkhead box D1 | *FOXD1* | -10.3 | 0.0002074 | 0.0037001 |
| 604 | NM_006528.2 | Tissue factor pathway inhibitor 2 | *TFPI2* | -10.4 | 0.0023226 | 0.0176485 |
| 605 | NM_018891.1 | Laminin, gamma 2 , transcript variant 2 | *LAMC2* | -10.7 | 0.0001193 | 0.0026583 |
| 606 | NM_006086.2 | Tubulin, beta 3 | *TUBB3* | -10.7 | 0.0008731 | 0.009251 |
| 607 | NM_016307.3 | Paired related homeobox 2 | *PRRX2* | -10.7 | 5.59E-06 | 0.0003956 |
| 608 | NM_013451.2 | Fer-1-like 3, myoferlin , transcript variant 1 | *FER1L3* | -10.8 | 4.82E-05 | 0.0014937 |
| 609 | NM_023938.4 | Chromosome 1 open reading frame 116 | *C1ORF116* | -11.0 | 4.80E-05 | 0.0014913 |
| 610 | NM_001031692.1 | Leucine rich repeat containing 17 , transcript variant 1 | *LRRC17* | -11.0 | 2.73E-06 | 0.000289 |
| 611 | NM_025257.1 | Solute carrier family 44, member 4 , transcript variant 1 | *SLC44A4* | -11.0 | 9.40E-06 | 0.0005581 |
| 612 | NM_001018021.1 | Mucin 1, transmembrane , transcript variant 4 | *MUC1* | -11.6 | 0.0013054 | 0.0121424 |
| 613 | NM_002581.3 | Pregnancy-associated plasma protein A, pappalysin 1 | *PAPPA* | -11.7 | 6.94E-06 | 0.0004563 |
| 614 | NM_014391.2 | Ankyrin repeat domain 1 | *ANKRD1* | -12.9 | 0.0008332 | 0.0089906 |
| 615 | NM_002994.3 | Chemokine ligand 5 | *CXCL5* | -12.9 | 0.0006304 | 0.0075582 |
| 616 | NM_001878.2 | Cellular retinoic acid binding protein 2 | *CRABP2* | -13.1 | 6.36E-06 | 0.0004406 |
| 617 | NM_002658.2 | Plasminogen activator, urokinase | *PLAU* | -13.2 | 0.0001394 | 0.0028923 |
| 618 | NM_052913.2 | Kiaa1913 | *KIAA1913* | -14.2 | 1.68E-06 | 0.0002046 |
| 619 | NM_005139.1 | Annexin A3 | *ANXA3* | -14.4 | 3.57E-05 | 0.0012559 |
| 620 | NM_000600.1 | Interleukin 6 | *IL6* | -14.4 | 0.0001666 | 0.0032325 |
| 621 | NM_000710.2 | Bradykinin receptor B1 | *BDKRB1* | -14.8 | 4.24E-07 | 8.79E-05 |
| 622 | NM_013372.5 | Gremlin 1, cysteine knot superfamily, homolog | *GREM1* | -14.8 | 2.01E-05 | 0.0008751 |
| 623 | NM_020387.1 | RAB25, member RAS oncogene family | *RAB25* | -15.0 | 9.38E-05 | 0.0022678 |
| 624 | NM_005564.2 | Lipocalin 2 | *LCN2* | -15.4 | 9.81E-05 | 0.0023276 |
| 625 | NM_002704.2 | Pro-platelet basic protein ligand 7) | *PPBP* | -15.5 | 0.0023362 | 0.0177152 |
| 626 | NM_001993.2 | Coagulation factor III | *F3* | -16.9 | 1.27E-06 | 0.0001705 |
| 627 | NM_003979.3 | G protein-coupled receptor, family C, group 5, member A | *GPRC5A* | -17.1 | 3.16E-06 | 0.0002997 |
| 628 | NM_005613.3 | Regulator of G-protein signalling 4 | *RGS4* | -17.4 | 9.19E-07 | 0.0001403 |
| 629 | NM_182507.1 | Hypothetical protein LOC144501 | *LOC144501* | -17.6 | 6.09E-05 | 0.0017439 |
| 630 | NM_006818.3 | Myeloid/lymphoid or mixed-lineage leukemia ; translocated to, 11 | *MLLT11* | -18.0 | 1.48E-07 | 4.50E-05 |
| 631 | NM_014070.1 | Chromosome 6 open reading frame 15 | *C6ORF15* | -18.3 | 0.0036805 | 0.0238085 |
| 632 | NM_002852.2 | Pentraxin-related gene, rapidly induced by IL-1 beta | *PTX3* | -18.6 | 8.23E-05 | 0.0020704 |
| 633 | NM_005556.3 | Keratin 7 | *KRT7* | -20.4 | 9.15E-05 | 0.0022323 |
| 634 | NM_153360.1 | Hypothetical protein FLJ90166 | *FLJ90166* | -21.4 | 2.17E-07 | 5.78E-05 |
| 635 | NM_002178.2 | Insulin-like growth factor binding protein 6 | *IGFBP6* | -21.5 | 8.99E-06 | 0.0005419 |
| 636 | NM_002421.2 | Matrix metallopeptidase 1 | *MMP1* | -21.6 | 0.0004068 | 0.0056122 |
| 637 | NM_002483.3 | Carcinoembryonic antigen-related cell adhesion molecule 6 | *CEACAM6* | -22.0 | 5.34E-05 | 0.0016035 |
| 638 | NM_001323.2 | Cystatin E/M | *CST6* | -23.8 | 8.64E-06 | 0.0005246 |
| 639 | NM_000422.1 | Keratin 17 | *KRT17* | -24.2 | 0.0002633 | 0.0042746 |
| 640 | NM_007281.1 | Scrapie responsive protein 1 | *SCRG1* | -25.2 | 2.31E-05 | 0.0009596 |
| 641 | NM_004598.2 | Sparc/osteonectin, cwcv and kazal-like domains proteoglycan | *SPOCK* | -26.3 | 2.41E-07 | 6.00E-05 |
| 642 | NM_004181.3 | Ubiquitin carboxyl-terminal esterase L1 | *UCHL1* | -26.5 | 9.71E-05 | 0.0023172 |
| 643 | NM_153370.2 | Peptidase inhibitor 16 | *PI16* | -30.9 | 2.76E-06 | 0.0002901 |
| 644 | NM_003480.2 | Microfibrillar associated protein 5 | *MFAP5* | -34.9 | 1.00E-10 | 2.90E-07 |
| 645 | NM_002575.1 | Serpin peptidase inhibitor, clade B , member 2 | *SERPINB2* | -40.6 | 1.55E-06 | 0.0001939 |
| 646 | NM_000693.1 | Aldehyde dehydrogenase 1 family, member A3 | *ALDH1A3* | -59.3 | 7.36E-08 | 2.83E-05 |

A univariate *t*-test based on 10,000 random permutations in R packages was used to statistically analyze the differentially expressed genes. Genes with a *q*-value < 0.05 and with a mean difference > 4 were selected.
