# Supplementary material for: Genome-wide expression patterns associated with oncogenesis and sarcomatous transdifferentation of cholangiocarcinoma
Source: BMC Cancer. 2011 Feb 19;11:78. doi: 10.1186/1471-2407-11-78 (PMC3053267; doi:10.1186/1471-2407-11-78)
Supplement: Additional file 4 — Supplementary Table S4: Top 25 genes commonly regulated in both CC cells and tissues compared with cultured biliary epithelial cells. [file 1471-2407-11-78-S4.DOC]

Supplementary Table 4: Top 25 genes commonly regulated in both CC cells and tissues compared with cultured biliary epithelial cells.

| **Rank** | **Accession No.** | **Title** | **Symbol** | **Mean fold change** | ***P* Value** | ***q*-Value** |
| --- | --- | --- | --- | --- | --- | --- |
| A. Genes commonly upregulated in CC cells and tissues | | | | | | |
| 1 | NM_000582.2 | Secreted phosphoprotein 1 (osteopontin, bone sialoprotein I, early T-lymphocyte activation 1) | *SPP1* | 17.69 | 0.0063901 | 0.0657204 |
| 2 | NM_000096.1 | Ceruloplasmin (ferroxidase) | *CP* | 14.76 | 0.004606 | 0.0560517 |
| 3 | NM_000591.1 | CD14 antigen (CD14) | *CD14* | 10.09 | 0.0030821 | 0.0462251 |
| 4 | NM_031311.2 | Carboxypeptidase, vitellogenic-like (CPVL), transcript variant 1, mRNA. | *CPVL* | 8.12 | 0.0005808 | 0.0207333 |
| 5 | NM_016323.1 | Hect domain and RLD 5 | *HERC5* | 5.53 | 0.0004827 | 0.0189638 |
| 6 | NM_004086.1 | coagulation factor C homolog, cochlin (Limulus polyphemus) | *COCH* | 5.38 | 0.0001803 | 0.0119637 |
| 7 | NM_003645.2 | Solute carrier family 27 (fatty acid transporter), member 2 | *SLC27A2* | 5.36 | 0.0048143 | 0.0571476 |
| 8 | NM_016619.1 | Placenta-specific 8 | *PLAC8* | 5.02 | 0.0035658 | 0.0493337 |
| 9 | NM_004120.3 | Binding protein 2, interferon-inducible | *GBP2* | 4.93 | 0.0043132 | 0.0541957 |
| 10 | NM_005258.2 | GTP cyclohydrolase I feedback regulator | *GCHFR* | 4.32 | 0.0048704 | 0.0572591 |
| 11 | NM_004093.2 | Ephrin-B2 | *EFNB2* | 4.15 | 0.0002098 | 0.0128881 |
| 12 | NM_018490.1 | Leucine-rich repeat-containing G protein-coupled receptor 4 | *LGR4* | 4.13 | 0.0017499 | 0.0345578 |
| 13 | NM_020247.3 | Chaperone, ABC1 activity of bc1 complex like (S. pombe | *CABC1* | 3.91 | 0.0062336 | 0.0649723 |
| 14 | NM_014234.3 | Hydroxysteroid (17-beta) dehydrogenase 8. | *HSD17B8* | 3.90 | 0.0027659 | 0.0437959 |
| 15 | BQ438671 | cDNA clone IMAGE:6102595 5 |  | 3.73 | 0.0088979 | 0.0765079 |
| 16 | NM_006408.2 | Gradient 2 homolog (Xenopus laevis) | *AGR2* | 3.62 | 0.0013778 | 0.0309093 |
| 17 | NM_014573.1 | Transmembrane protein 97 | *TMEM97* | 3.56 | 0.0027974 | 0.0439756 |
| 18 | BC035116 | cDNA clone IMAGE:5263177 |  | 3.54 | 0.0046531 | 0.0563239 |
| 19 | NM_007129.2 | Zic family member 2 (odd-paired homolog, Drosophila) | *ZIC2* | 3.47 | 1.21E-05 | 0.0031677 |
| 20 | NM_148918.1 | Serine hydroxymethyltransferase 1 (soluble) (SHMT1), transcript variant 2 | *SHMT1* | 3.45 | 2.27E-06 | 0.0012335 |
| 21 | NM_199335.2 | FYN binding protein (FYB-120/130) (FYB), transcript variant 2 | *FYB* | 3.31 | 0.0073894 | 0.0698031 |
| 22 | NM_004091.2 | E2F transcription factor 2 (E2F2) | *E2F2* | 3.16 | 0.0005596 | 0.0203305 |
| 23 | NM_001467.3 | Solute carrier family 37 (glycerol-6-phosphate transporter), member 4 | *SLC37A4* | 3.09 | 0.0042853 | 0.053963 |
| 24 | NM_014214.1 | Inositol(myo)-1(or 4)-monophosphatase 2 | *IMPA2* | 2.99 | 0.0093063 | 0.0782639 |
| 25 | NM_153259.2 | mucolipin 2 | *MCOLN2* | 2.93 | 0.0004289 | 0.017726 |
| B. Genes commonly downregulated in CC cells and tissues | | | | | | |
| 1 | NM_000089.3 | Collagen, type I, alpha 2 | *COL1A2* | -167.85 | 1.10E-09 | 9.34E-06 |
| 2 | NM_000088.2 | Collagen, type I, alpha 1 | *COL1A1* | -126.15 | 1.90E-09 | 1.09E-05 |
| 3 | NM_000422.1 | Keratin 17 | *KRT17* | -68.04 | 9.16E-07 | 0.0009882 |
| 4 | NM_020404.2 | CD248 antigen, endosialin | *CD248* | -72.29 | 2.95E-06 | 0.0013253 |
| 5 | NM_002575.1 | Serpin peptidase inhibitor, clade B (ovalbumin), member 2 | *SERPINB2* | -40.39 | 2.79E-06 | 0.0012916 |
| 6 | NM_003480.2 | Microfibrillar associated protein 5 | *MFAP5* | -44.96 | 0 | 0 |
| 7 | NM_057164.2 | Collagen, type VI, alpha 3 (COL6A3), transcript variant 2 | *COL6A3* | -66.87 | 1.70E-08 | 6.11E-05 |
| 8 | NM_002421.2 | Matrix metallopeptidase 1 (interstitial collagenase) | *MMP1* | -48.34 | 4.38E-05 | 0.0062346 |
| 9 | NM_153370.2 | Peptidase inhibitor 16 | *PI16* | -27.45 | 7.49E-06 | 0.0024464 |
| 10 | NM_015170.1 | Sulfatase 1 | *SULF1* | -45.01 | 6.21E-06 | 0.0021778 |
| 11 | NM_000602.1 | Serpin peptidase inhibitor, clade E (nexin, plasminogen activator inhibitor type 1), member 1 | *SERPINE1* | -38.31 | 2.25E-06 | 0.0012335 |
| 12 | NM_007281.1 | Scrapie responsive protein 1 | *SCRG1* | -25.85 | 3.81E-05 | 0.0059299 |
| 13 | NM_000093.2 | Collagen, type V, alpha 1 | *COL5A1* | -42.25 | 1.51E-05 | 0.0036181 |
| 14 | NM_001001522.1 | Transgelin (TAGLN), transcript variant 1 | *TAGLN* | -44.43 | 2.40E-09 | 1.15E-05 |
| 15 | NM_052913.2 | KIAA1913 | *KIAA1913* | -36.01 | 6.50E-06 | 0.0022317 |
| 16 | NM_002982.3 | Chemokine (C-C motif) ligand 2 | *CCL2* | -36.57 | 1.01E-06 | 0.0009882 |
| 17 | NM_004181.3 | Ubiquitin carboxyl-terminal esterase L1 (ubiquitin thiolesterase) | *UCHL1* | -12.57 | 0.0074468 | 0.0700806 |
| 18 | NM_005613.3 | Regulator of G-protein signalling 4 | *RGS4* | -22.79 | 7.78E-07 | 0.0009717 |
| 19 | NM_001797.2 | Cadherin 11, type 2, OB-cadherin (osteoblast) | *CDH11* | -32.04 | 2.80E-07 | 0.0006194 |
| 20 | NM_153360.1 | Hypothetical protein FLJ90166 | *FLJ90166* | -17.11 | 0.0002138 | 0.0130733 |
| 21 | NM_001323.2 | Cystatin E/M | *CST6* | -16.84 | 3.86E-05 | 0.0059308 |
| 22 | NM_000600.1 | Interleukin 6 (interferon, beta 2) | *IL6* | -17.08 | 4.87E-05 | 0.0063928 |
| 23 | NM_004460.2 | Fibroblast activation protein, alpha | *FAP* | -25.51 | 1.06E-06 | 0.0009882 |
| 24 | NM_004598.2 | Sparc/osteonectin, cwcv and kazal-like domains proteoglycan (testican) | *SPOCK* | -14.05 | 0.0001902 | 0.0122166 |
| 25 | NM_004385.2 | Sulfate proteoglycan 2 (versican) | *CSPG2* | -28.33 | 2.96E-05 | 0.0050555 |

A univariate *t*-test based on 10,000 random permutations in R packages was used to statistically analyze the differentially expressed genes. Genes with a *q*-value < 0.1 and with a mean difference > 2 were selected.
