# Supplementary material for: Genome-wide expression patterns associated with oncogenesis and sarcomatous transdifferentation of cholangiocarcinoma
Source: BMC Cancer. 2011 Feb 19;11:78. doi: 10.1186/1471-2407-11-78 (PMC3053267; doi:10.1186/1471-2407-11-78)
Supplement: Additional file 5 — Supplementary Figure S1: Immunohistochemical staining with anti-SPP1, anti-EFNB2, anti-E2F2, and anti-IRX3 in hamster CC tissues induced by Clonorchiasis infestation. Control stainings were performed in normal hamster livers. *, selected from only cell-based microarray database. 35 [file 1471-2407-11-78-S5.PDF]

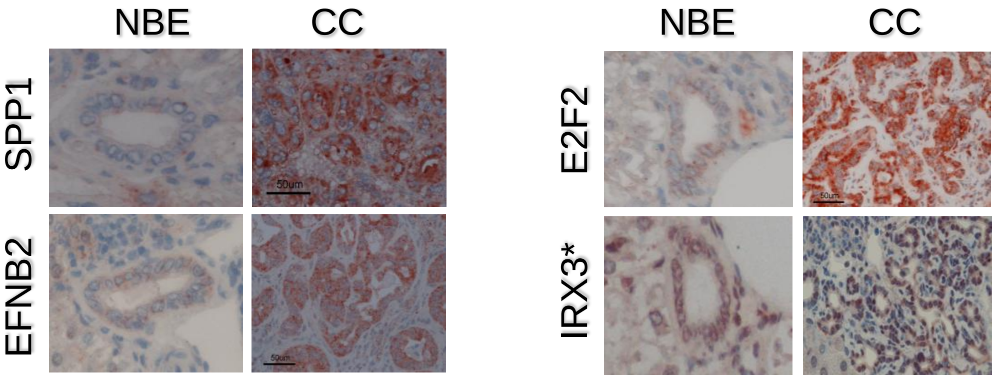

Supplementary Figure 1
Immunohistochemical staining with anti-SPP1, anti-EFNB2, anti-E2F2, and anti-IRX3 in hamster CC tissues induced by *Clonorchiasis* infestation. Control stainings were performed in normal hamster livers. *, selected from only cell-based microarray database.
