# Supplementary material for: Genome-wide expression patterns associated with oncogenesis and sarcomatous transdifferentation of cholangiocarcinoma
Source: BMC Cancer. 2011 Feb 19;11:78. doi: 10.1186/1471-2407-11-78 (PMC3053267; doi:10.1186/1471-2407-11-78)
Supplement: Additional file 6 — Supplementary Table 5. Top 25 genes differentially expressed in the sarcomatoid SCK cells compared with 3 adenocarcinomatous CC cell lines. [file 1471-2407-11-78-S6.DOC]

Supplementary Table 5: Top 25 genes differentially expressed in the sarcomatoid SCK cells compared with 3 adenocarcinomatous CC cell lines.

| **Rank** | **Accession No.** | **Title** | **Symbol** | **Mean fold change** | ***P* value** | ***q*-value** |
| --- | --- | --- | --- | --- | --- | --- |
| A. Genes differentially upregulated in SCK cells compared with 3 CC cell lines | | | | | | |
| 1 | NM_002300.3 | Lactate dehydrogenase B | *LDHB* | 130.6 | 0 | 0 |
| 2 | NM_004063.2 | Cadherin 17, LI cadherin (liver-intestine) | *CDH17* | 89.2 | 0 | 0 |
| 3 | NM_001477.1 | G antigen 7B | *GAGE7B* | 85.2 | 0 | 0 |
| 4 | NM_001474.1 | G antigen 4 | *GAGE4* | 82.3 | 0 | 0 |
| 5 | NM_012196.1 | G antigen 8 | *GAGE8* | 80.2 | 0 | 0 |
| 6 | NM_001017436.1 | Cancer/testis antigen CT45-4 | *CT45-4* | 80.0 | 0 | 0 |
| 7 | NM_001475.1 | G antigen 5 | *GAGE5* | 76.4 | 0 | 0 |
| 8 | NM_001472.1 | Homo sapiens G antigen 2 | *GAGE2* | 73.1 | 0 | 0 |
| 9 | NM_001252.2 | Tumor necrosis factor (ligand) superfamily, member 7 | *TNFSF7* | 60.1 | 0 | 0 |
| 10 | NM_001476.1 | G antigen 6 . | *GAGE6* | 51.4 | 0 | 0 |
| 11 | NM_000667.2 | Alcohol dehydrogenase 1A (class I), alpha polypeptide | *ADH1A* | 49.2 | 4.00E-10 | 8.35E-09 |
| 12 | NM_002133.1 | Heme oxygenase (decycling) 1 | *HMOX1* | 46.5 | 0 | 0 |
| 13 | NM_001017435.1 | Cancer/testis antigen CT45-3 | *CT45-3* | 45.8 | 0 | 0 |
| 14 | NM_002523.1 | Neuronal pentraxin II | *NPTX2* | 39.5 | 0 | 0 |
| 15 | NM_020299.3 | Aldo-keto reductase family 1, member B10 (aldose reductase). | *AKR1B10* | 39.3 | 3.41E-08 | 3.65E-07 |
| 16 | NM_199051.1 | Family with sequence similarity 5, member C | *FAM5C* | 35.8 | 0 | 0 |
| 17 | NM_000669.3 | Alcohol dehydrogenase 1C (class I), gamma polypeptide | *ADH1C* | 35.0 | 9.00E-10 | 1.68E-08 |
| 18 | NM_004052.2 | BCL2/adenovirus E1B 19kda interacting protein 3 | *BNIP3* | 32.7 | 0 | 0 |
| 19 | NM_016613.4 | Chromosome 4 open reading frame 18 | *C4orf18* | 30.1 | 0 | 0 |
| 20 | XM_930008.1 | Similar to GAGE-2 protein (G antigen 2) | *LOC645037* | 27.6 | 0 | 0 |
| 21 | NM_000096.1 | Ceruloplasmin (ferroxidase) | *CP* | 27.0 | 0 | 0 |
| 22 | NM_013301.1 | Protein predicted by clone 23882 | *HSU79303* | 26.4 | 0 | 0 |
| 23 | NM_004181.3 | Ubiquitin carboxyl-terminal esterase L1 (ubiquitin thiolesterase) | *UCHL1* | 26.2 | 7.35E-08 | 7.04E-07 |
| 24 | NM_002727.2 | Proteoglycan 1, secretory granule | *PRG1* | 25.9 | 0 | 0 |
| 25 | NM_001017417.1 | Cancer/testis antigen CT45-1 | *CT45-1* | 25.3 | 0 | 0 |
| B. Genes differentially downregulated in SCK cells compared with 3 CC cell lines | | | | | | |
| 1 | NM_052886.1 | Mal, T-cell differentiation protein 2 | *MAL2* | -87.0 | 0 | 0 |
| 2 | NM_005564.2 | Lipocalin 2 (oncogene 24p3) | *LCN2* | -86.1 | 1.04E-08 | 1.33E-07 |
| 3 | NM_007231.1 | Solute carrier family 6 (amino acid transporter), member 14 | *SLC6A14* | -80.8 | 1.00E-10 | 2.51E-09 |
| 4 | NM_000693.1 | Aldehyde dehydrogenase 1 family, member A3 | *ALDH1A3* | -72.4 | 0 | 0 |
| 5 | NM_005980.2 | S100 calcium binding protein P | *S100P* | -67.5 | 1.00E-10 | 2.51E-09 |
| 6 | NM_023938.4 | Chromosome 1 open reading frame 116 | *C1orf116* | -50.0 | 0 | 0 |
| 7 | NM_001008.3 | Ribosomal protein S4, Y-linked 1 | *RPS4Y1* | -48.5 | 4.00E-10 | 8.35E-09 |
| 8 | NM_021101.3 | Claudin 1 | *CLDN1* | -48.3 | 4.03E-08 | 4.21E-07 |
| 9 | NM_005139.1 | Annexin A3. | *ANXA3* | -47.4 | 0 | 0 |
| 10 | NM_020387.1 | RAB25, member RAS oncogene family | *RAB25* | -46.7 | 0 | 0 |
| 11 | NM_006472.1 | Thioredoxin interacting protein | *TXNIP* | -45.5 | 0 | 0 |
| 12 | NM_001062.2 | Transcobalamin I (vitamin B12 binding protein, R binder family) | *TCN1* | -42.3 | 3.00E-10 | 6.51E-09 |
| 13 | NM_138768.2 | Myeloma overexpressed gene (in a subset of t(11;14) positive multiple myelomas) | *MYEOV* | -42.0 | 3.00E-10 | 6.51E-09 |
| 14 | NM_002423.3 | Matrix metallopeptidase 7 (matrilysin, uterine) | *MMP7* | -41.2 | 4.64E-07 | 3.34E-06 |
| 15 | NM_005555.2 | Keratin 6B. | *KRT6B* | -40.6 | 2.30E-09 | 3.72E-08 |
| 16 | NM_002774.3 | Kallikrein 6 (neurosin, zyme) variant A | *KLK6* | -36.8 | 1.10E-09 | 1.98E-08 |
| 17 | NM_201397.1 | Glutathione peroxidase 1. | *GPX1* | -34.2 | 0 | 0 |
| 18 | NM_001001552.3 | LEM domain containing 1 | *LEMD1* | -34.2 | 3.10E-07 | 2.37E-06 |
| 19 | NM_004496.2 | Forkhead box A1 | *FOXA1* | -34.0 | 0 | 0 |
| 20 | NM_002639.2 | Serpin peptidase inhibitor, clade B (ovalbumin), member 5 | *SERPINB5* | -33.7 | 4.75E-08 | 4.83E-07 |
| 21 | NM_005558.3 | Ladinin 1 | *LAD1* | -32.5 | 0 | 0 |
| 22 | NM_000584.2 | Interleukin 8 | *IL8* | -31.1 | 1.00E-10 | 2.51E-09 |
| 23 | NM_174911.3 | Family with sequence similarity 84, member B | *FAM84B* | -30.4 | 0 | 0 |
| 24 | NM_001511.1 | Chemokine (C-X-C motif) ligand 1 | *CXCL1* | -27.6 | 0 | 0 |
| 25 | NM_006017.1 | Prominin 1 | *PROM1* | -27.2 | 2.07E-08 | 2.39E-07 |

A univariate *t*-test based on 10,000 random permutations in R packages was used to statistically analyze the differentially expressed genes. Genes with a *q*-value < 0.05 and with a mean difference > 4 between the two groups were selected.
